# B cell immune profiles in dysbiotic vermiform appendixes of pancreatic cancer patients

**DOI:** 10.3389/fimmu.2023.1230306

**Published:** 2023-11-10

**Authors:** Eveline E. Vietsch, Diba Latifi, Maaike Verheij, Elise W.A. van der Oost, Roeland F. de Wilde, Roel Haen, Anne Loes van den Boom, Bas Groot Koerkamp, Pascal G. Doornebosch, Victorien M.T. van Verschuer, Ariadne H.A.G. Ooms, Farzana Mohammad, Marcella Willemsen, Joachim G.J.V. Aerts, Ricki T. Krog, Noel F.C.C. de Miranda, Thierry P.P. van den Bosch, Yvonne M. Mueller, Peter D. Katsikis, Casper H.J. van Eijck

**Affiliations:** ^1^ Department of Pulmonary Medicine, Erasmus MC Cancer Institute, Rotterdam, Netherlands; ^2^ Department of Surgery, Erasmus MC Cancer Institute, Rotterdam, Netherlands; ^3^ Department of Surgery, Reinier de Graaf Hospital, Delft, Netherlands; ^4^ Department of Surgery, IJsselland Hospital, Capelle aan den IJssel, Netherlands; ^5^ Department of Pathology, Pathan BV, Rotterdam, Netherlands; ^6^ Department of Pathology, Erasmus University Medical Center, Rotterdam, Netherlands; ^7^ Department of Pathology, Leiden University Medical Center, Leiden, Netherlands; ^8^ Department of Immunology, Erasmus University Medical Center, Rotterdam, Netherlands

**Keywords:** vermiform appendix, pancreatic cancer, colon cancer, mucosal B cells, gut associated lymphoid tissue, gut microbiome, HLA-G, HLA-DR

## Abstract

Pancreatic ductal adenocarcinoma (PDAC) remains one of the deadliest solid tumors and is resistant to immunotherapy. B cells play an essential role in PDAC progression and immune responses, both locally and systemically. Moreover, increasing evidence suggests that microbial compositions inside the tumor, as well as in the oral cavity and the gut, are important factors in shaping the PDAC immune landscape. However, the gut-associated lymphoid tissue (GALT) has not previously been explored in PDAC patients. In this study, we analyzed healthy vermiform appendix (VA) from 20 patients with PDAC and 32 patients with colon diseases by gene expression immune profiling, flow cytometry analysis, and microbiome sequencing. We show that the VA GALT of PDAC patients exhibits markers of increased inflammation and cytotoxic cell activity. In contrast, B cell function is decreased in PDAC VA GALT based on gene expression profiling; B cells express significantly fewer MHC class II surface receptors, whereas plasma cells express the immune checkpoint molecule HLA-G. Additionally, the vermiform appendix microbiome of PDAC patients is enriched with *Klebsiella pneumoniae*, *Bifidobacterium animalis*, and *Adlercreutzia equolifaciens*, while certain commensals are depleted. Our findings may suggest impaired B cell function within the GALT of PDAC patients, which could potentially be linked to microbial dysbiosis. Additional investigations are imperative to validate our observations and explore these potential targets of future therapies.

## 1 Introduction

Pancreatic ductal adenocarcinoma (PDAC) is one of the most fatal solid tumors and is notorious for its resistance to (immuno)therapy. Although PDAC exhibits an excessive inflammatory signature, effective immune responses directed at the cancer cells are compromised (1), for reasons incompletely understood. Recently, the role of the lymphoid tissues in shaping cancer immune landscapes has gained interest. Tumor-draining lymph nodes, for example, display immune suppressive signatures in melanoma patients with distant disease recurrence (2). Similarly, increased abundance of regulatory T cells (Tregs) in tumor-draining lymph nodes of lung cancer patients seems predictive of an unfavorable treatment response and clinical outcome (3). The immune landscape of secondary lymphoid tissues in PDAC patients has not been completely elucidated.

The human vermiform appendix (VA) is a diverticulum attached to the cecum. The VA is rich in gut-associated lymphoid tissue (GALT), which maintains the intestinal microflora composition, detects pathogens, regulates immune functions, and shapes the B lymphocyte and primary antibody repertoires (4–6). Lymphocytes differentiate in the GALT, before they are conveyed into the bloodstream and relocated to other organs (7, 8). The primary destination is the liver, as the gut and liver communicate bidirectionally via biliary, portal, and systemic circulation (9).

The VA contains a reservoir of the commensal gut microbiome, capable of repopulating the gut after pathological events in the colon (10). Dysbiosis can cause an imbalance of the mucosal immune homeostasis, leading to the translocation of bacteria and bacterial products to the liver or pancreas. Microbial composition is a crucial factor shaping the PDAC immune landscape, given the known presence of bacterial and fungal components in PDAC tumors and the open connection between the gut and pancreatic duct (11, 12). Others have shown by spatial transcriptomics and single-cell RNA sequencing that microbiota can alter the biology of distinct cellular compartments, affecting antitumor immunity (13). Microbial dysbiosis is found in PDAC tumors and extends beyond the local PDAC environment, resulting in specific PDAC-associated oral and fecal microbial signatures, independent of technical sequencing batch effects, the lifestyle, geography, or genetic backgrounds of the patient populations (14).

The GALT is unexplored territory in PDAC patients, yet it may harbor key insights into PDAC pathogenesis and the mechanisms by which PDAC evades immune surveillance. In this study, we evaluate the immune landscape and the microbiome of the VA GALT of PDAC patients in comparison to patients with benign and malignant colon disease.

## 2 Materials and methods

### 2.1 Patient recruitment and sample collection

PDAC patients were recruited from the Department of Surgery at the Erasmus Medical Center Rotterdam, and informed consent was obtained. Inclusion criteria were age ≥40 years, histopathologically proven PDAC, laparoscopy and/or a tumor resection as part of clinical care for PDAC, and having a healthy vermiform appendix (termed VA sana) *in situ*. Exclusion criteria were previous appendectomy; appendix pathology; radio/chemotherapy in the past 5 years; malignant disease in the past 5 years prior to PDAC diagnosis; pregnancy; immunosuppressive therapy in the past 3 months; and serious concomitant systemic disorders that would compromise the safety of the patient or the ability to complete the study. The Netherlands Trial Registry number is NL7553.

Control formalin-fixed paraffin-embedded (FFPE) VA samples from benign colon disease and colon cancer patients were collected retrospectively from the Department of Pathology at the Erasmus MC and the Reinier de Graaf Gasthuis hospital in Delft from the FFPE tissue archive. The VAs were evaluated by clinical pathologists and defined as noninflamed and without malignancy (termed sana). Inclusion criteria were age ≥40 years; VA *in situ*; benign colon disease; or primary colon adenocarcinoma. Patients in the benign colon disease group were diagnosed with either slow transit obstipation, ileus, megacolon, diverticulitis, benign mesothelial cyst, or suspicion of appendicitis, which proved healthy upon histopathological analysis. Exclusion criteria were equally applied as to the PDAC patients. The studies were approved by the Medical Ethical Committee of the Erasmus MC Rotterdam (MEC-2018-1510 and MEC-2019-0156).

Fresh VA samples from colon adenocarcinoma (colon AC) patients were collected at the IJsselland Hospital and the Erasmus Medical Center in Rotterdam (MEC-2020-0689). Inclusion criteria were age ≥40 years, VA *in situ*, and treatment-naïve colon adenocarcinoma (AC) for which (ileo)cecal resection was indicated as standard clinical care. Exclusion criteria were objection to the use of remaining tissue for research purposes; appendix pathology; other colon cancers than adenocarcinoma; pregnancy; malignant disease and or radio/chemotherapy in the past 10 years; systemic immunosuppressive therapy in the past 3 months; and ASA Physical status IV–VI. Before the VA were processed for research purposes, a clinical pathologist evaluated the gross anatomy, confirmed the absence of VA pathology visible by eye, and collected tissue for clinical histopathology assessment.

### 2.2 FFPE VA RNA isolation

The FFPE VA were sectioned (10 × 10 µm thick per patient sample) and deparaffinized in xylene for 2 min, 90% ethanol for 2 min, 70% ethanol for 2 min, and distilled water for 4 min. After the slides dried for 2 h, regions with mucosa and submucosa of the VA were scraped with a clean scalpel into 1.5 mL microcentrifuge tubes. RNA was isolated using the RNeasy FFPE Kit (QIAGEN, 73504, Hilden, Germany), following the manufacturer’s instructions. RNA was eluted in 17 µL nuclease-free water. The RNA integrity and concentration were measured using the Bioanalyzer RNA 6000 Nano assay (Agilent, 5067-1511, Santa Clara, CA, USA). Only samples with >12% of RNA fragments longer than 300 nucleotides and a scaled concentration above 42.8 ng/µL were used for gene expression profiling.

### 2.3 nCounter immune profiling

Gene expression quantification was performed using the nCounter PanCancer Immune Profiling Panel (NanoString, XTCSO-HIP1-12, Seattle, WA, USA), which contains 730 immune target genes and up to 40 reference genes. Between 310 and 320 ng of RNA (adjusted for RNA fragments >300 nucleotides) per sample was processed following the nCounter FLEX System (NanoString, GLMX_ST0002). RNA was hybridized with capture and reporter probes at 65°C for 17 h in a SimpliAmp Thermal Cycler (Applied Biosystems, Foster City, CA, USA) before loading the samples into the nCounter FLEX system. Image acquisition was performed using the nCounter Digital Analyzer 5s (NanoString) by scanning 490 fields of view. mRNA counts were extracted from the RCC files using nSolver analysis software v4.0 (NanoString). Gene expression was normalized using the most stable reference genes from the panel, selected by the geNorm algorithm incorporated in the Advanced Analysis module of nSolver software v2.0 (NanoString). Cell scores are calculated by the nSolver software, based on normalized expression of the gene characteristic of specific cell types and concordance <0.05: BLK, CD19, and MS4A1 for B cells; GZMB, PRF1, KLRK1, GZMH, KLRD1, and GZMA for cytotoxic cells; XCL2, NCR1, KIR3DL1, and KIR2DL2 for NK cells. Normalized gene counts and cell scores were exported as.csv files for further analysis.

### 2.4 Immunohistochemical staining

Immunohistochemistry was performed with an automated, validated, and accredited staining system (Ventana Benchmark ULTRA, Ventana Medical Systems, Tucson, AZ, USA) using an optiview universal detection kit (Ventana, 760-500). In brief, following deparaffinization and heat-induced antigen retrieval, the tissue sections were incubated with a mouse anti-HLA-G antibody (Santa Cruz, SC-21799, clone 4H84, Dallas, TX, USA) in a 1/100 dilution for 32 min at 37°C, and anti-HLA-DR antibody (DAKO, M0775, clone CRs/43, dilution 1/400, Glostrup, Denmark). 3,3′-Diaminobenzidine tetrahydrochloride (DAB) was used for staining. Incubation was followed by a hematoxylin II counter stain for 12 min and then a blue coloring reagent for 8 min according to the manufacturer’s instructions (Ventana). The slides were scanned using the Nanozoomer 2.0-HT slide imager (Hamamatsu Photonics, Hamamatsu City, Japan). Images at ×10 magnification from surrounding areas of three germinal centers per patient sample were exported to tiff. files. From the images, DAB brown intensity was quantified with ImageJ 1.51n software (Fiji), using the color deconvolution, H DAB vector option.

### 2.5 Automated multiplex immunofluorescent staining

The automated multiplex immunofluorescence (IF) staining for HLA-G, CD68, Pan cytokeratin, CD4, CD8, CD138, and CD79a was done using the Ventana Benchmark Discovery ULTRA (Ventana Medical Systems Inc.) on human appendix and placenta FFPE tissue sections of 4 µm thick in multiple different antibody panels, including HLA-G. In one panel, sections were stained for CD4 (Ventana clone SP35, 790, 4429), CD8 (Ventana clone SP57, 790-4460), and CD68 (Ventana clone KP1, 790, 2931). In a second panel, tissues were also stained for pan-keratin (Ventana clone AE1/AE3/PCK26, 790-2135), HLA-DR (DAKO, clone M0746), and CD68 (Ventana, clone KP1, 790, 2931). Following deparaffinization and heat-induced antigen retrieval with CC1 (Ventana, 950-224) for 32 min, anti-CD68 was incubated for 32 min at 37°C, followed by omnimap anti-mouse HRP (Ventana,760-4310) and detection with DCC (Ventana, 760-240) for 8 min. An antibody denaturation step was performed with CC2 (Ventana, 950-123) at 100°C for 20 min. Secondly, incubation with either anti-CD4 or HLA-DR was performed for 60 min at 37°C, followed by omnimap antimouse HRP (Ventana, 760-4311) and detection with Cy5 (Ventana, 760-238) for 8 min. An antibody denaturation step followed by CC2 at 100°C for 20 min. Thirdly, either anti-CD8 or pan-keratin was incubated for 32 min/4 min at 37°C, followed by omnimap anti-rabbit HRP/Omnimap antimouse (Ventana, 760-4311, 760-3210) and detection with detection with FAM (Ventana,760-243). Finally, slides were washed in phosphate-buffered saline and mounted with Vectashield containing 4′,6-diamidino-2-phenylindole (Vector Laboratories, Peterborough, UK).

An additional panel was performed according to this method: following deparaffinization and heat-induced antigen retrieval with CC1 (Ventana, 950-500) for 64 min at 97°C, the tissue samples were incubated firstly with a mouse anti-HLA-G antibody (GeneTex, GTX78394 clone MEM-G/2, 1/100 dilution) for 32 min at 37°C, followed by detection with Ultramap antimouse HRP (Ventana, 760-4313), followed by visualization with R6G for 8 min (Ventana, 760-243). An antibody denature step was performed using CC2 (Ventana, 950-123) for 20 min at 100°C. Secondly, a mouse anti-CD138 antibody (Cell Marque, 138M, clone B-A38, 0.10 μg/mL, Rocklin, CA, USA) was incubated for 28 min at 37°C, followed by detection with Ultramap antirabbit HRP (Ventana, 760-4315), followed by visualization with Cy5 (Ventana, 760-238). An antibody denature step was performed using CC2 (Ventana, 950-123) for 20 min at 100°C. Thirdly, a rabbit anti-CD79a antibody (Ventana, 790-4432 clone SP18, 0.33 µg/mL) was incubated for 32 min at 37°C, followed by detection with Ultramap antimouse HRP (Ventana, 760-4313) for 12 min, followed by visualization with FAM for 12 min (Ventana, 760-235).

For the multiplex IF panel of CD79a, CD138, HLA-DR, and CD38, the following method was used. Following deparaffinization and heat-induced antigen retrieval with CC1 (Ventana, 950-500) for 64 min at 97°C, the tissue samples were incubated first with the anti-CD79a antibody (Ventana, 790-4432 clone SP18, 0.33 µg/mL) for 32 min at 37°C, followed by detection with Ultramap antimouse HRP (Ventana, 760-4313) for 12 min, followed by visualization with R6G for 8 min (Ventana, 760-243). An antibody denature step was performed using CC2 (Ventana, 950-123) for 20 min at 100°C. Secondly, the slide was incubated with anti-CD138 antibody (Cell Marque, 138M, clone B-A38, 0.10 µg/mL) for 28 min at 37°C, followed by detection with Ultramap anti-rabbit HRP (Ventana, 760-4315), followed by visualization with DCC for 8 min (Ventana, 760-240). An antibody denature step was performed using CC2 (Ventana, 950-123) for 20 min at 100°C. Thirdly, anti-HLA-DR antibody (DAKO, M0775, clone:CR3/43, dilution 1/400) was incubated for 32 min at 37°C, followed by detection with Ultramap anti-mouse HRP (Ventana, 760-4313) for 12 min, followed by visualization with Red610 for 8 min (Ventana, 760-245). An antibody denature step was performed using CC2 (Ventana, 950-123) for 20 min at 100°C. Lastly, anti-CD38 (Cell Marque, 118R-18, SP149, 0.3 µg/mL) was incubated for 32 min at 37°C, followed by detection with Ultramap antimouse HRP (Ventana, 760-4313) for 12 min, followed by visualization with Cy5 (Ventana, 760-238).

All slides were incubated in phosphate-buffered saline with DAPI for 15 min and covered with antifading medium (DAKO, S3023). Confocal microscopy was performed using an LSM-700 laser scanning confocal microscope (Carl Zeiss, Oberkochen, Germany).

### 2.6 Imaging mass cytometry staining and acquisition

Imaging mass cytometry (IMC) was utilized to analyze the immune microenvironment in the appendix, with a focus on the B-cell lineage, of patients with either PDAC (*n* = 3), colon adenocarcinoma (*n* = 3), or noncancer patients (benign colon disease) (*n* = 3). Three regions of interest (ROIs) per patient were included for the inclusion of different areas within the appendix. Antibodies were obtained in a formulation consisting solely of PBS and conjugated to purified lanthanide metals using the Maxpar Antibody Labeling Kit and protocol (Standard BioTools, South San Francisco, CA, USA). Antibody conjugation to 194Pt and 198Pt was performed following the methodology of Mei et al. (15). Metal-conjugated antibodies were tested with immunohistochemistry (IHC) to ensure that the metal conjugation would not affect the performance of the antibody. The final metal-conjugated antibodies were stored in W-buffer (Standard BioTools, CA, USA) and Antibody Stabilizer (CANDOR Bioscience, DE, Wangen im Allgäu, Germany) supplemented with 0.05% sodium azide (1:1). A final panel of 36 markers was established for the staining of the FFPE tissues and was carried out as previously described by Ijsselsteijn et al. (16) (**Supplementary Table S1**). In brief, deparaffinization of the tissues was carried out in xylene, followed by rehydration in decreasing concentrations of ethanol. Antigen retrieval of the tissues was performed in 1× Antigen Retrieval Solution (ThermoFisher Scientific, Waltham, MA, USA) at pH 6, and unspecific staining was blocked using SuperBlock (PBS) Blocking Buffer (ThermoFisher Scientific). Antibodies detected indirectly were incubated overnight at room temperature, followed by incubation with the secondary antibodies conjugated to metals for 1 h at room temperature. Antibodies conjugated to metal for direct detection were incubated either for 5 h at room temperature or overnight at 4°C. Lastly, Cell-ID Intercalator Iridium (125 µM) (Standard BioTools) was incubated for 5 min at room temperature for the detection of DNA. The slides were air-dried and stored at room temperature in vacuum until acquisition.

The acquisition of the tissue ROIs was carried out using the Hyperion Imaging System (Standard BioTools). Autotuning was performed using a three-element tuning slide (Standard BioTools) in concordance with the Hyperion Imaging Systems User Guide (Standard BioTools). The optimal laser strength was determined prior to acquisition of the ROIs. ROIs were ablated at 200 Hz, and images as output were analyzed. All images were visually inspected using the Fluidigm MCD viewer software and exported as single-marker tiff. files. Data were scaled between 0 and 1 and normalized using semiautomatic background removal, after which single-cell information was generated as described previously (17). After dimensionality reduction, single-cell phenotypes were identified through mean-shift clustering in Cytosplore. All clusters were mapped back on the images and visually compared to the raw data in the MCD viewer. Finally, cluster abundances per image were combined and normalized to cells per square millimeter. Expression of HLA-G was assessed by counting the number of cells in each phenotype with a marker expression above 0.2. Data were combined in R-studio (R version 4.2.0) using the ggplot2 (version 3.4.0) and ComplexHeatmap (version 2.14.0) packages.

### 2.7 VA intraepithelial mononuclear cell isolation

In the operating room, freshly resected healthy VA were collected, after which fecal content and mesoappendiceal fat were immediately removed. The VA were transported to the lab in RPMI media (Gibco: Paisley, Scotland. ThermoFisher Scientific: Waltham, Massachusetts, USA) supplemented with 100 µg/mL Primocin antimicrobial agent (ThermoFisher Scientific, NC9141851). In a laminal flow cabinet, mononuclear cells were isolated as follows: the VA were cut into 0.3 cm pieces before being vortexed and washed in sterile DPBS without calcium and magnesium (Sigma, D8537, St Louis, Missouri, USA) multiple times until the solution was clear and without visible debris. The tissue was incubated for 20 min at 37°C in DPBS containing 10 mM HEPES (ThermoFisher Scientific, 11560496); 5 mM EDTA (Sigma Aldrich, 03690); 1 mM DTT (VWR A3668.0050), and 5% heat-inactivated fetal bovine serum (Gibco, 10270106, endotoxin 0.400 EU/mL) in a shaking water bath. The sample was vortexed rigorously before being run through a 100-µm cell strainer (Falcon Corning, 352360, Corning, NY, USA). This incubation step of the tissue was repeated, after which the tissue was incubated in DPBS 5% FBS for 10 min at 37°C. All the supernatant was collected and centrifuged at 500×*g* at 20°C for 5 min. Cell pellets were resuspended in 30% Percoll solution (Sigma Aldrich, P1644) and carefully overlayed on 60% Percoll solution, after which the tubes were centrifuged at 1,000×*g* at 20°C for 20 min without brakes. The cells collected at the interface of the two Percoll solutions were collected and considered mononuclear cells. Mononuclear cells were washed in RPMI after being centrifuged at 500×*g* for 5 min at 20°C. Mononuclear cells were counted using Trypan Blue (Invitrogen, T10282) on the Countess II (Invitrogen, Carlsbad, CA, USA) and cryopreserved in 90% FBS and 10% DMSO (Sigma Aldrich, D2650) and stored in liquid nitrogen until flow cytometry analyses.

### 2.8 Flow cytometry analysis

The antibodies that were used are specified in **Supplementary Table S2**. Flow cytometry was performed on cryopreserved mononuclear cells, and all stainings were performed at 4°C. First, cell surface staining was performed for 30 min, followed by incubation with Fixable Viability Dye (Aqua L/D, eBioscience, ThermoFisher, Waltham, MA, USA) for 15 min. Subsequently, cells were fixed and permeabilized (FoxP3 Transcription Factor Staining Buffer Set, eBioscience) and stained intracellularly for Ki67 for 60 min. Antibody binding was analyzed on a FACSymphony A5 (BD Biosciences, Franklin Lakes, NJ, USA) using BD FACSDiva software (BD Biosciences). Data were analyzed using FlowJo software (Tree Star, Ashland, OR, USA). The gating strategy can be found in **Supplementary Figure S4A**. Data from samples with mononuclear cell type counts <100 were discarded from the analyses, as well as samples with B cell subset counts <25. In the bar graphs showing the percentages of surface activation marker positive cells, values <0.10% were visualized as 0.10% in the bar graphs with a log scale for the *y*-axis, whereas statistical analyses were performed on the true percentages.

### 2.9 VA Fecal microbiome sequencing

In the operating room, the resected VA were immediately opened longitudinally, and fecal content was collected in stool container cups (DAKL727) and snap-frozen on dry ice instantly. Samples were stored at −80°C and shipped to CosmosID, Germantown, MD, USA. DNA from stool samples was extracted using the PowerSoil Pro kit according to the manufacturer’s protocol. Extracted DNA samples were quantified with the GloMax Plate Reader System (Promega, Madison, WI, USA) using QuantiFluor^®^ dsDNA System (Promega) chemistry. DNA libraries were prepared using the Nextera XT DNA Library Preparation Kit (Illumina, San Diego, CA, USA) and IDT Unique Dual Indexes with total DNA input of 1 ng. Genomic DNA was fragmented using a proportional amount of Illumina Nextera XT fragmentation enzyme. Unique dual indexes were added to each sample, followed by 12 cycles of PCR to construct libraries. DNA libraries were purified using AMpure magnetic Beads (Beckman Coulter, Brea, CA, USA) and eluted in QIAGEN EB buffer. DNA libraries were quantified using the Qubit 4 fluorometer and the Qubit™ dsDNA HS Assay Kit. Libraries were then sequenced on an Illumina HiSeqX platform at 2 × 150 bp. In this primary sequencing run, two samples (APP-003 and c-201) did not meet the throughput requirement, and these libraries were re-sequenced on an Illumina NextSeq2000 platform at 2 × 150 bp.

### 2.10 VA Fecal metagenomics analyses

The CosmosIDHub uses a high-performance data-mining k-mer algorithm that rapidly disambiguates millions of short sequence reads into the discrete genomes, engendering the particular sequences. The pipeline has two separable comparators: the first consists of a precomputation phase for reference databases, and the second is a per-sample computation. The input to the precomputation phase are databases of reference genomes and virulence markers that CosmosID scientists continuously curate. The output of the pre-computational phase is a phylogeny tree of microbes, together with sets of variable-length k-mer fingerprints (biomarkers) uniquely associated with distinct branches and leaves of the tree. The second per-sample computational phase searches the hundreds of millions of short sequence reads, or alternatively contigs from draft *de novo* assemblies, against the fingerprint sets. This query enables the sensitive and precise detection and taxonomic classification of microbial NGS reads. The resulting statistics are analyzed to return the fine-grain taxonomic and relative abundance estimates for the microbial NGS datasets. To exclude false-positive identifications, the results are filtered using a filtering threshold derived based on internal statistical scores that are determined by analyzing a large number of diverse metagenomes. The same approach is applied to enable the sensitive and accurate detection of genetic markers for virulence factors. The number of taxonomically significant bacterial hits in the Bacteria 2.0.1 database that were found in the samples and used to make identifications was listed as a number of hits that have met the threshold for high confidence that the organism called.

The alpha diversity abundance score was used as input. The abundance score is a normalized abundance metric that reflects the underlying microbiome composition of the community. The aggregation level of the input data for comparative analysis has been set to the species level. The CHAO1 index—appropriate for abundance data—assumes that the number of organisms identified for a taxa has a Poisson distribution and corrects for variance. It is helpful for data sets skewed toward low-abundance calls, as is often the case with microbes. The Simpson diversity index is used to calculate a measure of diversity, considering the number and abundance of taxa. The Simpson index gives more weight to common or dominant species, which means a few rare species with only a few representatives will not affect the diversity of the sample. The Shannon index summarizes the diversity in the population while assuming all species are represented in a sample and that they are randomly sampled. The Shannon index increases as both the richness and evenness of the community increase.

Beta diversity is a comparison of samples to each other and answers how different they are. It measures the distance or dissimilarity between each sample pair. A beta diversity distance matrix, where the input metric is relative abundance, reflects the underlying microbiome composition of the community. The aggregation level of the input data has been set to species level. PERMANOVA stands for permutational multivariate analysis of variance and is a nonparametric multivariate statistical test. The test is based on the prior calculation of the distance between any two cohorts included in the principal coordinate analysis of beta diversity. PERMANOVA measures the sum-of-squares within and between cohorts and makes use of the *F*-test to compare within-cohort to between-cohort variance. PERMANOVA draws tests for significance by comparing the actual *F*-test result to that gained from random permutations of the objects between the groups.

#### 2.10.1 LDA analysis

Linear discriminant analysis effect size (LEfSe) is an algorithm for high-dimensional biomarker discovery that identifies genomic features (genes, pathways, or taxa) characterizing the differences between two or more biological conditions. Specifically, the nonparametric factorial Kruskal–Wallis (KW) sum-rank test is used to detect features with significant differential abundance with respect to the class of interest. As a last step, LEfSe uses linear discriminant analysis to estimate the effect size of each differentially abundant feature and rank the feature accordingly.

### 2.11 Quantitation and statistical analysis

When comparing continuous data such as patient age and cell scores between two groups, the Mann–Whitney *U* test was used. Dichotomous data between two groups were compared using Fisher’s exact test and between three groups using Chi-square test. Tests were two-sided and *p*-values ≤0.050 were considered statistically significant. The Pearson correlation coefficient calculation between age and gene counts and other statistical testing were performed using GraphPad Prism 9.0.0. Normalized gene counts from the nCounter were evaluated per patient group, and genes with a median count of <100 in any of the groups were discarded. Differential expression (DE) testing between the patient groups was performed using the Advanced Analysis module of nSolver software v2.0 (NanoString, Seattle, WA, USA), and *p*-values were adjusted using the Benjamini–Hochberg (BH) procedure. Only genes that were differentially expressed ≥2-fold, with a BH-adjusted *p*-value of ≤0.010 were considered significant. The DE genes deemed cancer-testis antigens and genes significantly associated with patient age (Pearson *p*-value ≤0.05) were discarded.

### 2.12 Visualization

The schematic study overview figures were created with BioRender.com. Data graphs were made using GraphPad Prism 9.0.0, and the final figures were established using Adobe Illustrator CC2018.

## 3 Results

### 3.1 Transcriptomic VA GALT immune profiles of PDAC patients suggest cytotoxic cell activation and suppressed B cell functions

To investigate the GALT immune profiles in patients with PDAC and colon diseases, we collected non-inflamed, healthy VA (termed VA sana). The patient characteristics of the VA samples included are shown in **Table 1**. Among the three patient groups, there were no differences in age, sex, or cancer stage. Benign colon diseases included ileus, slow transit obstipation, megacolon, diverticulitis, benign mesothelial cysts, and suspicion of appendicitis.

**Table 1:** Patient characteristics of samples included in VA GALT gene expression analysis.

	Benign colon disease (*n* = 9)	Colon AC (*n* = 9)	PDAC (*n* = 16)	*p*-value
Cancer stage				
Local malignancy	N/A	6 (66.7%)	14 (87.5%)	0.312^a^
Metastatic malignancy		3 (33.3%)	2 (12.5%)	
**Age** (years; median (range))	55.0 (43.0–95.0)	59.0 (52.0–76.0)	66.5 (49.0–89.0)	0.194^b^
Sex				
Male	4 (44.4%)	3 (33.3%)	10 (62.5%)	0.348^c^
Female	5 (55.6%)	6 (66.7%)	6 (37.5%)	

VA GALT, vermiform appendix gut-associated lymphoid tissue; n, number; AC, adenocarcinoma; PDAC, pancreatic ductal adenocarcinoma; N/A, not applicable.

^a^Fisher’s exact test.

^b^Welch ANOVA.

^c^Chi-square test.

**Figure 1:**
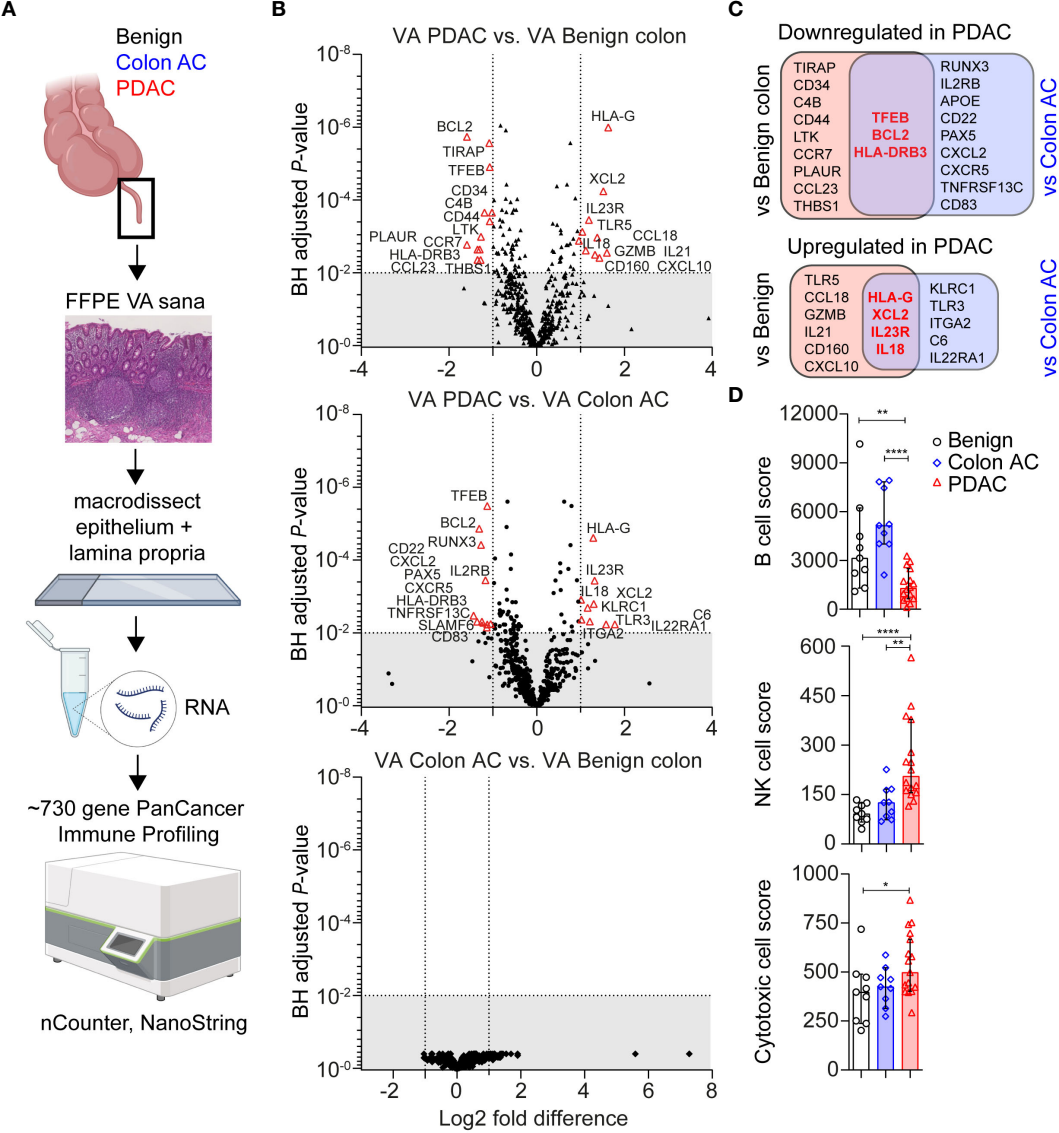
Transcriptomic immune profiles of the vermiform appendix (VA) among three adult patient groups. **(A)** Schematic overview of immune profiling using formalin-fixed paraffin-embedded (FFPE) healthy VA (termed sana) from patients with benign colon disease (*n* = 9); colon adenocarcinoma (AC) (*n* = 9); or pancreatic ductal adenocarcinoma (PDAC) (*n* = 16). The gut-associated lymphoid tissue (GALT) was macrodissected from tissue slides, and immune-related gene expression was quantified using the PanCancer Immune Profiling Panel (NanoString). This figure was created with BioRender.com. **(B)** Volcano plot illustrating differentially expressed (DE) genes in three patient group comparisons (│Fold difference│ ≥ 2, Benjamini–Hochberg-adjusted *p*-value <0.010 cut-off indicated by the gray area). **(C)** Venn diagrams showing the overlap in PDAC-related DE genes compared to benign colon disease or colon AC. Overlapping PDAC-specific genes are in bold and red. **(D)** Median cell type scores in VA in the three patient groups, based on nSolver-assigned immune cell-specific gene expression. Error bars are 95% confidence intervals (CI); *p*-values are calculated using the Mann–Whitney *U* test. ^*^ *p* < 0.050; ^**^ *p* < 0.010; ^****^ *p* < 0.0001.

Immune-related gene expression in the VA GALT was measured using the PanCancer multiplex nucleic acid hybridization panel of NanoString nCounter (**Figure 1A**). Genes with a low average mRNA count <100 and genes that were correlated to age were excluded from the differential expression analysis. In PDAC patients compared to those with benign colon disease, we found that 12 genes were downregulated and 10 genes were upregulated (|Fold difference|>2 and adjusted *p* < 0.010, **Figure 1B**). Compared to colon adenocarcinoma (AC) patients, 12 genes were downregulated and nine genes were upregulated in the PDAC group. No genes were significantly differentially expressed in colon AC versus benign colon disease (**Figure 1B**). The PDAC-specific genes that were identified in both comparisons are indicated in **Figure 1C** and include transcription factor EB (TFEB), BCL2, and HLA-DRB3 as downregulated and HLA-G, XCL2, IL-23R, and IL-18 as upregulated. These genes are indicative of chronic immune stimulation in the VA GALT of PDAC patients. A list of average gene counts per patient group of the complete PanCancer panel is shown in **Supplementary Table S3**.

Based on immune cell-specific gene expression, we found that PDAC VA GALT has a reduced B cell score (median of 1,291) compared to the two other patient groups (median score of 3,141 in benign and 5,148 in colon AC patients, **Figure 1D**). On the other hand, the NK cell score is significantly higher in PDAC VA GALT compared to both groups (median of 206 in PDAC vs. 92 in benign and 126 in colon AC). The cytotoxic cell score is also higher in PDAC VA GALT (median score of 527) compared to benign and colon AC (398, *p* = 0.032 and 242, *p* = 0.108, respectively), only being statistically significant compared to the benign colon group. In summary, the GALT of PDAC patients is distinct from that of patients with colon disease based on gene expression profiling and shows a transcriptional phenotype shifted toward chronic cytotoxic cell stimulation and decreased B cell activity.

### 3.2 The most significantly upregulated gene HLA-G is predominantly expressed in plasma cells

**Figure 2:**
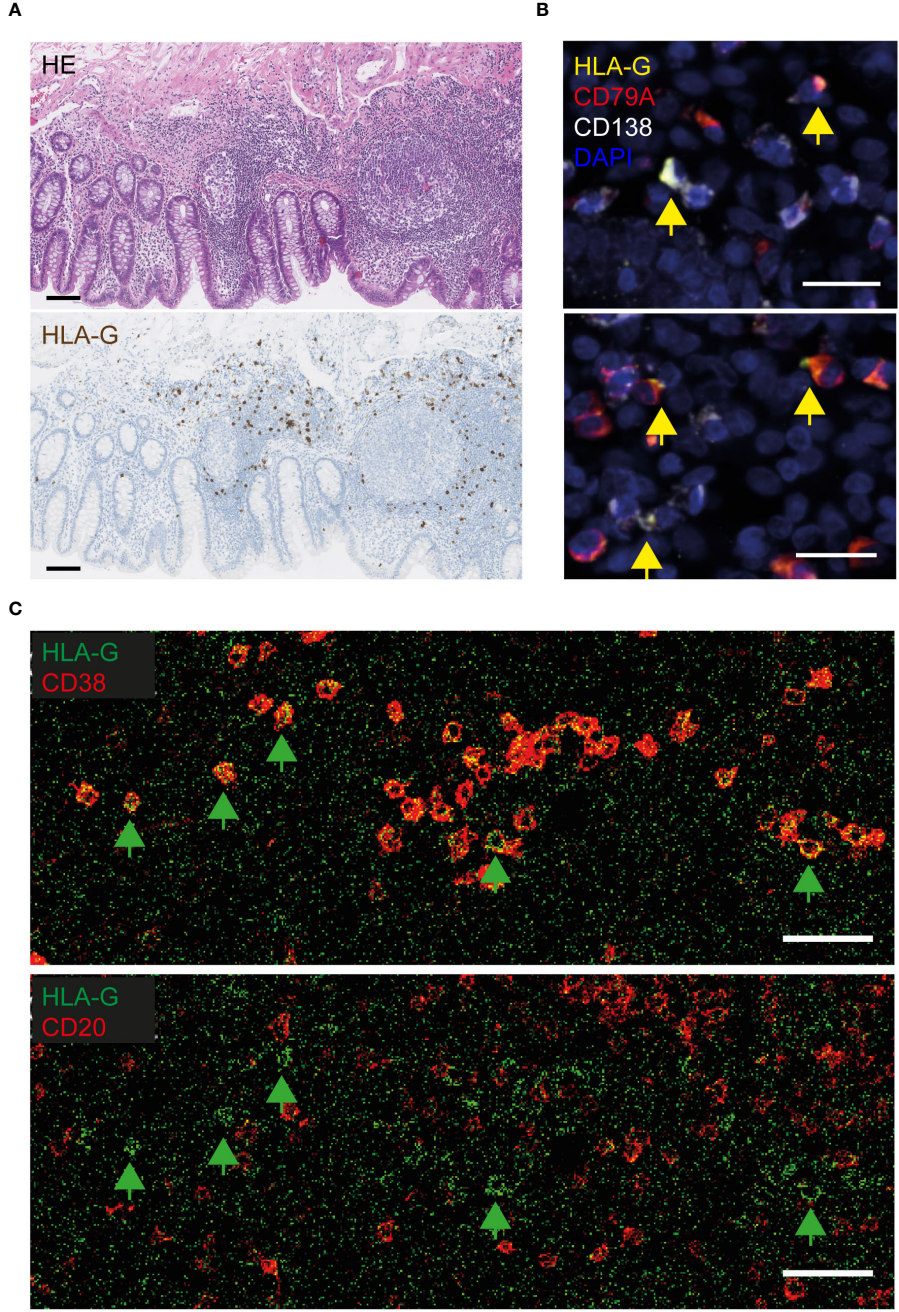
Visualization of HLA-G protein expression in the VA of PDAC patients. **(A)** Representative images of HLA-G protein expression in FFPE VA of a PDAC patient. The upper image of the VA section was stained with hematoxylin and eosin (HE), and the lower section was stained with hematoxylin and an HLA-G antibody visualized with DAB. Scale bars = 100 µm. **(B)** Fluorescent images of VA tissue slides from PDAC patients, stained with antibodies against HLA-G (yellow), CD79A (red, B cell lineage), CD138 (white, plasma cells), and DAPI (navy blue, nuclei). Yellow arrows indicate HLA-G-positive cells. Scale bars = 20 µm. **(C)** Visualization of HLA-G-positive cells in lime green, combined with CD38-expressing (plasma) cells in red (upper image), or combined with CD20^+^ B cells in red (lower image), by imaging mass cytometry. The green arrows indicate surface HLA-G-expressing cells. Scale bars = 50 µm.

Human leukocyte antigen (HLA)-G was the most significantly overexpressed PDAC-specific gene in the VA GALT, based on the NanoString immune profiling. We first performed immunohistochemical protein staining on the tissue sections to investigate which cell types express HLA-G in PDAC VA. Human placenta tissue sections were used as a positive control for HLA-G staining (**Supplementary Figure S1A**). VA tissues from each patient group were stained with an HLA-G antibody to visualize the expression patterns in the GALT (**Supplementary Figure S1B**). The GALT VA contains HLA-G+-expressing cells in each patient group; however, the abundance of HLA-G+ cells is lower in the benign colon patient group (**Supplementary Figure S1C**). A representative image in **Figure 2A** shows that HLA-G is expressed by immune cells in the lamina propria of VA, surrounding the germinal centers. To identify the specific cell types that express HLA-G in PDAC VA GALT, we performed multiplex IF staining for several different cell surface markers, including epithelial, T-cell, B cell, and myeloid cell markers (**Supplementary Figure S2**). We found that HLA-G protein expression predominantly co-localizes with the B cell lineage marker CD79A or with the plasma cell marker CD138 (**Figure 2B**). Next, imaging mass cytometry (IMC) analysis with a panel of 36 markers (**Supplementary Table S1**) was performed on three patient VA tissue slides per disease group. A heatmap of relative VA immune cell abundance in marginal zones is shown in **Supplementary Figure S3A**. The percentage of HLA-G+ cells in the different immune cell types in multiple regions of interest per patient sample (**Supplementary Figure S3B**) revealed that of all cells analyzed in this panel, plasma cells most frequently express HLA-G. Representative images in **Figure 2C** show that HLA-G+-expressing cells are negative for early B cell marker CD20, while all are positive for plasma cell marker CD38, corroborating the IF results and indicating that HLA-G is primarily expressed by plasma cells in the VA GALT.

**Figure 3:**
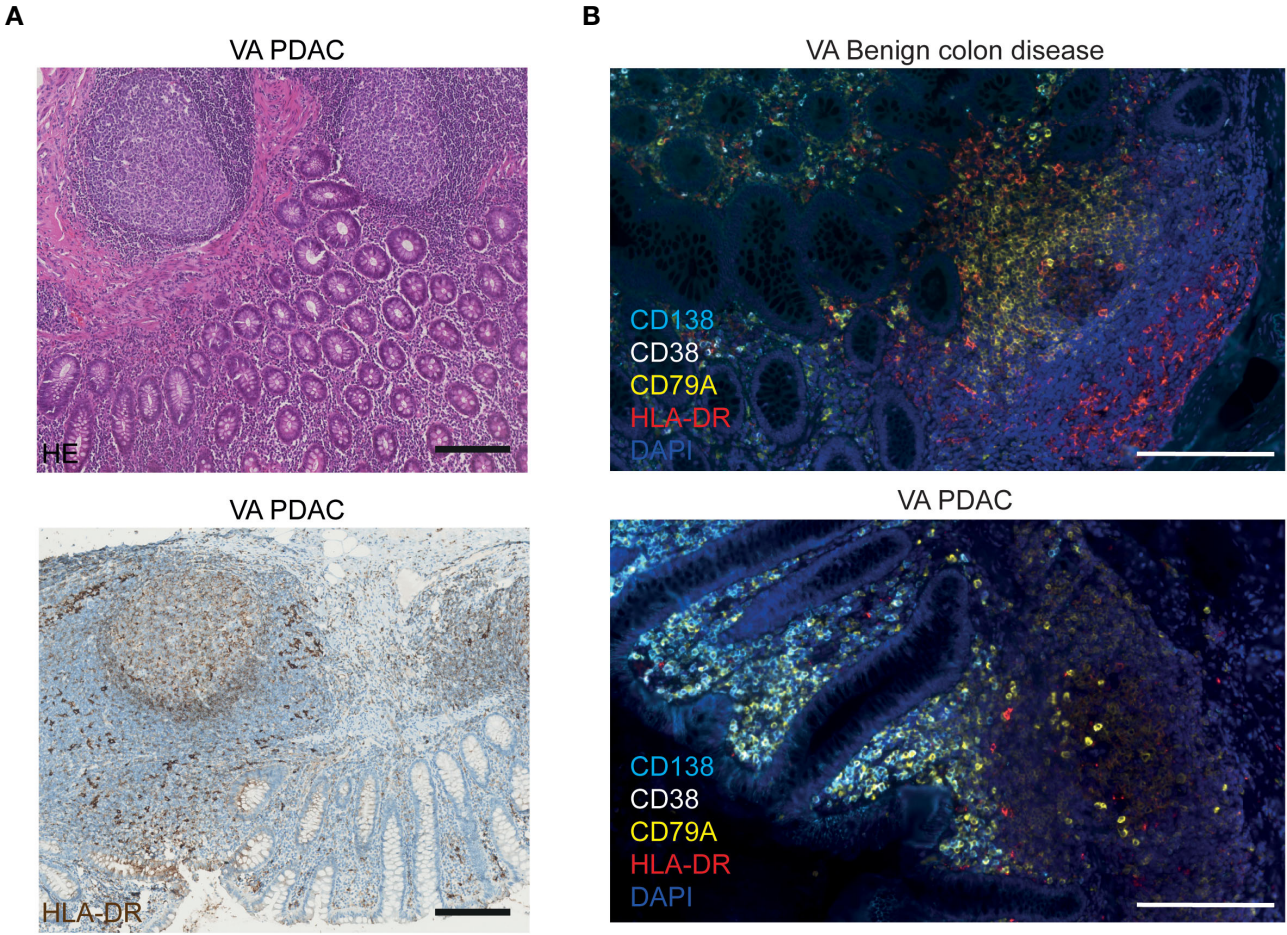
Visualization of HLA-DR protein expression in VA. **(A)** Representative images of FFPE VA tissue sections from a PDAC patient stained with HE (upper image) or with hematoxylin and an HLA-DR antibody visualized with DAB (lower image). Scale bars = 200 µm. **(B)** Fluorescence images of VA tissue slides from a benign colon disease patient (upper image) and from a PDAC patient (lower image), stained with antibodies against HLA-DR (red), CD138 (aqua blue, plasma cells), CD38 (white, plasma cells), CD79A (yellow, all B cells), and DAPI (navy blue, nuclei). Scale bars = 200 µm.

The MHC class II molecule HLA-DR, on the other hand, is known to be expressed by a broad range of antigen-presenting cells (APCs) in lymphoid tissues, including B cells, dendritic cells, macrophages, and activated T cells. As shown above, in **Figure 1C**, we observed that HLA-DRB3 mRNA was downregulated specifically in PDAC VA GALT. **Figure 3A** shows the immunohistochemical protein expression pattern of HLA-DR in PDAC VA GALT. As expected, HLA-DR is abundantly expressed in the GALT by various immune cells in the germinal centers, marginal zones, and submucosa. Only a limited number of HLA-DR-expressing APCs in VA GALT cells are plasma B cells (**Figure 3B**). Cells expressing high levels of HLA-DR are mainly located in the marginal zones of the lymphoid follicles (**Figure 3B**), which seem less prominent in the VA GALT of PDAC patients compared to benign colon disease patients.

### 3.3 PDAC VA GALT contains lower numbers of antigen-presenting B cells

**Figure 4:**
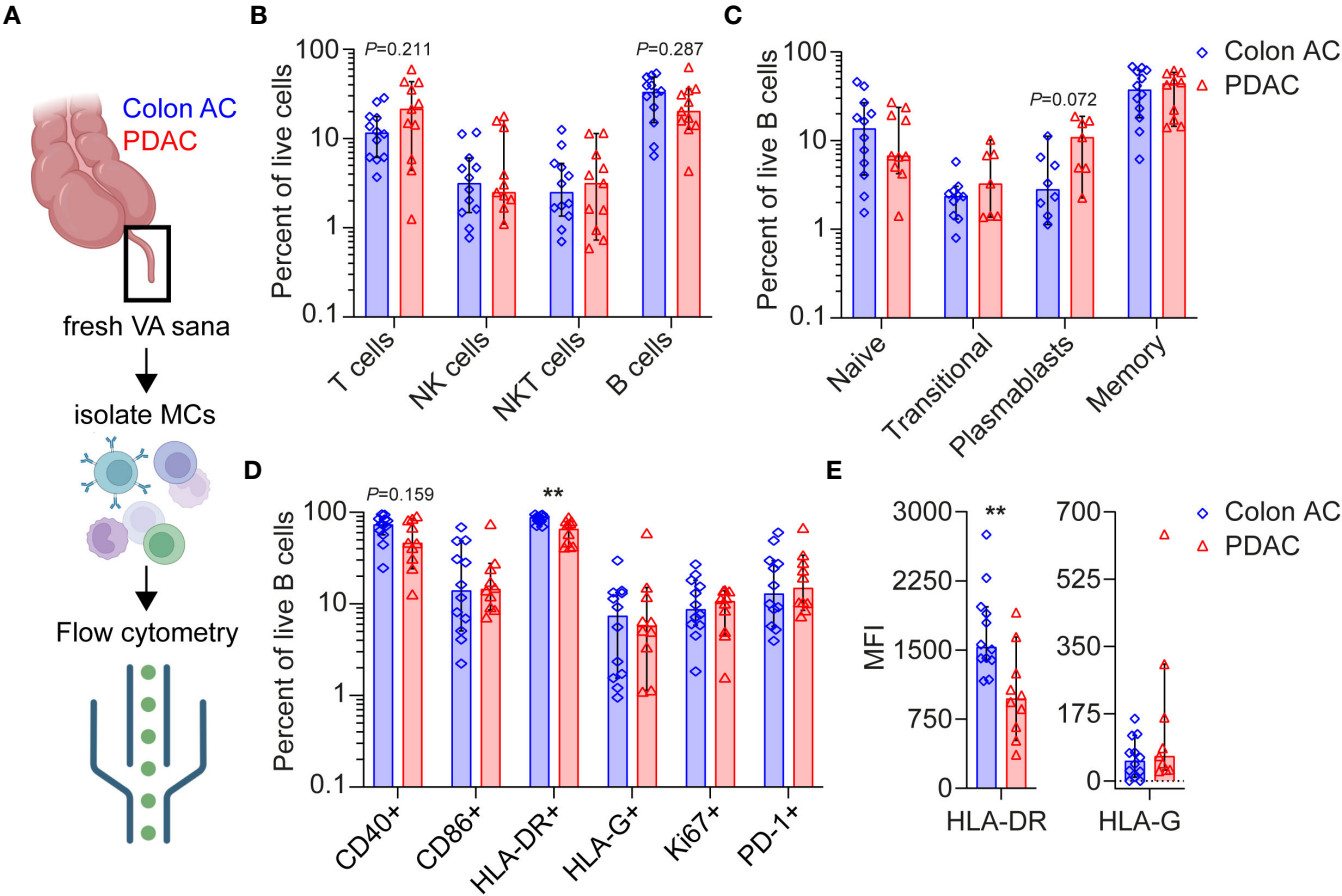
Flow cytometry analyses of VA mononuclear cells from PDAC and colon AC patients. **(A)** Schematic overview of flow cytometry analysis of isolated mononuclear cells (MCs) from freshly resected normal VA (termed VA sana) of (*n* = 12) colon AC patients and (*n* = 11) PDAC patients. This figure was made in BioRender. **(B)** Percentage of live lymphoid cells in the two patient groups. Note the log scale of the *y*-axis. **(C)** Percentage of live B cell types in the two patient groups. Note the log scale of the *y*-axis. **(D)** Percentage of surface marker-positive B cells in the two patient groups. ^**^ *p* < 0.010. Note the log scale of the *y*-axis. **(E)** Median fluorescence intensity (MFI) of HLA-DR and HLA-G expression on B cells. ^**^ *p* < 0.010. AC, adenocarcinoma; PDAC, pancreatic ductal adenocarcinoma. Bars show median percentages; each dot represents an individual patient sample, and error bars are 95% CI; *p*-values are calculated using the Mann–Whitney *U* test.

To further characterize and quantify B cell populations in the VA GALT, we performed flow cytometry analysis (**Figure 4A**). Freshly resected VA from colon AC and PDAC patients were obtained to isolate mononuclear cells. All patients with benign colon disease undergoing right-sided hemicolectomy were treated with systemic immunosuppressive therapy for inflammatory bowel disease and could unfortunately not be included in the flow cytometry analysis to serve as benign controls. The patient characteristics of the samples included are shown in **Table 2**, and the representative flow cytometry gating strategy is shown in **Supplementary Figure S4A**.

**Table 2:** Patient characteristics of samples included in VA mononuclear cell flow cytometry analysis.

	Colon AC (*n* = 12)	PDAC (*n* = 11)	*p*-value
Cancer stage			
Local malignancy	11 (91.7%)	9 (81.8%)	0.590^a^
Metastatic malignancy	1 (8.3%)	2 (18.2%)	
**Age (years), median (range)**	65.0 (57.0–90.0)	71.0 (49.0–75.0)	0.145^b^
Sex			
Male	5 (41.7%)	6 (54.6%)	0.684^a^
Female	7 (58.3%)	5 (45.4%)	
Diabetes			
Yes	2 (16.7%)	6 (54.6%)	0.089^a^
No	10 (83.3%)	5 (45.5%)	

n, number; AC, adenocarcinoma; PDAC, pancreatic ductal adenocarcinoma.

a Fisher’s exact test.

b Mann–Whitney U test.

Overall lymphoid cell numbers were not statistically different in the VA GALT of colon AC vs. PDAC patients (**Figure 4B**). However, the following trends were observed: the median percentages of T cells were 11.6% in colon AC vs. 21.4% in PDAC patients, whereas the median percentages of B cells were 33.1% vs. 20.3%, respectively. The B/T cell ratio was significantly lower in PDAC patients (2.85 in colon AC vs. 0.95 in PDAC patients, *p* = 0.015). We evaluated B cell differentiation and maturation and found that the relative numbers of VA B cell subtypes were similar in the two patient groups (**Figure 4C**). It is noteworthy that the percentage of plasmablasts was trending higher in PDAC patients (median of 10.9% in PDAC vs. 2.8% in colon AC patients, *p* = 0.072). The abundance of IgA-, IgD-, IgG-, and IgM-expressing memory B cells (**Supplementary Figure S5A**) was similar among the two groups. The VA GALT IgD-memory B cells in both patient groups predominantly express IgA (median 53% in colon AC and 58% in PDAC), followed by IgM (median 21% and 19%), and lastly IgG (median 12% and 10%, respectively).

Based on B cell activation marker expression profiles (**Figure 4D**), we found a significantly lower percentage of HLA-DR+ antigen-presenting B cells in the PDAC group (median 88.5% in colon AC vs. 65.5% in PDAC, *p* = 0.003) and a trending decrease in CD40^+^ B cells (73.6% in colon AC vs. 46.3% in PDAC, *p* = 0.159). Surprisingly, the percentage of HLA-G surface-expressing B cells was not different between PDAC and colon cancer patients (**Figure 4D**). Moreover, HLA-G surface expression on NK cell, T-cell, and B cells sets was similar in the two patient groups (**Supplementary Figure S5B**). Of the different VA mononuclear cell types that were analyzed, plasmablasts had the highest percentage of HLA-G-positive cells (median 9%–11% of plasmablasts in colon AC and PDAC patients, **Supplementary Figure S5B**), corroborating the tissue staining. Given the differences in mRNA expression of HLA-DR and HLA-G in PDAC patients (**Figure 1B**), we also evaluated the intensity of protein expression on the VA B cells by flow cytometry analyses (**Figure 4E**). In line with the frequency of HLA-DR- and HLA-G-expressing B cells, we found that expression intensity of HLA-DR is significantly lower on PDAC VA B cells (median MFI 1,532 in colon AC vs. MFI 937 in PDAC, *p* = 0.001), whereas there was no difference in surface expression intensity of HLA-G on B cells between the two patient groups. We further analyzed the activation maker expression in the different B cell subsets (**Supplementary Figure S5C**). On naïve and transitional B cells, there were no differences in activation marker expression between the two patient groups. On the more differentiated B cells, however, we found that the percentage of HLA-DR+ memory B cells was significantly reduced in PDAC patients (median 94.5% in colon AC vs. 76.7% in PDAC, *p* = 0.023), as shown in **Supplementary Figure S5C**.

To summarize our findings, the VA GALT of PDAC patients is relatively rich in T cells and poor in B cells compared to colon cancer patients. Plasmablasts are abundant, whereas antigen-presenting memory B cells are significantly reduced in the VA of PDAC patients, as observed by lower frequencies of MHC-II and CD40-expressing memory B cells. HLA-G is highly overexpressed at the mRNA level in VA of PDAC patients, although the HLA-G surface protein expression on B cells is not increased, suggesting either any translation or secretion of HLA-G protein. Overall, the GALT immune signature of PDAC patients shows signs of chronic GALT immune stimulation, and our findings could indicate that VA immune cell cytotoxicity aid by B cells is impaired in these patients.

### 3.4 Comparative metagenomics of VA microbiome reveals dysbiosis in PDAC patients

**Figure 5:**
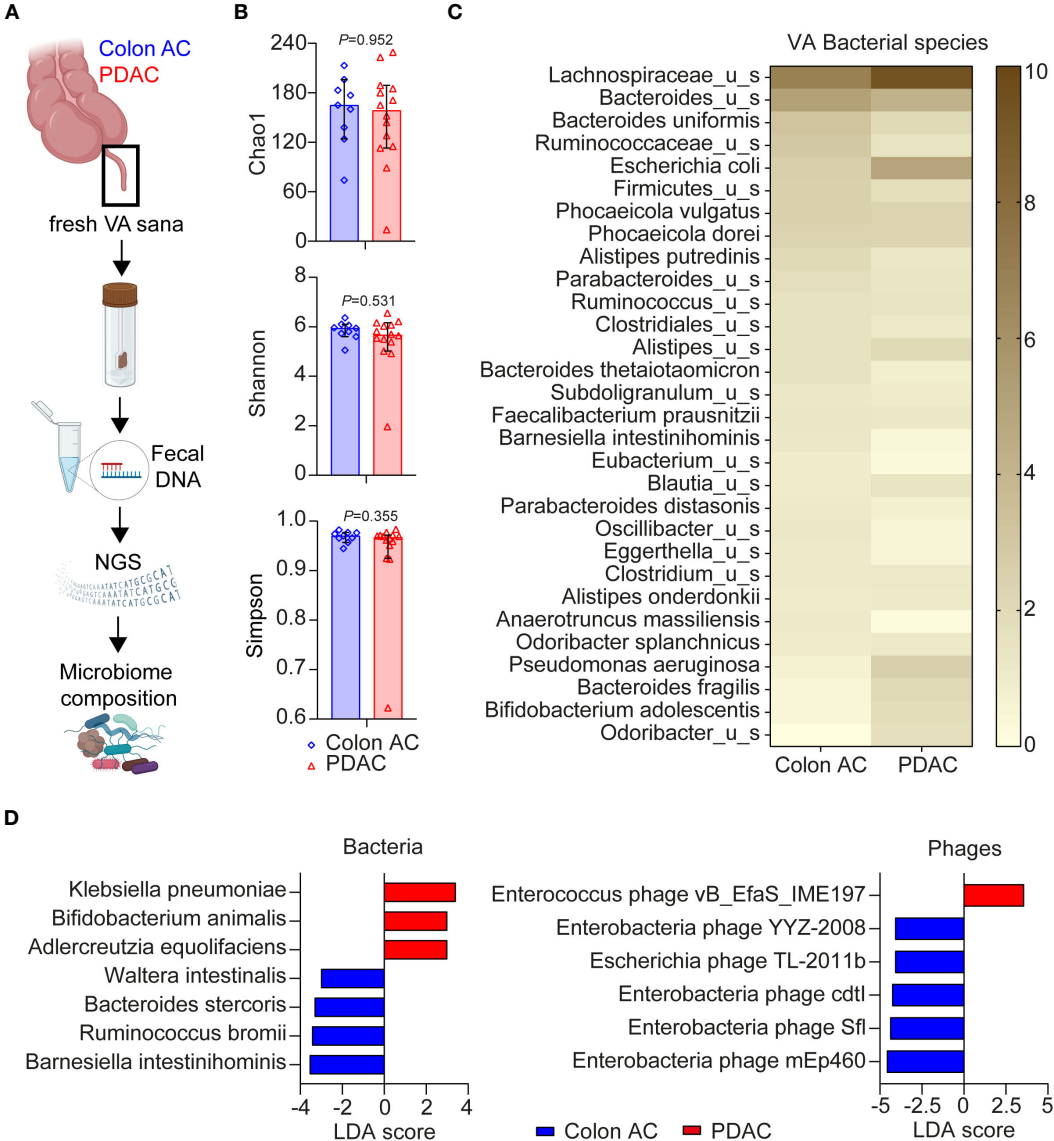
Microbiome composition in VA of PDAC and colon AC patients. **(A)** Schematic overview of VA microbiome analysis. AC, adenocarcinoma; PDAC, pancreatic ductal adenocarcinoma; VA, vermiform appendix; sana, healthy; NGS, next-generation sequencing. This figure was made in BioRender. **(B)** Alpha diversity scores of bacterial species of the two patient groups are represented as median with 95% CI; each dot represents an individual patient sample; *p*-values are calculated using the Wilcoxon rank sum test. *N* = 9 colon AC patients, *n* = 15 PDAC patients. **(C)** Heatmap of relative bacterial species abundance, cut-off ≥1% average abundance in at least one patient group. Color gradient: 0%–10% average relative abundance. **(D)** Linear discriminant analysis effect size (LEfSe) analysis of bacteria and phages in the two patient groups. Cut-off linear discriminant analysis (LDA) score ≥3 and *p* < 0.050.

Given the close interaction between the gut microbiome and mucosal immune system, we compared VA microbiome compositions in colon AC (*n* = 13) and PDAC (*n* = 14) patient samples by metagenomic analyses (**Figure 5A**). The patient characteristics were similar between the two groups, as shown in **Table 3**. The number of sequencing reads and bacterial hits among the two patient groups was similar (**Supplementary Figures S6A, B**). Four out of 13 colon AC samples resulted in less than 50 bacterial hits (**Supplementary Figure S6B**), and these samples were therefore excluded from further analyses. There was no difference in microbial alpha diversity (**Figure 5B**) nor in beta diversity: Jaccard PERMANOVA *p* = 0.921; Bray–Curtis beta PERMANOVA *p* = 0.881, data not shown. Moreover, the relative bacterial class abundance was similar in colon AC and PDAC patients (**Supplementary Figure S6C**, *p* = 0.998). The most abundant bacterial species that reached an average abundance of ≥1% in either patient group is shown in **Figure 5C**. The two most abundant bacteria in the VA were Lachnospiraceae and Bacteroides. Differential abundance evaluation of bacterial species by linear discriminant analysis revealed three species enriched and four species depleted in the PDAC VA (**Figure 5D**). The most enriched species in the PDAC group is the opportunistic *Klebsiella pneumoniae*, whereas the most depleted species are the commensals *Barnesiella intestinihominis* and *Ruminococcus bromii*.

**Table 3:** Patient characteristics of samples included in VA microbiome analysis.

	Colon AC (*n* = 13^a^)	PDAC (*n* = 14)	*p*-value
Cancer stage			
Local malignancy	13 (92.9%)	12 (85.7%)	>0.999^b^
Metastatic malignancy	1 (7.1%)	2 (14.3%)	
**Age (years), median (range)**	69.0 (57.0–90.0)	64.5 (49.9–89.0)	0.394^c^
Sex			
Male	4 (30.8%)	9 (64.3%)	0.128^b^
Female	9 (69.2%)	5 (35.7%)	
Diabetes			
Yes	3 (23.1%)	7 (50.0%	0.237^b^
No	10 (76.9%)	7 (50.0%	

n, number. AC, adenocarcinoma; PDAC, pancreatic ductal adenocarcinoma.

a Four out of 13 patients were excluded from the analysis due to low bacterial hits.

b Fisher’s exact test.

c Mann–Whitney U test.

Moreover, multiple virulence factors are differentially abundant among the patient groups, mostly originating from *Bacteroides fragilis* (**Supplementary Figure S6D**). Of the highly abundant virulence factors in the PDAC group, some originate from *Escherichia coli*, *Klebsiella*, or *Shigella flexneri*. Additional LEfSe analysis revealed that one *Enterococcus* phage is more abundant in PDAC patients, whereas five phages were less abundant (**Figure 5D**), of which four are enterobacteria-targeting phages. Interestingly, the PDAC GALT-enriched *Klebsiella pneumoniae* belongs to the enterobacteria. To summarize the metatranscriptome analyses, we found microbial dysbiosis in the VA of PDAC patients dominated by *Klebsiella pneumoniae*, *Bifidobacterium animalis*, and *Adlercreutzia equolifaciens*, which may play a significant role in the immune landscape of PDAC patients. An overview of all individual VA patient samples included in the three various analyses of this study is shown in **Supplementary Table S4**.

## 4 Discussion

This is the first study describing the normal appendix GALT immune profiles in PDAC and colon AC patients. Although immune profiles of pancreatic and colon tumors are extensively investigated by comparing normal pancreas and colon tissues, no studies have reported gene expression data of normal-looking VA GALT in these patient groups until now. Although we found no significant VA GALT gene expression differences between benign and malignant colon disease patients using a targeted immune profiling panel (**Figure 1B**), there are no reference studies available with normal VA data from age-matched patients to compare our results with. A validation study with a larger patient cohort is necessary to further investigate this finding.

We found that transcriptomic NK and cytotoxic cell signatures were high in PDAC patients. In particular, HLA-G, XCL2, IL-23R, and IL-18 were overexpressed in PDAC GALT. The immune checkpoint molecule HLA-G induces immune escape by decreasing MHC-II presentation pathways and inhibiting cytolysis of effector cells (18). HLA-G is an HLA-class Ib molecule and is known to be overexpressed in the placenta for fetal-maternal tolerance, in cancer tissues, and in inflammatory and viral diseases (18). We found that HLA-G in the GALT is predominantly expressed by plasma cells. The fact that the HLA-G mRNA overexpression did not result in higher surface protein expression on B cells may indicate that HLA-G is secreted. The NanoString probe used to quantify HLA-G mRNA levels covers the 3′UTR (exons 7 and 8). This gene region is an important regulatory region influencing HLA-G mRNA stability, turnover, mobility, and splicing patterns. Different 3′UTR haplotypes and polymorphisms are associated with differential soluble HLA-G expression (19). It is known that soluble HLA-G is increased in the circulation of PDAC patients (20). Further in-depth analysis is needed to elucidate differential isoform expression and the functions of HLA-G secretion in the VA GALT.

Chemokine XCL2 is an inducer of M1 macrophage chemotaxis (21), and most importantly, it is overexpressed by antigen-responsive CD8^+^ T cells (22, 23). It is shown that XCL2 is upregulated in various human cancers and correlated with activated T-cell immune signatures in renal cancer (24). IL-23R, on the other hand, is known to be highly expressed on regulatory T cells (Tregs). Interestingly, IL-23R expressed by tumor-infiltrating Treg promotes suppressive activity (25), whereas IL-23R signaling on Treg in the gut impairs suppressive function and results in Treg apoptosis (26). The proinflammatory cytokine IL-18 is another PDAC-specific overexpressed gene in the VA GALT. Macrophages normally produce IL-18, which activates intratumoral T cells and induces IFN-y expression (27).

Lastly, the most downregulated gene in the PDAC GALT was the pro-survival gene BCL2, suggestive of a shift in the balance between naïve T cells and activated T cells. Naive T cells express high levels of BCL2 and are heavily dependent on BCL2 for survival, whereas activated T cells alter their survival program and are less reliant on BCL2 (28). Our transcriptomic immune profiling data implies that the VA of PDAC patients may be a potential source of cytotoxic cells, which should be explored in future functional studies. Future studies are needed to assess the functionality of the various VA mononuclear cell populations by ELISpot and/or flow cytometry analysis of effector- and immunosuppressive cytokine production. Our findings have stimulated our interest in performing future studies to evaluate the cytotoxic cell capacity of VA GALT-derived mononuclear cells against PDAC antigens, patient PDAC-derived organoids, as well as PDAC microbial-related antigens, in the presence or absence of HLA-G blockade.

In contrast to the activated cytotoxic cell phenotype, the B cells in the VA GALT of PDAC patients showed diminished activity, specifically by the NanoString B cell score, the high HLA-G mRNA expression, and reduced levels of the antigen presentation molecule HLA-DR. Increasing evidence highlights the importance of B cells in PDAC immune responses, both locally and systemically. Immune cell functions of B cells include the production of cytokines and neutralizing antibodies, interaction with the complement cascade, antigen presentation, and direct communication with effector immune cells (29). Additionally, B cells can exert immune regulation (30) and induce PDAC tissue fibrosis (31). In PDAC tumors, B cells as well as immunoglobulins are highly abundant (32–34), and lower stromal B cell tumor infiltration is related to a worse prognosis (35, 36). On the other hand, the level of circulating B cells in the peripheral blood of PDAC patients is lower compared to healthy controls (1).

We found that TFEB was specifically downregulated in PDAC VA GALT. TFEB functions as a master regulator of lysosomal biogenesis (37), critical for the degradation of peptides and nucleic acids. Its downregulation likely disrupts the antigen-presenting roles of lysosomes, leading to an impaired adaptive immune system and antigen presentation (38). Moreover, TFEB plays an essential role in humoral activity by promoting immunoglobulin production and CD40 ligand expression by CD4^+^ T cells (39). CD40, a TNF superfamily receptor, is expressed on multiple immune cells. Others have shown that CD40 activation on B cells can promote antitumor effects by providing support to already existing T-cell immunity or by functioning as potent APCs and generating effector T-cell activity (40). The downregulation of TFEB and the trending decrease in CD40^+^ B cells may imply once more that in the PDAC VA GALT, immune cell cytotoxicity aid by B cells is impaired.

An additional interesting finding in our study is the trending increase of plasmablasts in the PDAC VA GALT. Plasmablasts find themselves between the stage of activated B cells and plasma cells, are rare in the blood of healthy individuals, and are expanded briefly during infection (41). In patients with autoimmunity or conditions of chronic antigen exposure, however, plasmablasts circulate for longer periods, as described in patients with inflammatory bowel disease, rheumatoid arthritis, systemic lupus erythematosus, and even patients with PDAC (31). This may suggest that chronic antigenic stimulation in PDAC patients occurs on a systemic level, including in the appendix GALT, confirming the notion that PDAC is essentially a systemic disease.

Finally, we observed that in the VA, considered a “safe-house” harboring the gut microbiome fingerprint of individuals, the abundance of certain bacteria, virulence factors, and phages are altered in PDAC patients. Most importantly, *Klebsiella pneumoniae* is enriched, and its targeting of Enterobacteria phages is reduced in PDAC patients. *Klebsiella pneumoniae* is notorious for its ability to cause liver abscesses and lipopolysaccharide (LPS) production. Others have shown that the presence of *Klebsiella pneumoniae* in PDAC tumors is associated with increased tumor size, gemcitabine resistance, and decreased overall survival in resected PDAC patients (42). In addition, long-term exposure to LPS dramatically decreases TFEB expression by macrophages (43), which is in line with our observation of decreased TFEB expression in the PDAC VA GALT.

Another enriched bacteria in the PDAC VA GALT, *Adlercreutzia equolifaciens*, produces equol out of isoflavones (44). Isoflavones have been associated with health benefits and decreased risk of cancer and can reduce PDAC proliferation and migration (45). In melanoma patients resistant to immune checkpoint therapy, *Adlercreutzia equolifaciens* was enriched in the gut microbiome, as shown by Limeta et al. (46). Another observation in our study was the relative abundance of *Pseudomonas aeruginosa* in five of 14 PDAC patients. This opportunistic pathogen is notorious for infecting immunodeficient tissues. Not only can *P. aeruginosa* damage epithelial cells with the various virulence factors it produces, but this pathogen can also induce Toll-like receptors to activate cytokines, gut barrier disruption, and inflammation (47).

The depleted microbes in the PDAC VA GALT were the commensals *Barnesiella intestinihominis* and *Ruminococcus bromii*, among others. Interestingly, these are both well-known commensals of the human gut, and Limeta et al. also report that *Barnesiella intestinihominis* and *Ruminococcus* species are enriched in melanoma patients responding to immunotherapy. In patients with metastatic renal cancer, *Barnesiella intestinihominis* was almost exclusively present in the stool of patients who achieved clinical benefit from VEGF kinase inhibitor therapy (48), and in patients with ovarian and lung cancer receiving chemoimmunotherapy, *B. intestinihominis* within the gut was associated with longer survival (49). Our findings suggest that the VA GALT is a potential source of microbial targets for PDAC therapy.

This study has a number of limitations. First of all, the number of patients in this exploratory study is limited. Obtaining fresh samples with sufficient appendix fecal content and sufficient numbers of live mononuclear cells for experimental purposes from patients meeting all the exclusion and inclusion criteria was challenging. The lifestyle, geography, genetic backgrounds, and diet of our patient cohort are relatively homogeneous. Nonsignificant outcomes for certain experiments could have been due to a limited patient cohort, and validation of our observations in independent cohorts is necessary to confirm our conclusions. Statistical methods in this article were designed to extract features distinct per patient group, and data from flow cytometry and microbiome analyses were not adjusted for multiple testing. Survival analyses using our observed PDAC-specific markers would be an evident next step, applied to a larger patient cohort. Archival formalin-fixed paraffin embedded material of healthy VA from patients with benign colon diseases was easily obtained, as opposed to freshly resected VA. Unfortunately, we were not able to obtain fresh mononuclear cells and VA fecal material from age-matched patients with benign disease undergoing ileo-caecal resection who were not treated with systemic immunosuppressive drugs. Therefore, no flow cytometry or microbiome analysis could be performed in a benign colon disease control group.

Our findings imply a complex interplay between the gut microbiome, immune response, and pancreatic cancer. Diminished B cell function in the GALT may be a crucial component in the immune escape of PDAC and could provide a therapeutic target in PDAC patients. Our findings argue that GALT B cell dysregulation in PDAC patients may be related to microbial dysbiosis, which could potentially be targeted to synergize with cytotoxic cell-directed immunotherapy. Further research is needed to confirm these associations and elucidate potential therapeutic targets.

## Data availability statement

The datasets presented in this study can be found in online repositories. The names of the repository/repositories and accession number(s) can be found below: NCBI database, under accessions GSE236951 and PRJNA996050.

## Ethics statement

The studies involving humans were approved by the Medical Ethical Committee of the Erasmus Medical Center Rotterdam. The studies were conducted in accordance with the local legislation and institutional requirements. The participants provided their written informed consent to participate in this study.

## Author contributions

EV and CE contributed to the conception, design, and project administration of the study. DL, MV, EO, RW, RH, AB, BK, PD, VV, AO, FM, MW, JA, RK, NM, TB, YM, and PK included patients, collected samples, and assisted in resources, methods, and investigation. EV, MV, MW, RK, and TB performed statistical analyses, and CE acquired funding and supervised the study. EV wrote the first draft of the manuscript. All authors contributed to the manuscript revision and read and approved the submitted version.

## Supplementary material

Data Sheet 1 Word file = Supplementary Tables 1-4 and Supplementary figure legends

Image 1 = Supplementary Figure S1

Image 2 = Supplementary Figure S2

Image 3 = Supplementary Figure S3

Image 4 = Supplementary Figure S4

Image 5 = Supplementary Figure S5

Image 6 = Supplementary Figure S6
