# Supplementary material for: B cell immune profiles in dysbiotic vermiform appendixes of pancreatic cancer patients
Source: Front Immunol. 2023 Nov 10;14:1230306. doi: 10.3389/fimmu.2023.1230306 (PMC10667699; doi:10.3389/fimmu.2023.1230306)
Supplement: Supplementary file 1 [file DataSheet_1.docx]

Supplementary Material

B cell immune profiles in dysbiotic vermiform appendixes of pancreatic cancer patients

Eveline E. Vietsch^1,2^, Diba Latifi^2^, Maaike Verheij^2^, Elise W.A. van der Oost^2^, Roeland F. de Wilde^2^, Roel Haen^2^, Anne Loes van den Boom^2,3^, Bas Groot Koerkamp^2^, Pascal G. Doornebosch^4^, Victorien M.T. van Verschuer^4^, Ariadne H.A.G. Ooms^5,7^, Farzana Mohammad^1^, Marcella Willemsen^1^, Joachim G.J.V. Aerts^1^, Ricki T. Krog^6^, Noel F.C.C. de Miranda^6^, Thierry P.P. van den Bosch^7^, Yvonne M. Mueller^8^, Peter D. Katsikis^8^ and Casper H.J. van Eijck^2,*^

^1^ Department of Pulmonary Medicine, Erasmus MC Cancer Institute, Rotterdam, The Netherlands

^2^ Department of Surgery, Erasmus MC Cancer Institute, Rotterdam, The Netherlands

^3^ Department of Surgery, Reinier de Graaf Hospital, Delft, The Netherlands.

^4^ Department of Surgery, IJsselland Hospital, Capelle aan den IJssel, the Netherlands

^5^ Department of Pathology, Pathan BV, Rotterdam, the Netherlands

^6^ Department of Pathology, Leiden University Medical Center, Leiden, Netherlands

^7^ Department of Pathology, Erasmus University Medical Center, Rotterdam, Netherlands

^8^ Department of Immunology, Erasmus University Medical Center, Rotterdam, the Netherlands

*** Correspondence:**
Prof. dr. Casper H.J. van Eijck, MD, PhD, Department of Surgery, Erasmus University Medical Center, P.O. box 2040, Rotterdam, 3000 CA, The Netherlands, c.vaneijck@erasmusmc.nl

# Supplementary Tables

**Supplementary Table 1.** Imaging Mass Cytometry Immune-cell Panel.

| **Antibody and metal** | | | | **Incubation** | | |
| --- | --- | --- | --- | --- | --- | --- |
| **Target** | **Clone** | **Manufacturer** | **Metal** | **Time** | **Temp** | **Dilution** |
| **HLA-DR** | TAL 1B5 | ThermoFisher | 141 Pr | 5h | 20 ℃ | 100 |
| **CD20** | H1 | BD Bioscience | 142 Nd | O/N | 4℃ | 100 |
| **CD68** | D4B9C | CST | 143 Nd | O/N | 4℃ | 100 |
| **CD4 + 2nd Ab** | EPR6855 | Abcam | 145 Nd | Indirect O/N | 4℃ | 50 |
| **CD8a** | D8A8Y | CST | 146 Nd | 5h | 20 ℃ | 50 |
| **CD31** | 89C2 | CST | 147 Sm | O/N | 4℃ | 100 |
| **DC-LAMP+** | 1010E1.01 | Novus Bio | 148 Nd | O/N | 4℃ | 50 |
| **CD45** | D9M8I | CST | 149 Sm | O/N | 4℃ | 50 |
| **IgG4** | EP4420 | Abcam | 151 Eu | 5h | 20 ℃ | 100 |
| **Ki-67** | 8D5 | CST | 152 Sm | O/N | 4℃ | 100 |
| **CD3** | EP449E | Abcam | 153 Eu | O/N | 4℃ | 50 |
| **IgG1** | EPR4417 | Abcam | 154 Sm | O/N | 4℃ | 100 |
| **CD138** | MI15 | BioLegend | 155 Gd | 5h | 20 ℃ | 25 |
| **TIGIT** | BLR047F | Abcam | 156 Gd | 5h | 20 ℃ | 50 |
| **CD39** | EPR20627 | Abcam | 157 Gd | 5h | 20 ℃ | 100 |
| **PNAd** | MECA-79 | BioLegend | 158 Gd | 5h | 20 ℃ | 100 |
| **FOXP3** | D608R | CST | 159 Tb | O/N | 4℃ | 50 |
| **PD-1** | D4W2J | CST | 160 Gd | 5h | 20 ℃ | 50 |
| **ICOS** | D1K2T(tm) | CST | 161 Dy | 5h | 20 ℃ | 50 |
| **HLA-G + 2nd Ab** | MEM-G/2 | GeneTex | 162 Dy | Indirect O/N | 4℃ | 50 |
| **CD14** | D7A2T | CST | 163 Dy | 5h | 20 ℃ | 100 |
| **AID** | EPR23436-45 | Abcam | 164 Dy | O/N | 4℃ | 25 |
| **CD45RO** | UCHL1 | CST | 165 Ho | O/N | 4℃ | 100 |
| **BCL-6** | E5I8I | CST | 166 Er | O/N | 4℃ | 100 |
| **TCF7** | C63D9 | CST | 168 Er | 5h | 20 ℃ | 100 |
| **CD38** | EPR4106 | Abcam | 169 Tm | O/N | 4℃ | 100 |
| **Tbet** | 4B10 | Abcam | 170 Er | 5h | 20 ℃ | 50 |
| **CD21** | EP3093 | Abcam | 171 Yb | 5h | 20 ℃ | 100 |
| **CD19** | D4V4B | CST | 172 Yb | 5h | 20 ℃ | 100 |
| **CD163** | EPR14643-36 | Abcam | 173 Yb | 5h | 20 ℃ | 50 |
| **CXCR5** | D6L3C | CST | 174 Yb | 5h | 20 ℃ | 100 |
| **CD27** | EPR8569 | Abcam | 175 Lu | O/N | 4℃ | 50 |
| **CD11c** | EP1347Y | Abcam | 176 Yb | 5h | 20 ℃ | 100 |
| **Vimentin** | D21H3 | CST | 194 Pt | O/N | 4℃ | 50 |
| **Keratin** | C11 and AE1/AE3 | BioLegend/CST | 198 Pt | O/N | 4℃ | 50 |
| **Histone H3** | D1H2 | CST | 209 Bi | O/N | 4℃ | 50 |

Temp=temperature; O/N = over night; 5h=5 hours.

**Supplementary Table 2.** The antibodies that were used for flow cytometry analysis.

| Target | Fluorochrome | Manufacturer | Catalogue number |
| --- | --- | --- | --- |
| CD38 | PerCP-Cy5.5 | BD | 566446 |
| IgD | PE-Cy7 | BD | 561314 |
| PD-1 | APC | Biolegend | 329907 |
| CD56 | APC-Cy7 | Biolegend | 362511 |
| CD3 | AF700 | eBioscience | 56-0038-42 |
| CD27 | BV421 | BD | 562513 |
| IgM | BV605 | BD | 562977 |
| CD86 | BV650 | BD | 563411 |
| CD19 | BV750 | BD | 747083 |
| CD40 | PE | BD | 555589 |
| HLA-DR | BV711 | BD | 563696 |
| HLA-G | PE-Dazzle594 | Biolegend | 335919 |
| IgG | BV786 | BD | 564230 |
| Ki67 (Intracellular) | FITC | Invitrogen | 11-5699-42 |
| Aqua L/D | BV510 | eBioscience | 65-0866-14 |

**Supplementary Table 3.** Average mRNA gene counts in the VA samples of three patient groups, by NanoString nCounter.

| **Gene Name** | **NanoString Probe ID** | **Class Name** | **Average PDAC patients (n=16)** | **StDev of PDAC** | **Average Colon AC (n=9)** | **StDev of: Colon AC** | **Averge Benign colon disease (n=9)** | **StDev of: Benign** |
| --- | --- | --- | --- | --- | --- | --- | --- | --- |
| **A2M** | NM_000014.4:1685 | Endogenous | 12481.35 | 2843.88 | 12339.45 | 3413.34 | 13563.93 | 4775.33 |
| **ABCB1** | NM_000927.3:3910 | Endogenous | 1471.45 | 1048.97 | 1008.88 | 166.37 | 1281.16 | 351.76 |
| **ABCF1** | NM_001090.2:850 | Housekeeping | 1482.96 | 258.29 | 1573.78 | 152.98 | 1658.22 | 192.23 |
| **ABL1** | NM_005157.3:3200 | Endogenous | 1446.74 | 371.99 | 1607.38 | 194 | 1792.2 | 489.5 |
| **ADA** | NM_000022.2:1300 | Endogenous | 654.44 | 244.89 | 703.02 | 193 | 527.28 | 154.06 |
| **ADORA2A** | NM_000675.3:1095 | Endogenous | 384.81 | 162 | 544.29 | 164.15 | 456.85 | 209.23 |
| **AGK** | NM_018238.3:816 | Housekeeping | 791.92 | 239.1 | 499.78 | 54.26 | 446.22 | 75.51 |
| **AICDA** | NM_020661.1:567 | Endogenous | 209.09 | 265.87 | 417.84 | 278.34 | 150.35 | 266.21 |
| **AIRE** | NM_000383.2:1864 | Endogenous | 193.36 | 183.26 | 232.4 | 128.99 | 111.87 | 70.3 |
| **AKT3** | NM_181690.1:755 | Endogenous | 2145.26 | 624.57 | 2126.27 | 250.86 | 2280.52 | 589.84 |
| **ALAS1** | NM_000688.4:1615 | Housekeeping | 1074.08 | 425.08 | 1027.09 | 235.29 | 1220.1 | 228.93 |
| **ALCAM** | NM_001627.3:789 | Endogenous | 873.32 | 153.38 | 617.81 | 83.68 | 666.86 | 174.97 |
| **AMBP** | NM_001633.3:597 | Endogenous | 66.28 | 144.65 | 20.04 | 35.67 | 16.43 | 5.22 |
| **AMICA1** | NM_153206.2:620 | Endogenous | 2992.18 | 894.19 | 2989.26 | 1016.5 | 3087.06 | 1507.7 |
| **AMMECR1L** | NM_001199140.1:3564 | Housekeeping | 1216.94 | 283.37 | 1018.79 | 127.76 | 994.34 | 123.96 |
| **ANP32B** | NM_006401.2:660 | Endogenous | 6493.25 | 1096.97 | 6929.7 | 723.58 | 5958.09 | 1630.89 |
| **ANXA1** | NM_000700.1:515 | Endogenous | 4079.7 | 1752.74 | 3598.59 | 575.93 | 4097.61 | 1039.11 |
| **APOE** | NM_000041.2:96 | Endogenous | 4717.47 | 1881.93 | 9841.06 | 3447.29 | 6946.25 | 2598.02 |
| **APP** | NM_000484.3:1725 | Endogenous | 9459.96 | 3547.08 | 10759.2 | 2938.51 | 12195.15 | 4538.2 |
| **ARG1** | NM_000045.2:505 | Endogenous | 68.67 | 162.46 | 19.02 | 47.84 | 16.36 | 5.96 |
| **ARG2** | NM_001172.3:1150 | Endogenous | 109.2 | 68.15 | 73.87 | 46.05 | 77.14 | 46.39 |
| **ATF1** | NM_005171.2:710 | Endogenous | 993.64 | 159.05 | 823.69 | 60.61 | 819.82 | 143.16 |
| **ATF2** | NM_001256090.1:336 | Endogenous | 2184.85 | 340.81 | 2003.89 | 209.43 | 1978.94 | 205.13 |
| **ATG10** | NM_001131028.1:985 | Endogenous | 392.14 | 124.2 | 314.66 | 52.76 | 288.14 | 58.47 |
| **ATG12** | NM_004707.2:25 | Endogenous | 165.66 | 113.18 | 118.22 | 103.75 | 91.58 | 18.47 |
| **ATG16L1** | NM_198890.2:1975 | Endogenous | 1111.84 | 404.55 | 1090.16 | 179.84 | 1089.2 | 126.6 |
| **ATG5** | NM_004849.2:1104 | Endogenous | 1594.61 | 385.14 | 900.59 | 161.74 | 902.51 | 205.43 |
| **ATG7** | NM_001136031.2:810 | Endogenous | 1048.52 | 146.88 | 982.94 | 104.8 | 899.4 | 153.36 |
| **ATM** | NM_000051.3:30 | Endogenous | 153.78 | 130.21 | 271.16 | 112.81 | 209.96 | 142.9 |
| **AXL** | NM_021913.2:2190 | Endogenous | 1150.1 | 258 | 1483.56 | 375.87 | 1698.17 | 574.95 |
| **BAGE** | NM_001187.1:399 | Endogenous | 104.17 | 101.79 | 35.63 | 34.53 | 25.15 | 14.61 |
| **BATF** | NM_006399.3:293 | Endogenous | 375.67 | 163.65 | 627.14 | 148.74 | 466.01 | 323.84 |
| **BAX** | NM_138761.3:342 | Endogenous | 2733.89 | 503.2 | 2563.08 | 193.01 | 2696.28 | 727.76 |
| **BCL10** | NM_003921.2:1250 | Endogenous | 3940.42 | 1951.75 | 3037.92 | 456.94 | 3344.29 | 507.72 |
| **BCL2** | NM_000657.2:947 | Endogenous | 1165.87 | 391.94 | 2954.61 | 756.49 | 3047.58 | 1682.67 |
| **BCL2L1** | NM_001191.2:260 | Endogenous | 2898 | 1346.89 | 2396.47 | 184.56 | 2573.11 | 337 |
| **BCL6** | NM_001706.2:675 | Endogenous | 1016.74 | 616.66 | 1564.84 | 613.5 | 1126.21 | 465.83 |
| **BID** | NM_001196.2:1875 | Endogenous | 141.56 | 95 | 115.89 | 36.8 | 108.85 | 29.93 |
| **BIRC5** | NM_001168.2:1215 | Endogenous | 869.26 | 292.28 | 1280.15 | 433.05 | 914.26 | 411.46 |
| **BLK** | NM_001715.2:990 | Endogenous | 547.13 | 281.94 | 831.82 | 252.77 | 745.76 | 442.29 |
| **BLNK** | NM_013314.2:930 | Endogenous | 821.23 | 253.8 | 996.82 | 159.6 | 836.5 | 286.95 |
| **BMI1** | NM_005180.5:1145 | Endogenous | 1098.8 | 178.38 | 964.53 | 134.98 | 982.62 | 176.71 |
| **BST1** | NM_004334.2:710 | Endogenous | 479.66 | 237.62 | 343.42 | 81.85 | 308.15 | 110 |
| **BST2** | NM_004335.2:560 | Endogenous | 2275.48 | 651.2 | 2668.27 | 609.97 | 2601.2 | 435.05 |
| **BTK** | NM_000061.1:570 | Endogenous | 812.6 | 357.89 | 1259.29 | 366.11 | 883.06 | 489.94 |
| **BTLA** | NM_181780.2:305 | Endogenous | 1758.53 | 1239.65 | 1985.51 | 712.42 | 1482.61 | 908.34 |
| **C1QA** | NM_015991.2:718 | Endogenous | 2548.61 | 1105.78 | 2971.54 | 931.18 | 2828.34 | 950.86 |
| **C1QB** | NM_000491.3:819 | Endogenous | 5856.93 | 1613.5 | 7678.06 | 3422.99 | 7536.57 | 3669.31 |
| **C1QBP** | NM_001212.3:745 | Endogenous | 5135.52 | 1248.94 | 5656.33 | 880.06 | 5559.94 | 1288.44 |
| **C1R** | NM_001733.4:760 | Endogenous | 6424.2 | 1981.22 | 6503.3 | 1540.71 | 6032.86 | 2406.06 |
| **C1S** | NM_001734.2:775 | Endogenous | 4781.37 | 1911.5 | 5497.77 | 1420.84 | 4722.58 | 1694.08 |
| **C2** | NM_000063.3:1075 | Endogenous | 843.65 | 231.33 | 1000.13 | 309.47 | 886.45 | 260.4 |
| **C3** | NM_000064.2:4396 | Endogenous | 8513.67 | 4066.11 | 8334.18 | 3376.74 | 5825.17 | 2859.97 |
| **C3AR1** | NM_004054.2:415 | Endogenous | 579.62 | 147.98 | 600.34 | 59.69 | 550.43 | 166.38 |
| **C4B** | NM_001002029.3:4437 | Endogenous | 1622.36 | 760.56 | 2945.24 | 1184.82 | 3420.6 | 1707.74 |
| **C4BPA** | NM_000715.3:690 | Endogenous | 123.67 | 110.35 | 70.74 | 73.81 | 72.11 | 51.88 |
| **C5** | NM_001735.2:2592 | Endogenous | 250.2 | 169.25 | 220.33 | 53.85 | 177.22 | 31.04 |
| **C6** | NM_000065.2:3170 | Endogenous | 182.05 | 234.85 | 70.12 | 65.04 | 47.94 | 45.45 |
| **C7** | NM_000587.2:310 | Endogenous | 4132.51 | 1956.5 | 4206.58 | 1682.96 | 4134.13 | 3252.96 |
| **C8A** | NM_000562.2:690 | Endogenous | 65.99 | 160.56 | 17.08 | 32.75 | 11.78 | 4.63 |
| **C8B** | NM_000066.2:620 | Endogenous | 63.55 | 121.45 | 23.98 | 27.28 | 18.15 | 5.17 |
| **C8G** | NM_000606.2:407 | Endogenous | 85.22 | 73.85 | 61.12 | 29.37 | 72.56 | 77.16 |
| **C9** | NM_001737.3:602 | Endogenous | 147.05 | 266 | 66.36 | 47.35 | 38.53 | 14.56 |
| **CAMP** | NM_004345.3:220 | Endogenous | 95.78 | 126.57 | 73.65 | 68.58 | 42.76 | 26.87 |
| **CARD11** | NM_032415.2:1075 | Endogenous | 1192.77 | 487.5 | 2065.17 | 497.27 | 1837.87 | 1344.75 |
| **CARD9** | NM_052813.4:1525 | Endogenous | 168.92 | 110.03 | 194.7 | 47.38 | 177.07 | 95.08 |
| **CASP1** | NM_001223.3:971 | Endogenous | 1904.24 | 922.29 | 1854.18 | 746.85 | 1830.31 | 447.81 |
| **CASP10** | NM_032977.3:20 | Endogenous | 138.12 | 237.74 | 60.79 | 115.09 | 44.36 | 15.72 |
| **CASP3** | NM_032991.2:685 | Endogenous | 1768.6 | 327.12 | 1435.23 | 232.82 | 1404.7 | 170.44 |
| **CASP8** | NM_001228.4:301 | Endogenous | 2720.74 | 559.79 | 2319.49 | 426.95 | 2558.73 | 812.89 |
| **CC2D1B** | NM_032449.2:4182 | Housekeeping | 196.13 | 179.52 | 187.59 | 49.6 | 184.87 | 55.27 |
| **CCL1** | NM_002981.1:157 | Endogenous | 76.23 | 190.64 | 20.7 | 67.06 | 10.51 | 2.4 |
| **CCL11** | NM_002986.2:378 | Endogenous | 2564.08 | 1028.06 | 2171.12 | 1556.76 | 1996.57 | 1338.89 |
| **CCL13** | NM_005408.2:320 | Endogenous | 506.61 | 506.64 | 399.78 | 251.19 | 352.32 | 231.46 |
| **CCL14** | NM_032963.3:274 | Endogenous | 951.89 | 345.19 | 933.28 | 234.39 | 856.17 | 484.46 |
| **CCL15** | NM_032965.3:112 | Endogenous | 437.29 | 127.71 | 202.66 | 120.91 | 242.47 | 103.83 |
| **CCL16** | NM_004590.2:367 | Endogenous | 111.64 | 201.1 | 38.18 | 52.11 | 29.43 | 11.81 |
| **CCL17** | NM_002987.2:229 | Endogenous | 221.01 | 110.05 | 230.44 | 79.97 | 174.4 | 181.23 |
| **CCL18** | NM_002988.2:585 | Endogenous | 1397.28 | 765.45 | 754.28 | 464.02 | 459.41 | 423.97 |
| **CCL19** | NM_006274.2:401 | Endogenous | 12559.62 | 11307.81 | 26564.4 | 24641.61 | 16164.08 | 20006.22 |
| **CCL2** | NM_002982.3:123 | Endogenous | 1113.06 | 699.63 | 1858.46 | 1180.13 | 1680.1 | 1031.93 |
| **CCL20** | NM_004591.1:35 | Endogenous | 1751.23 | 1455.88 | 2195.33 | 2450.71 | 1684.1 | 909.64 |
| **CCL21** | NM_002989.2:180 | Endogenous | 10530.92 | 11345.41 | 12418.73 | 4625.21 | 10792.23 | 7768.44 |
| **CCL22** | NM_002990.3:797 | Endogenous | 356.16 | 133.3 | 598.62 | 313.38 | 370.32 | 302.56 |
| **CCL23** | NM_145898.1:336 | Endogenous | 225.24 | 261.76 | 380.43 | 278.75 | 503.32 | 343.53 |
| **CCL24** | NM_002991.2:18 | Endogenous | 251.52 | 387.46 | 205.23 | 74.84 | 270.54 | 432.86 |
| **CCL25** | NM_005624.2:325 | Endogenous | 61.09 | 108.34 | 29.59 | 38.46 | 20.16 | 10.03 |
| **CCL26** | NM_006072.4:184 | Endogenous | 117.42 | 179.63 | 48.63 | 49.2 | 33.75 | 17.13 |
| **CCL27** | NM_006664.2:304 | Endogenous | 52.99 | 66.99 | 24.85 | 36.55 | 21.69 | 6.81 |
| **CCL28** | NM_148672.2:100 | Endogenous | 614.33 | 443.08 | 350.25 | 312.56 | 528.42 | 314.45 |
| **CCL3** | NM_002983.2:159 | Endogenous | 437.52 | 411.67 | 364.88 | 156.17 | 258.2 | 147.78 |
| **CCL3L1** | NM_021006.4:421 | Endogenous | 402.06 | 337.49 | 338.41 | 143.8 | 245.87 | 198.82 |
| **CCL4** | NM_002984.2:35 | Endogenous | 589.64 | 242.78 | 728.91 | 192.24 | 499.95 | 343.37 |
| **CCL5** | NM_002985.2:280 | Endogenous | 2244.07 | 1000.17 | 3277.66 | 930.43 | 3565.89 | 980.13 |
| **CCL7** | NM_006273.2:120 | Endogenous | 20.31 | 4.5 | 19.87 | 5.93 | 19.36 | 11.02 |
| **CCL8** | NM_005623.2:689 | Endogenous | 197.6 | 171.69 | 144.45 | 67.34 | 141.9 | 115.99 |
| **CCND3** | NM_001760.2:1215 | Endogenous | 1505.35 | 480.34 | 1840.09 | 575.02 | 1651.91 | 557.74 |
| **CCR1** | NM_001295.2:535 | Endogenous | 422.96 | 129.55 | 301.46 | 91.22 | 243.84 | 91.96 |
| **CCR2** | NM_001123041.2:743 | Endogenous | 511.52 | 231.61 | 504.37 | 221.8 | 518.66 | 230.93 |
| **CCR3** | NM_001837.2:980 | Endogenous | 143.79 | 173.77 | 45.61 | 51.98 | 30.69 | 15.86 |
| **CCR4** | NM_005508.4:35 | Endogenous | 250.81 | 173.96 | 213.43 | 101.42 | 154.25 | 67.49 |
| **CCR5** | NM_000579.1:2730 | Endogenous | 360.91 | 163.82 | 487.2 | 113.36 | 391.52 | 81.06 |
| **CCR6** | NM_031409.2:935 | Endogenous | 661.79 | 330.75 | 683.12 | 224.24 | 509.44 | 243.4 |
| **CCR7** | NM_001838.2:1610 | Endogenous | 971.28 | 690.69 | 3029.66 | 1250.53 | 2304.5 | 2633.79 |
| **CCR9** | NM_031200.1:1095 | Endogenous | 111.14 | 177.95 | 88.2 | 54.8 | 57.74 | 34.83 |
| **CCRL2** | NM_003965.4:1110 | Endogenous | 581.5 | 216.32 | 313.77 | 122.83 | 318.41 | 60.31 |
| **CD14** | NM_000591.2:885 | Endogenous | 1460.98 | 318.31 | 1619.26 | 376.08 | 1828.56 | 944.18 |
| **CD160** | NM_007053.2:500 | Endogenous | 206.52 | 227.3 | 119.89 | 89.46 | 102.38 | 41.14 |
| **CD163** | NM_004244.4:1630 | Endogenous | 1284.91 | 630.8 | 976.1 | 248.45 | 1111.12 | 590.14 |
| **CD164** | NM_006016.4:2575 | Endogenous | 9993.33 | 7729.65 | 6435.67 | 2174.14 | 6570.39 | 2745.56 |
| **CD180** | NM_005582.2:1036 | Endogenous | 1384.77 | 684.01 | 1775.36 | 548.98 | 1332.66 | 771.22 |
| **CD19** | NM_001770.4:1770 | Endogenous | 1558.57 | 1424.68 | 5746.47 | 2424.77 | 3850.73 | 2641.6 |
| **CD1A** | NM_001763.2:1815 | Endogenous | 126.14 | 138.03 | 146.94 | 61.83 | 82.07 | 93.66 |
| **CD1B** | NM_001764.2:1045 | Endogenous | 28.71 | 18.73 | 42.17 | 43.94 | 19.12 | 23.13 |
| **CD1C** | NM_001765.2:750 | Endogenous | 747.8 | 418.72 | 969.81 | 380.99 | 590.2 | 461.54 |
| **CD1D** | NM_001766.3:1428 | Endogenous | 396.11 | 115.06 | 637.86 | 191.99 | 467.63 | 205.88 |
| **CD1E** | NM_001042583.1:180 | Endogenous | 133.89 | 131.56 | 99.44 | 61.88 | 53 | 35.14 |
| **CD2** | NM_001767.3:687 | Endogenous | 429.57 | 184.63 | 542.22 | 148.27 | 494.93 | 162.71 |
| **CD200** | NM_005944.5:665 | Endogenous | 807.93 | 197.67 | 816.01 | 199.16 | 657.35 | 163.5 |
| **CD207** | NM_015717.2:995 | Endogenous | 132.96 | 144.93 | 103.9 | 26.61 | 63.29 | 50.66 |
| **CD209** | NM_021155.2:1532 | Endogenous | 561.12 | 195.78 | 461.81 | 234.75 | 446.32 | 127.81 |
| **CD22** | NM_001771.2:2515 | Endogenous | 3258.67 | 2534.26 | 10105.52 | 5499.49 | 5876.37 | 4414.52 |
| **CD24** | NM_013230.2:95 | Endogenous | 25310.42 | 13915.76 | 24183.55 | 14874.35 | 38262.95 | 18193.37 |
| **CD244** | NM_016382.2:1150 | Endogenous | 111.03 | 55.94 | 96.63 | 39.94 | 114.32 | 36.15 |
| **CD247** | NM_198053.1:1490 | Endogenous | 1296.59 | 627.84 | 1608.41 | 356.01 | 1327.05 | 521.5 |
| **CD27** | NM_001242.4:330 | Endogenous | 2534.24 | 1030.82 | 3978.87 | 370.1 | 3324.92 | 1032.23 |
| **CD274** | NM_014143.3:1245 | Endogenous | 171.63 | 68.08 | 215.61 | 83.56 | 174.46 | 102.55 |
| **CD276** | NM_001024736.1:2120 | Endogenous | 1099.67 | 551.14 | 1036.07 | 162.76 | 1115.19 | 322.41 |
| **CD28** | NM_001243078.1:2065 | Endogenous | 497.68 | 278.57 | 785.91 | 272.78 | 590.49 | 240.78 |
| **CD33** | NM_001177608.1:730 | Endogenous | 75.9 | 26.48 | 104.88 | 39.2 | 104.18 | 29.56 |
| **CD34** | NM_001025109.1:1580 | Endogenous | 188.05 | 73.33 | 323.64 | 94.91 | 330.04 | 112.82 |
| **CD36** | NM_001001548.2:705 | Endogenous | 1452.89 | 445.01 | 1004.2 | 771.65 | 1154.36 | 292.8 |
| **CD37** | NM_001774.2:535 | Endogenous | 738.31 | 400.13 | 1923.35 | 732.22 | 1582.35 | 996.09 |
| **CD38** | NM_001775.2:460 | Endogenous | 1010.34 | 423.59 | 1258.69 | 270.24 | 842.03 | 231.61 |
| **CD3D** | NM_000732.4:110 | Endogenous | 3321.38 | 1641.06 | 3378.39 | 816.01 | 2764.98 | 1288.81 |
| **CD3E** | NM_000733.2:75 | Endogenous | 1939.05 | 1022.5 | 3021.31 | 718.26 | 2578.16 | 1175.67 |
| **CD3EAP** | NM_012099.1:555 | Endogenous | 208.81 | 146.82 | 223.8 | 95.73 | 200.65 | 62.04 |
| **CD3G** | NM_000073.2:404 | Endogenous | 687.95 | 366.2 | 503.54 | 164.19 | 404.1 | 241.69 |
| **CD4** | NM_000616.4:975 | Endogenous | 1362.42 | 652.5 | 2387.05 | 715.84 | 2091.38 | 819.9 |
| **CD40** | NM_001250.4:1265 | Endogenous | 1880.1 | 912.64 | 3039.55 | 1116.74 | 1961.37 | 782.81 |
| **CD40LG** | NM_000074.2:1225 | Endogenous | 565.8 | 278.53 | 828.21 | 308.45 | 775.33 | 283.86 |
| **CD44** | NM_001001392.1:429 | Endogenous | 6922.3 | 1856.1 | 11683.3 | 2988.02 | 12172.35 | 8400.58 |
| **CD46** | NM_172350.1:365 | Endogenous | 7665.33 | 1350.51 | 4410.61 | 952.74 | 5279.64 | 1154.18 |
| **CD47** | NM_001777.3:897 | Endogenous | 6565.61 | 1244.61 | 5954.65 | 675.87 | 5874.34 | 1090.77 |
| **CD48** | NM_001778.2:270 | Endogenous | 2678.3 | 1429.04 | 3984.7 | 874.04 | 3411.33 | 1343.48 |
| **CD5** | NM_014207.2:1295 | Endogenous | 730.82 | 447.07 | 1059.97 | 406.2 | 930.65 | 412.61 |
| **CD53** | NM_001040033.1:835 | Endogenous | 8690.38 | 3937.3 | 8676.9 | 1940.33 | 6647.48 | 2497.7 |
| **CD55** | NM_000574.3:101 | Endogenous | 255.09 | 194.14 | 232.88 | 149.56 | 168.96 | 47.38 |
| **CD58** | NM_001779.2:478 | Endogenous | 1311.51 | 282.74 | 914.82 | 133.95 | 949.54 | 152.85 |
| **CD59** | NM_000611.4:730 | Endogenous | 8243.62 | 4661.07 | 9406.39 | 1658.31 | 9818.28 | 2270.69 |
| **CD6** | NM_006725.3:1280 | Endogenous | 606.86 | 273.81 | 937.19 | 317.76 | 769.09 | 310.23 |
| **CD63** | NM_001780.4:350 | Endogenous | 26659.4 | 10611.15 | 30258.61 | 9783.24 | 38378.59 | 19118.06 |
| **CD68** | NM_001251.2:1140 | Endogenous | 1667.27 | 328.51 | 2228.61 | 784.73 | 2216.34 | 754.1 |
| **CD7** | NM_006137.6:440 | Endogenous | 527.35 | 295.01 | 1056.1 | 387.24 | 909.98 | 537.72 |
| **CD70** | NM_001252.2:190 | Endogenous | 132.2 | 178.51 | 155.83 | 44.41 | 135.52 | 95.88 |
| **CD74** | NM_001025159.1:964 | Endogenous | 40397.98 | 14320.11 | 75714.31 | 15216.58 | 55610.2 | 24951.8 |
| **CD79A** | NM_001783.3:695 | Endogenous | 7729.83 | 3720.93 | 16169.87 | 3658.71 | 12423.87 | 8841.77 |
| **CD79B** | NM_021602.2:24 | Endogenous | 2908.33 | 2002.76 | 6235.85 | 3671.46 | 4005.81 | 2351.73 |
| **CD80** | NM_005191.3:1288 | Endogenous | 414.04 | 180.39 | 346.11 | 122.54 | 248.53 | 138.48 |
| **CD81** | NM_004356.3:735 | Endogenous | 17710.53 | 3532.37 | 19282.53 | 2432.99 | 17879.44 | 5057.42 |
| **CD83** | NM_004233.3:1960 | Endogenous | 1980.63 | 1517.7 | 4619.47 | 2444.66 | 2863.89 | 1832.05 |
| **CD84** | NM_001184879.1:28 | Endogenous | 2046.85 | 866.65 | 1532.58 | 199.21 | 1279.87 | 450.53 |
| **CD86** | NM_175862.3:1265 | Endogenous | 362.64 | 151.46 | 299.57 | 74.34 | 204.16 | 59.23 |
| **CD8A** | NM_001768.5:1320 | Endogenous | 763.91 | 455.72 | 1064.38 | 458.94 | 1115.54 | 344.51 |
| **CD8B** | NM_004931.3:440 | Endogenous | 368.89 | 240.63 | 357.9 | 161.09 | 357.29 | 141.93 |
| **CD9** | NM_001769.2:405 | Endogenous | 7957.75 | 5014.86 | 8078.15 | 4103.88 | 11983.24 | 4522.71 |
| **CD96** | NM_005816.4:1355 | Endogenous | 1319.25 | 625.88 | 1267.6 | 283.2 | 1163.28 | 422.33 |
| **CD97** | NM_078481.2:1370 | Endogenous | 2807.89 | 588.74 | 2832.31 | 289.8 | 2832.5 | 348.32 |
| **CD99** | NM_002414.3:625 | Endogenous | 7616.44 | 1684.09 | 9058.71 | 1869.75 | 9518.8 | 2099.41 |
| **CDH1** | NM_004360.2:535 | Endogenous | 6418.92 | 4386.17 | 3952.3 | 2390.45 | 6694.32 | 2196.12 |
| **CDH5** | NM_001795.3:3405 | Endogenous | 496.04 | 178.89 | 398.65 | 112.33 | 325.87 | 85.39 |
| **CDK1** | NM_001786.4:178 | Endogenous | 1180.3 | 508.32 | 1280.04 | 468.7 | 814.95 | 358.02 |
| **CDKN1A** | NM_000389.2:1975 | Endogenous | 1447.04 | 2059.93 | 1523.71 | 641.75 | 1935.04 | 578.41 |
| **CEACAM1** | NM_001712.3:2455 | Endogenous | 2237.39 | 3594.03 | 2037.21 | 3192.55 | 3598.67 | 2925.32 |
| **CEACAM6** | NM_002483.4:1217 | Endogenous | 3430.94 | 13106.32 | 1815.02 | 2462.3 | 3671.75 | 2129.57 |
| **CEACAM8** | NM_001816.3:825 | Endogenous | 61.71 | 128.07 | 21.49 | 29.14 | 15.82 | 7.11 |
| **CEBPB** | NM_005194.2:1420 | Endogenous | 1657.83 | 497.9 | 1607.81 | 674.75 | 1357.85 | 555.4 |
| **CFB** | NM_001710.5:2029 | Endogenous | 950.6 | 362.25 | 875.09 | 406.96 | 865.71 | 456.95 |
| **CFD** | NM_001928.2:859 | Endogenous | 1071.93 | 713.69 | 1090.3 | 972.08 | 1442.49 | 811.45 |
| **CFI** | NM_000204.3:1780 | Endogenous | 576.6 | 138.27 | 621.3 | 114.05 | 596.15 | 152.31 |
| **CFP** | NM_002621.2:380 | Endogenous | 303.8 | 130.19 | 387.98 | 157.44 | 320.32 | 214.9 |
| **CHIT1** | NM_003465.2:410 | Endogenous | 170.95 | 246.44 | 102.3 | 53.26 | 56.73 | 17.63 |
| **CHUK** | NM_001278.3:860 | Endogenous | 1032.95 | 388.9 | 1550.06 | 119.14 | 1620.05 | 424.83 |
| **CKLF** | NM_181640.2:275 | Endogenous | 2260.97 | 731.55 | 1969.35 | 297.4 | 1967.04 | 279.6 |
| **CLEC4A** | NM_194448.2:388 | Endogenous | 533.87 | 148 | 676.23 | 205.17 | 593.34 | 268.53 |
| **CLEC4C** | NM_203503.1:570 | Endogenous | 70.26 | 68.81 | 142.62 | 68.52 | 63.14 | 78.12 |
| **CLEC5A** | NM_013252.2:615 | Endogenous | 24.21 | 11.91 | 24.03 | 7.64 | 24.28 | 15.47 |
| **CLEC6A** | NM_001007033.1:342 | Endogenous | 80.29 | 91.28 | 49.1 | 55.74 | 25.7 | 16.17 |
| **CLEC7A** | NM_197954.2:55 | Endogenous | 363.28 | 169.6 | 182.03 | 99.25 | 157.61 | 56.15 |
| **CLU** | NM_001831.2:2340 | Endogenous | 133.68 | 174.9 | 56.42 | 54.92 | 44.98 | 25.79 |
| **CMA1** | NM_001836.2:561 | Endogenous | 129.07 | 131.58 | 161.84 | 142.96 | 134.65 | 151.41 |
| **CMKLR1** | NM_004072.1:770 | Endogenous | 1081.04 | 261.58 | 1024.92 | 205.97 | 855.63 | 203.41 |
| **CNOT10** | NM_001256741.1:1962 | Housekeeping | 700.84 | 402.38 | 308 | 54.41 | 306.94 | 52.55 |
| **CNOT4** | NM_001190848.1:795 | Housekeeping | 1218.37 | 134.59 | 1201.16 | 117.57 | 1226.63 | 190.07 |
| **COG7** | NM_153603.3:1492 | Housekeeping | 1210.43 | 192.97 | 834.04 | 161.67 | 969.67 | 130.17 |
| **COL3A1** | NM_000090.3:180 | Endogenous | 17270.88 | 4947.54 | 21790.23 | 10645.08 | 22015.19 | 6458.04 |
| **COLEC12** | NM_130386.2:900 | Endogenous | 467.95 | 180.87 | 386.7 | 59.09 | 391.31 | 125.39 |
| **CR1** | NM_000651.4:1695 | Endogenous | 2310.01 | 1194.92 | 3924.69 | 2074.5 | 2413.23 | 1326.85 |
| **CR2** | NM_001006658.1:485 | Endogenous | 2378.71 | 2324.16 | 3929.33 | 2022.67 | 1903.46 | 1580.43 |
| **CREB1** | NM_004379.3:4855 | Endogenous | 1699.87 | 316.98 | 2307.84 | 491.19 | 1960.98 | 542.58 |
| **CREB5** | NM_182898.2:1885 | Endogenous | 260.94 | 174.83 | 179.27 | 80.1 | 155.75 | 43.23 |
| **CREBBP** | NM_004380.2:8855 | Endogenous | 154.49 | 84.98 | 179.22 | 37.42 | 185.34 | 25.91 |
| **CRP** | NM_000567.2:1521 | Endogenous | 49.9 | 127.96 | 17.26 | 27.2 | 12.32 | 4.51 |
| **CSF1** | NM_000757.4:823 | Endogenous | 741.82 | 177.35 | 674.78 | 171.72 | 613.82 | 158.97 |
| **CSF1R** | NM_005211.2:3775 | Endogenous | 187.23 | 102.63 | 257.05 | 50.76 | 213.27 | 44.61 |
| **CSF2** | NM_000758.2:475 | Endogenous | 43.73 | 51.97 | 27.77 | 27.19 | 22.41 | 13.9 |
| **CSF2RB** | NM_000395.2:3300 | Endogenous | 2419.67 | 803.76 | 4402.03 | 1204.77 | 3599.15 | 1847.16 |
| **CSF3** | NM_000759.3:851 | Endogenous | 35.65 | 41.51 | 25.27 | 15.96 | 21.46 | 7.02 |
| **CSF3R** | NM_156038.2:90 | Endogenous | 350.97 | 293.5 | 358.86 | 163.17 | 335.13 | 465.43 |
| **CT45A1** | NM_001017417.1:866 | Endogenous | 187.85 | 1164.24 | 34.32 | 141.81 | 26.76 | 9.61 |
| **CTAG1B** | NM_001327.2:285 | Endogenous | 26.54 | 9.44 | 30.39 | 312.63 | 20.75 | 7.51 |
| **CTAGE1** | NM_172241.2:3365 | Endogenous | 74.24 | 220.3 | 19.17 | 48.41 | 13.01 | 3.07 |
| **CTCFL** | NM_001269042.1:1100 | Endogenous | 106.88 | 205.89 | 32.03 | 67.41 | 13.96 | 10.45 |
| **CTLA4** | NM_005214.3:405 | Endogenous | 945.95 | 452.28 | 1013.52 | 439.96 | 586.04 | 532.13 |
| **CTSG** | NM_001911.2:160 | Endogenous | 290.54 | 177.39 | 333.2 | 122.28 | 360.25 | 536.69 |
| **CTSH** | NM_004390.3:344 | Endogenous | 4056.98 | 1992.48 | 3880.5 | 849.74 | 2960.49 | 1036.8 |
| **CTSL** | NM_001912.4:1072 | Endogenous | 1027.92 | 360.57 | 966.58 | 260.19 | 909.75 | 303.27 |
| **CTSS** | NM_004079.3:685 | Endogenous | 11001.47 | 3028.25 | 7576.85 | 1608.71 | 7279.79 | 1760.49 |
| **CTSW** | NM_001335.3:1075 | Endogenous | 266.65 | 170.59 | 239.39 | 163.17 | 328.36 | 150.39 |
| **CX3CL1** | NM_002996.3:140 | Endogenous | 483.52 | 131.02 | 341.13 | 60.92 | 289.19 | 252.18 |
| **CX3CR1** | NM_001337.3:1040 | Endogenous | 243.44 | 149.05 | 246.25 | 66.83 | 217.74 | 172.85 |
| **CXCL1** | NM_001511.1:742 | Endogenous | 1371.1 | 995.73 | 2940.78 | 2622.12 | 1366.97 | 1168.81 |
| **CXCL10** | NM_001565.1:40 | Endogenous | 569.5 | 467.7 | 411.68 | 259.47 | 187.56 | 273.36 |
| **CXCL11** | NM_005409.4:282 | Endogenous | 238.97 | 195.74 | 220.86 | 191.03 | 108.26 | 196.98 |
| **CXCL12** | NM_000609.5:210 | Endogenous | 6110.15 | 5073.4 | 5084.9 | 892.79 | 5110.31 | 2904.13 |
| **CXCL13** | NM_006419.2:210 | Endogenous | 2627.93 | 4652.8 | 6883.93 | 3046.08 | 4299.41 | 3952.03 |
| **CXCL14** | NM_004887.4:1125 | Endogenous | 15427.1 | 5033.62 | 18667.7 | 4649.26 | 15362.18 | 7064.17 |
| **CXCL16** | NM_001100812.1:850 | Endogenous | 893.65 | 431.76 | 1172.49 | 408.55 | 1253.47 | 230.91 |
| **CXCL2** | NM_002089.3:854 | Endogenous | 376.22 | 241.08 | 762.34 | 893.49 | 449.56 | 441.14 |
| **CXCL3** | NM_002090.2:540 | Endogenous | 658.48 | 725.57 | 730.56 | 1823.67 | 388.94 | 458.74 |
| **CXCL5** | NM_002994.3:250 | Endogenous | 157.95 | 584.78 | 156.63 | 751.82 | 85.31 | 337.72 |
| **CXCL6** | NM_002993.3:539 | Endogenous | 385.07 | 196.12 | 571.5 | 294.26 | 207.57 | 239.09 |
| **CXCL9** | NM_002416.1:1975 | Endogenous | 1265.28 | 1053.4 | 2618.7 | 1811.92 | 1201.36 | 1985.35 |
| **CXCR1** | NM_000634.2:1950 | Endogenous | 91.86 | 52.82 | 38.83 | 36.37 | 39.64 | 31.41 |
| **CXCR2** | NM_001557.2:2055 | Endogenous | 165.79 | 197.56 | 82.58 | 82.39 | 58.69 | 123.64 |
| **CXCR3** | NM_001504.1:80 | Endogenous | 439.94 | 183.35 | 571.95 | 244.8 | 496.13 | 188.25 |
| **CXCR4** | NM_003467.2:1335 | Endogenous | 12900.98 | 11074.55 | 32280.03 | 18497.36 | 18325.6 | 18440.24 |
| **CXCR5** | NM_001716.3:2618 | Endogenous | 722.7 | 496.76 | 1805.76 | 1000.99 | 1244.69 | 893.04 |
| **CXCR6** | NM_006564.1:95 | Endogenous | 553.97 | 276.46 | 405.35 | 87.01 | 354.13 | 141.15 |
| **CYBB** | NM_000397.3:2686 | Endogenous | 3505.87 | 1359.69 | 5353.58 | 1418.03 | 4052.76 | 1574.13 |
| **CYFIP2** | NM_001037332.2:4043 | Endogenous | 2590.27 | 905.91 | 3047.28 | 615.2 | 2499.15 | 817.85 |
| **CYLD** | NM_015247.1:2890 | Endogenous | 2954.49 | 752.42 | 3475.13 | 452.71 | 3261.07 | 1081.86 |
| **DDX43** | NM_018665.2:1255 | Endogenous | 91.7 | 65.11 | 56.66 | 39.95 | 48 | 16.38 |
| **DDX50** | NM_024045.1:1185 | Housekeeping | 1011.73 | 278.09 | 1214.12 | 164.5 | 1102.24 | 142.52 |
| **DDX58** | NM_014314.3:2130 | Endogenous | 847.19 | 247.97 | 1032.33 | 231.63 | 1090.61 | 272.19 |
| **DEFB1** | NM_005218.3:40 | Endogenous | 101.45 | 123.74 | 53.84 | 49.91 | 46.93 | 23.78 |
| **DHX16** | NM_001164239.1:2490 | Housekeeping | 858.71 | 199.66 | 1070.94 | 148.94 | 1129.11 | 156.1 |
| **DMBT1** | NM_007329.2:3712 | Endogenous | 3138.55 | 5533.56 | 2091.21 | 10631.78 | 3213.9 | 16496.78 |
| **DNAJC14** | NM_032364.5:1166 | Housekeeping | 480.52 | 103.48 | 698.84 | 56.53 | 708.75 | 211.57 |
| **DOCK9** | NM_001130048.1:1020 | Endogenous | 1657.57 | 307.46 | 1787.54 | 331.8 | 1918.96 | 257.49 |
| **DPP4** | NM_001935.3:2700 | Endogenous | 323.9 | 138.88 | 289.97 | 43.22 | 327.99 | 75.21 |
| **DUSP4** | NM_057158.2:3115 | Endogenous | 271.58 | 384.95 | 331.16 | 166.24 | 215.75 | 117.1 |
| **DUSP6** | NM_001946.2:1535 | Endogenous | 1564.53 | 890.37 | 1466.01 | 396.7 | 1449.73 | 478.17 |
| **EBI3** | NM_005755.2:485 | Endogenous | 119.98 | 49.92 | 296.87 | 91.74 | 249.56 | 96.59 |
| **ECSIT** | NM_001142464.2:1318 | Endogenous | 897.29 | 149.16 | 691.16 | 94.12 | 786.94 | 91.47 |
| **EDC3** | NM_001142443.1:925 | Housekeeping | 1036.67 | 210.53 | 1252.09 | 157.23 | 1298.55 | 178.96 |
| **EGR1** | NM_001964.2:1505 | Endogenous | 390.52 | 434.74 | 1768.99 | 1531.08 | 1260.83 | 2830.72 |
| **EGR2** | NM_000399.3:1891 | Endogenous | 235.83 | 150.56 | 281.14 | 155.48 | 176.31 | 169.44 |
| **EIF2B4** | NM_172195.3:1390 | Housekeeping | 1369.29 | 93.77 | 1172.7 | 65.3 | 1308.48 | 153.24 |
| **ELANE** | NM_001972.2:195 | Endogenous | 28.64 | 10.09 | 26.56 | 8.87 | 28.57 | 6.97 |
| **ELK1** | NM_005229.3:2350 | Endogenous | 337.52 | 75.7 | 390.65 | 91.81 | 381.37 | 131.59 |
| **ENG** | NM_001114753.1:1480 | Endogenous | 1591.75 | 329.64 | 1775.92 | 436.75 | 1758.35 | 458.22 |
| **ENTPD1** | NM_001098175.1:8830 | Endogenous | 957.12 | 320.88 | 1438.76 | 371.08 | 1201.95 | 240.97 |
| **EOMES** | NM_005442.2:1670 | Endogenous | 122.94 | 66.39 | 167.84 | 43.14 | 136.21 | 56.21 |
| **EP300** | NM_001429.2:715 | Endogenous | 3421.25 | 437.92 | 3487.31 | 330.17 | 3341.13 | 548.91 |
| **EPCAM** | NM_002354.1:415 | Endogenous | 14246.54 | 10251.6 | 9575.79 | 7933.19 | 18581.06 | 6533.03 |
| **ERCC3** | NM_000122.1:1950 | Housekeeping | 747.53 | 98.16 | 1035.67 | 140.75 | 970.07 | 108.95 |
| **ETS1** | NM_005238.3:4625 | Endogenous | 8289.64 | 3457.36 | 8738.54 | 1702.63 | 6707.38 | 3077.5 |
| **EWSR1** | NM_013986.3:452 | Endogenous | 4869.27 | 492 | 4786.18 | 598.63 | 4912.9 | 730.13 |
| **F12** | NM_000505.3:1862 | Endogenous | 162.42 | 89.4 | 113.57 | 54.61 | 145.93 | 66.07 |
| **F13A1** | NM_000129.3:3196 | Endogenous | 2646.67 | 1495.05 | 2467.33 | 1233.57 | 2687 | 1526.7 |
| **F2RL1** | NM_005242.3:940 | Endogenous | 1140.33 | 1337.49 | 1078.91 | 911.9 | 2121.23 | 1183.44 |
| **FADD** | NM_003824.2:1560 | Endogenous | 243.97 | 122.74 | 301.01 | 26.22 | 308.08 | 98.31 |
| **FAS** | NM_000043.3:90 | Endogenous | 144.75 | 38.66 | 138.32 | 42.36 | 121.66 | 24.8 |
| **FCER1A** | NM_002001.2:114 | Endogenous | 254.75 | 159.13 | 319.24 | 89.43 | 280.88 | 236.12 |
| **FCER1G** | NM_004106.1:36 | Endogenous | 1542.7 | 584.49 | 2141.61 | 807.44 | 2185.3 | 793.44 |
| **FCER2** | NM_002002.4:420 | Endogenous | 531.54 | 484.64 | 1447.3 | 1253.73 | 886.81 | 948.63 |
| **FCF1** | NM_015962.4:1022 | Housekeeping | 6023.59 | 1675 | 5737.86 | 816.2 | 5259.61 | 888.36 |
| **FCGR1A** | NM_000566.3:1545 | Endogenous | 120.59 | 122.22 | 83.93 | 78.12 | 62.87 | 25.86 |
| **FCGR2A** | NM_021642.3:60 | Endogenous | 1230.18 | 409.94 | 996 | 441.2 | 1119.97 | 453.17 |
| **FCGR2B** | NM_001002273.1:870 | Endogenous | 1137.35 | 371.75 | 1146.34 | 229.22 | 1090.78 | 450.31 |
| **FCGR3A** | NM_000569.6:1644 | Endogenous | 519.11 | 396.45 | 612.58 | 494.9 | 418.76 | 381.1 |
| **FEZ1** | NM_005103.4:426 | Endogenous | 709.71 | 249.35 | 612.69 | 79.47 | 582.18 | 235.37 |
| **FLT3** | NM_004119.1:580 | Endogenous | 269.08 | 129.76 | 309.6 | 100.28 | 252.75 | 119.57 |
| **FLT3LG** | NM_001459.3:360 | Endogenous | 1029.91 | 426.11 | 1393.23 | 386.4 | 1434.46 | 596.85 |
| **FN1** | NM_212482.1:1776 | Endogenous | 1874.32 | 940.34 | 1491.93 | 351 | 1227.42 | 1380.72 |
| **FOS** | NM_005252.2:1475 | Endogenous | 4194.67 | 12232.38 | 17016.91 | 16036.3 | 10623.21 | 40435.88 |
| **FOXJ1** | NM_001454.3:815 | Endogenous | 47.9 | 39.53 | 33.87 | 10.39 | 42.46 | 37.86 |
| **FOXP3** | NM_014009.3:1230 | Endogenous | 297.5 | 171.76 | 413.4 | 176.72 | 282.66 | 162.71 |
| **FPR2** | NM_001462.3:1200 | Endogenous | 115.11 | 116.55 | 72.53 | 44.64 | 67.59 | 45.37 |
| **FUT5** | NM_002034.2:1726 | Endogenous | 59.53 | 212.6 | 16.41 | 39.5 | 12.4 | 7.76 |
| **FUT7** | NM_004479.3:1710 | Endogenous | 92.72 | 156.92 | 114.44 | 70.42 | 61.99 | 33.65 |
| **FYN** | NM_002037.3:765 | Endogenous | 2530.39 | 805.28 | 4112.28 | 934.67 | 4190.69 | 1385.65 |
| **G6PD** | NM_000402.2:1155 | Housekeeping | 773.67 | 146.38 | 1105.38 | 195.17 | 1156.48 | 483.83 |
| **GAGE1** | NM_001040663.2:1642 | Endogenous | 59.49 | 78.06 | 25.57 | 27.41 | 26.17 | 21.08 |
| **GATA3** | NM_001002295.1:2835 | Endogenous | 66.02 | 77.85 | 66.15 | 16.54 | 54.41 | 21.12 |
| **GNLY** | NM_006433.2:305 | Endogenous | 302.69 | 123.74 | 325.16 | 330.07 | 227.99 | 175.23 |
| **GPATCH3** | NM_022078.2:1685 | Housekeeping | 282.17 | 138.43 | 364.02 | 46.02 | 380.49 | 104.83 |
| **GPI** | NM_000175.2:1695 | Endogenous | 2857.64 | 753.12 | 2894.6 | 360.14 | 3004.24 | 719.97 |
| **GTF3C1** | NM_001520.3:2904 | Endogenous | 1576.13 | 256.19 | 1763.39 | 109.95 | 1815.49 | 207.93 |
| **GUSB** | NM_000181.1:1350 | Housekeeping | 801.98 | 241.64 | 1000.17 | 203.93 | 1180.76 | 263.8 |
| **GZMA** | NM_006144.2:155 | Endogenous | 1279.64 | 557.04 | 1043.29 | 629.63 | 1014.21 | 385.32 |
| **GZMB** | NM_004131.3:540 | Endogenous | 452.93 | 285.75 | 370.63 | 126.98 | 194.99 | 126.3 |
| **GZMH** | NM_033423.3:705 | Endogenous | 172.53 | 90.38 | 121.56 | 67.6 | 104.59 | 34.65 |
| **GZMK** | NM_002104.2:700 | Endogenous | 1463.54 | 811.79 | 1527.77 | 338.06 | 1193.15 | 476.65 |
| **GZMM** | NM_005317.2:669 | Endogenous | 230.47 | 173.02 | 435.98 | 152.71 | 365.66 | 184.54 |
| **HAMP** | NM_021175.2:99 | Endogenous | 37.4 | 16.1 | 47.61 | 20.54 | 28.56 | 12.48 |
| **HAVCR2** | NM_032782.3:955 | Endogenous | 462.99 | 163.15 | 358.92 | 43.97 | 280.38 | 86.79 |
| **HCK** | NM_002110.2:260 | Endogenous | 1283.89 | 339.21 | 1764.97 | 513.35 | 1542.03 | 646.93 |
| **HDAC3** | NM_003883.2:1455 | Housekeeping | 1321.87 | 154.57 | 1502.54 | 158.97 | 1338.66 | 159.89 |
| **HLA-A** | NM_002116.5:1000 | Endogenous | 28031.12 | 9137.73 | 31326.38 | 4877.82 | 27227.61 | 5325.59 |
| **HLA-B** | NM_005514.6:937 | Endogenous | 28146.42 | 12357.75 | 30280.34 | 6528 | 27609.7 | 6330.41 |
| **HLA-C** | NM_002117.4:895 | Endogenous | 11173.87 | 4531.97 | 17417.43 | 5111.51 | 16452.04 | 5724.63 |
| **HLA-DMA** | NM_006120.3:380 | Endogenous | 3689.92 | 1315.97 | 5369.29 | 794.92 | 4006.63 | 1592.53 |
| **HLA-DMB** | NM_002118.3:20 | Endogenous | 3664.91 | 1726.02 | 3659.56 | 797.86 | 2461.46 | 932.42 |
| **HLA-DOB** | NM_002120.3:230 | Endogenous | 1516.8 | 845.97 | 2391.42 | 789.32 | 1612.18 | 857.48 |
| **HLA-DPA1** | NM_033554.2:857 | Endogenous | 10091.3 | 4742.52 | 10486.92 | 2471.19 | 7132.67 | 3056.6 |
| **HLA-DPB1** | NM_002121.4:931 | Endogenous | 16552.1 | 6558.64 | 14446.85 | 3703.62 | 9805.99 | 4427.93 |
| **HLA-DQA1** | NM_002122.3:261 | Endogenous | 2730.26 | 14929.25 | 15918.24 | 7024.46 | 298.74 | 12466.84 |
| **HLA-DQB1** | NM_002123.3:384 | Endogenous | 632.54 | 2853.24 | 4086.62 | 3400.34 | 192.94 | 3348.22 |
| **HLA-DRA** | NM_019111.3:335 | Endogenous | 27511.97 | 12364.9 | 25910.09 | 6019.26 | 18018.88 | 7847.14 |
| **HLA-DRB3** | NM_022555.3:698 | Endogenous | 16798.16 | 7498.7 | 38203.92 | 14893.34 | 31121.29 | 36736.41 |
| **HLA-DRB4** | NM_021983.4:194 | Endogenous | 98.57 | 1923.6 | 31.31 | 1149.86 | 206.69 | 3127 |
| **HLA-E** | NM_005516.4:1204 | Endogenous | 17140.84 | 4136 | 26217.6 | 3881.57 | 23815.09 | 3799.09 |
| **HLA-G** | NM_002127.4:1180 | Endogenous | 4446.11 | 2181.21 | 1883.77 | 672.34 | 1443.07 | 574.13 |
| **HMGB1** | NM_002128.4:208 | Endogenous | 3465.68 | 1444.99 | 4335.87 | 638.84 | 3484.56 | 705.83 |
| **HPRT1** | NM_000194.1:240 | Housekeeping | 2231.15 | 381.06 | 1742.01 | 217.67 | 1660.74 | 174.67 |
| **HRAS** | NM_005343.2:396 | Endogenous | 222.78 | 54.67 | 208.97 | 15.83 | 222.92 | 43.94 |
| **HSD11B1** | NM_181755.1:155 | Endogenous | 52.16 | 22.17 | 77.7 | 23.78 | 53.39 | 36.41 |
| **ICAM1** | NM_000201.2:2253 | Endogenous | 881.38 | 350.32 | 1450.1 | 561.84 | 1102.97 | 420.41 |
| **ICAM2** | NM_000873.3:415 | Endogenous | 1218.5 | 280.54 | 1152.79 | 192.97 | 1013.82 | 192.73 |
| **ICAM3** | NM_002162.3:1225 | Endogenous | 3621.52 | 1056.35 | 4522.94 | 675.82 | 4164.22 | 1259.9 |
| **ICAM4** | NM_001039132.1:463 | Endogenous | 111.2 | 175.9 | 78.6 | 67.23 | 71.79 | 38.3 |
| **ICOS** | NM_012092.2:640 | Endogenous | 498.03 | 299.8 | 492.26 | 133.73 | 330.75 | 161.7 |
| **ICOSLG** | NM_015259.4:1190 | Endogenous | 652.99 | 214.87 | 954.03 | 425.47 | 720.64 | 203.03 |
| **IDO1** | NM_002164.3:50 | Endogenous | 919.14 | 639.12 | 1193.39 | 558.44 | 732.26 | 769.06 |
| **IFI16** | NM_005531.1:2255 | Endogenous | 2939.88 | 1196.5 | 4628.92 | 650.54 | 4314.6 | 2130.41 |
| **IFI27** | NM_005532.3:390 | Endogenous | 7843.73 | 4947.64 | 7542.26 | 4189.61 | 11686.61 | 14442.08 |
| **IFI35** | NM_005533.3:415 | Endogenous | 740.37 | 228 | 910.28 | 254.97 | 1037.19 | 306.52 |
| **IFIH1** | NM_022168.2:185 | Endogenous | 787.48 | 120.81 | 686.71 | 138.14 | 742.71 | 195.46 |
| **IFIT1** | NM_001548.3:1440 | Endogenous | 339.59 | 126.31 | 388.97 | 98.15 | 469.78 | 359.36 |
| **IFIT2** | NM_001547.4:1995 | Endogenous | 413.95 | 268.51 | 289.19 | 127.21 | 303.41 | 120.08 |
| **IFITM1** | NM_003641.3:482 | Endogenous | 9081.76 | 3012.44 | 5983.18 | 1422.88 | 6679.28 | 2404.25 |
| **IFITM2** | NM_006435.2:390 | Endogenous | 5404.22 | 1127.12 | 5328.28 | 1759.23 | 5512.26 | 1673.42 |
| **IFNA1** | NM_024013.1:585 | Endogenous | 69.99 | 178.64 | 24.46 | 52.66 | 15.66 | 5.22 |
| **IFNA17** | NM_021268.2:291 | Endogenous | 132.88 | 518.02 | 32.2 | 163.74 | 19.65 | 7.9 |
| **IFNA2** | NM_000605.3:611 | Endogenous | 79.72 | 193.7 | 23.73 | 69.12 | 16.81 | 6.79 |
| **IFNA7** | NM_021057.2:215 | Endogenous | 220.56 | 1096.17 | 37.4 | 255.48 | 25.19 | 16.76 |
| **IFNA8** | NM_002170.3:604 | Endogenous | 63.22 | 207.74 | 16.22 | 60.95 | 11.64 | 6.05 |
| **IFNAR1** | NM_000629.2:3123 | Endogenous | 761.38 | 254.79 | 810.8 | 84.82 | 815.43 | 103.09 |
| **IFNAR2** | NM_000874.3:631 | Endogenous | 3054.12 | 656.82 | 3512.62 | 464.18 | 3456.08 | 742.58 |
| **IFNB1** | NM_002176.2:610 | Endogenous | 65.69 | 204.28 | 21.78 | 56.87 | 12.01 | 5.16 |
| **IFNG** | NM_000619.2:970 | Endogenous | 118.03 | 142.49 | 73.84 | 68.26 | 47.09 | 16.8 |
| **IFNGR1** | NM_000416.1:1140 | Endogenous | 3926.47 | 1008.76 | 3114.9 | 552.69 | 2597.21 | 630.57 |
| **IFNL1** | NM_172140.1:233 | Endogenous | 47.89 | 15.72 | 38.62 | 13.58 | 36.1 | 12.35 |
| **IFNL2** | NM_172138.1:589 | Endogenous | 128.56 | 378.86 | 38.32 | 97.31 | 18.25 | 5.43 |
| **IGF1R** | NM_000875.2:455 | Endogenous | 1834.29 | 317.51 | 1905.52 | 316.85 | 2571.01 | 1202.25 |
| **IGF2R** | NM_000876.1:2605 | Endogenous | 1399.42 | 303.41 | 1448.32 | 183.1 | 1583.38 | 225.73 |
| **IGLL1** | NM_020070.2:188 | Endogenous | 31.97 | 70.97 | 19.92 | 13.28 | 15.56 | 5.25 |
| **IKBKB** | NM_001556.1:1995 | Endogenous | 869.36 | 271.81 | 1261.37 | 264.09 | 1395.78 | 486.83 |
| **IKBKE** | NM_014002.2:2470 | Endogenous | 1361.28 | 309 | 1945.87 | 503.26 | 1597.39 | 470.29 |
| **IKBKG** | NM_003639.2:470 | Endogenous | 705.81 | 110.24 | 811.79 | 166.39 | 854.93 | 165.18 |
| **IL10** | NM_000572.2:230 | Endogenous | 63.86 | 18.19 | 94.39 | 26.42 | 71.77 | 26.02 |
| **IL10RA** | NM_001558.2:150 | Endogenous | 1729.68 | 642.56 | 2173.8 | 559.08 | 1957.42 | 574.01 |
| **IL11** | NM_000641.2:1145 | Endogenous | 44.33 | 52.08 | 34.93 | 35.66 | 32.6 | 19.13 |
| **IL11RA** | NM_147162.1:400 | Endogenous | 453.63 | 191.41 | 498.91 | 94.92 | 539.59 | 137.1 |
| **IL12A** | NM_000882.2:775 | Endogenous | 59.56 | 16.3 | 69.79 | 12.95 | 73.53 | 13.73 |
| **IL12B** | NM_002187.2:1435 | Endogenous | 86.81 | 145.54 | 42.43 | 54.51 | 21.6 | 7.99 |
| **IL12RB1** | NM_005535.1:225 | Endogenous | 377.17 | 243.77 | 535.25 | 165.44 | 383.49 | 132.54 |
| **IL12RB2** | NM_001559.2:1315 | Endogenous | 127.72 | 71.92 | 91.16 | 43.76 | 79.28 | 22.74 |
| **IL13** | NM_002188.2:516 | Endogenous | 77.6 | 149.53 | 30.64 | 38.55 | 22.3 | 6.18 |
| **IL13RA1** | NM_001560.2:1230 | Endogenous | 2992.19 | 907.07 | 3310.26 | 699.49 | 3793.04 | 615.21 |
| **IL13RA2** | NM_000640.2:400 | Endogenous | 59.73 | 62.33 | 45.01 | 32.84 | 38.5 | 18.81 |
| **IL15** | NM_172174.1:1685 | Endogenous | 413.75 | 165.98 | 370.7 | 60.82 | 336.78 | 44.83 |
| **IL15RA** | NM_002189.2:505 | Endogenous | 864.18 | 188.5 | 898.21 | 149.85 | 825.6 | 131.06 |
| **IL16** | NM_004513.4:1262 | Endogenous | 5049.5 | 2332.91 | 6648.94 | 1804.83 | 6010.25 | 2778.97 |
| **IL17A** | NM_002190.2:240 | Endogenous | 36.8 | 28.51 | 31.14 | 79.41 | 23.28 | 20.78 |
| **IL17B** | NM_014443.2:177 | Endogenous | 44.67 | 26.56 | 42.11 | 16.1 | 28.89 | 14.78 |
| **IL17F** | NM_052872.3:210 | Endogenous | 122.28 | 224.63 | 43.59 | 62.23 | 27.41 | 6.55 |
| **IL17RA** | NM_014339.6:482 | Endogenous | 942.13 | 162.66 | 907.67 | 127.58 | 895.2 | 149.8 |
| **IL17RB** | NM_018725.3:225 | Endogenous | 273.67 | 129.11 | 324.76 | 194.14 | 399.03 | 212.2 |
| **IL18** | NM_001562.2:48 | Endogenous | 1297.72 | 728.31 | 692 | 192.93 | 664.61 | 274.69 |
| **IL18R1** | NM_003855.2:2025 | Endogenous | 813.21 | 205.09 | 795.53 | 114.96 | 807.78 | 240.25 |
| **IL18RAP** | NM_003853.2:2412 | Endogenous | 305.79 | 154 | 314.17 | 136.36 | 328.77 | 109.46 |
| **IL19** | NM_013371.3:1030 | Endogenous | 17.55 | 11.81 | 13.5 | 5.03 | 10.54 | 4.15 |
| **IL1A** | NM_000575.3:1085 | Endogenous | 91.84 | 188.36 | 45.69 | 58.97 | 36.49 | 15.76 |
| **IL1B** | NM_000576.2:840 | Endogenous | 324.14 | 215.4 | 437.6 | 361.36 | 403.23 | 427.4 |
| **IL1R1** | NM_000877.2:4295 | Endogenous | 994.46 | 304.37 | 1414.74 | 393.06 | 1313.38 | 320.69 |
| **IL1R2** | NM_173343.1:113 | Endogenous | 521.58 | 487.07 | 470.46 | 208.22 | 574.95 | 177.25 |
| **IL1RAP** | NM_002182.2:460 | Endogenous | 363.03 | 85.79 | 301.51 | 38.84 | 342.42 | 78.65 |
| **IL1RAPL2** | NM_017416.1:1800 | Endogenous | 117.04 | 166.42 | 66.46 | 26.84 | 51.39 | 15.51 |
| **IL1RL1** | NM_016232.4:700 | Endogenous | 445.03 | 208.1 | 326.65 | 112.49 | 252.61 | 187.55 |
| **IL1RL2** | NM_003854.2:595 | Endogenous | 78.54 | 51.79 | 43.38 | 31.06 | 48.68 | 13.6 |
| **IL1RN** | NM_000577.3:480 | Endogenous | 146.79 | 197.82 | 98.56 | 74.28 | 83.13 | 53.47 |
| **IL2** | NM_000586.2:300 | Endogenous | 84.98 | 145.41 | 31.84 | 65.59 | 21 | 9.67 |
| **IL21** | NM_021803.2:65 | Endogenous | 202.86 | 176.6 | 164.55 | 97.37 | 68.64 | 61.97 |
| **IL21R** | NM_021798.2:2080 | Endogenous | 542.7 | 473 | 906.02 | 486.74 | 493.46 | 295.25 |
| **IL22** | NM_020525.4:319 | Endogenous | 61.37 | 117.83 | 21.99 | 43.95 | 16.22 | 24.47 |
| **IL22RA1** | NM_021258.2:2524 | Endogenous | 408.08 | 723.83 | 162.39 | 134.27 | 246.54 | 154.21 |
| **IL22RA2** | NM_181310.1:290 | Endogenous | 178.03 | 200.91 | 207.42 | 90.63 | 110.54 | 105.77 |
| **IL23A** | NM_016584.2:411 | Endogenous | 116.64 | 106.01 | 97.18 | 64.82 | 75.52 | 34.07 |
| **IL23R** | NM_144701.2:710 | Endogenous | 234.29 | 137.12 | 107.08 | 42.56 | 110.89 | 33.87 |
| **IL24** | NM_181339.1:1016 | Endogenous | 72.11 | 146.23 | 23.33 | 76.13 | 16.9 | 8.53 |
| **IL25** | NM_022789.2:1027 | Endogenous | 61.6 | 115.96 | 24.62 | 25.93 | 14.21 | 4.85 |
| **IL26** | NM_018402.1:80 | Endogenous | 125 | 169.68 | 48.13 | 67.18 | 30.83 | 15.9 |
| **IL27** | NM_145659.3:143 | Endogenous | 60.43 | 111.2 | 28.5 | 33.87 | 17.36 | 7.45 |
| **IL2RA** | NM_000417.1:1000 | Endogenous | 840.38 | 358.77 | 1061.03 | 384.35 | 658.8 | 432.91 |
| **IL2RB** | NM_000878.2:1980 | Endogenous | 828.55 | 390.11 | 1975.69 | 550.57 | 1445.77 | 558.02 |
| **IL2RG** | NM_000206.1:595 | Endogenous | 4455.25 | 1369.98 | 4878.56 | 824.68 | 4658.39 | 1115.86 |
| **IL3** | NM_000588.3:130 | Endogenous | 44.56 | 94.08 | 19.34 | 27.28 | 10.64 | 4.2 |
| **IL32** | NM_004221.4:358 | Endogenous | 4552.38 | 1917.1 | 5607.49 | 1516.15 | 5695.65 | 2124.23 |
| **IL34** | NM_152456.1:860 | Endogenous | 238.58 | 65.55 | 375.76 | 70.68 | 324.57 | 87.85 |
| **IL3RA** | NM_002183.2:745 | Endogenous | 184.11 | 95.96 | 157.72 | 42.95 | 134.86 | 53.84 |
| **IL4** | NM_000589.2:625 | Endogenous | 56.2 | 101.52 | 32.17 | 28.58 | 21.31 | 10.41 |
| **IL4R** | NM_000418.2:705 | Endogenous | 1798.42 | 443.51 | 2312.05 | 539.82 | 2020.95 | 674.28 |
| **IL5** | NM_000879.2:105 | Endogenous | 74.65 | 136.29 | 44.14 | 43.6 | 26.27 | 8.46 |
| **IL5RA** | NM_000564.3:210 | Endogenous | 106.95 | 128.44 | 42.37 | 32.4 | 37.63 | 8.46 |
| **IL6** | NM_000600.1:220 | Endogenous | 66.48 | 39.24 | 126.66 | 302.83 | 80.65 | 39.98 |
| **IL6R** | NM_000565.2:993 | Endogenous | 691.68 | 477.28 | 1138.89 | 103.5 | 1102.37 | 201.31 |
| **IL6ST** | NM_002184.2:2505 | Endogenous | 10600.63 | 3311.66 | 8030.1 | 1541.12 | 7126.38 | 2234.71 |
| **IL7** | NM_000880.2:38 | Endogenous | 400.45 | 191.15 | 221.42 | 98.82 | 193.81 | 50.37 |
| **IL7R** | NM_002185.2:1610 | Endogenous | 5255.19 | 3586.78 | 4568.86 | 1355.49 | 4530.11 | 2534.7 |
| **IL8** | NM_000584.2:25 | Endogenous | 296.36 | 515.59 | 433.78 | 1057.54 | 314.8 | 990.83 |
| **IL9** | NM_000590.1:300 | Endogenous | 27.99 | 30.15 | 13.27 | 5.84 | 9.9 | 3.09 |
| **ILF3** | NM_001137673.1:730 | Endogenous | 5914.3 | 694.46 | 6268.28 | 575.75 | 6184.63 | 847 |
| **INPP5D** | NM_005541.3:4075 | Endogenous | 2918.98 | 1247.1 | 3399.2 | 872.94 | 2601.21 | 818.4 |
| **IRAK1** | NM_001569.3:1995 | Endogenous | 1506.32 | 295.3 | 1604.76 | 122.7 | 1690.01 | 216.72 |
| **IRAK2** | NM_001570.3:1285 | Endogenous | 305.34 | 131.85 | 330.38 | 87.6 | 315.97 | 105.47 |
| **IRAK4** | NM_016123.1:2175 | Endogenous | 998.2 | 190.19 | 1495.31 | 254 | 1341.79 | 227.92 |
| **IRF1** | NM_002198.1:510 | Endogenous | 1123.6 | 619.81 | 1727.76 | 623.35 | 1619.65 | 711.64 |
| **IRF2** | NM_002199.3:1624 | Endogenous | 2288.85 | 390.47 | 2138.06 | 352.56 | 1824.7 | 264.36 |
| **IRF3** | NM_001571.5:1303 | Endogenous | 550.02 | 119.03 | 1269.1 | 183.74 | 1326.56 | 517.18 |
| **IRF4** | NM_002460.1:325 | Endogenous | 2877.5 | 1038.88 | 3698.67 | 1392.26 | 3823.99 | 1310.17 |
| **IRF5** | NM_002200.3:1845 | Endogenous | 345.88 | 94.11 | 667.01 | 117.67 | 602.68 | 217.76 |
| **IRF7** | NM_001572.3:1763 | Endogenous | 931.3 | 266.74 | 1233.64 | 233.71 | 1172.66 | 525.87 |
| **IRF8** | NM_002163.2:253 | Endogenous | 2384.46 | 1087.99 | 4058.91 | 922.18 | 2937.53 | 1526.19 |
| **IRGM** | NM_001145805.1:1412 | Endogenous | 87.38 | 117.67 | 54.89 | 35.36 | 42.34 | 18.64 |
| **ISG15** | NM_005101.3:305 | Endogenous | 744.7 | 233.37 | 965.21 | 152.36 | 1145.8 | 961.1 |
| **ISG20** | NM_002201.4:358 | Endogenous | 1944.39 | 737.1 | 2330.89 | 487.96 | 1931.8 | 825.5 |
| **ITCH** | NM_001257138.1:438 | Endogenous | 3412.88 | 690.72 | 2370.06 | 384 | 2572.35 | 414.65 |
| **ITGA1** | NM_181501.1:1875 | Endogenous | 2171.5 | 428.11 | 2192.74 | 469.57 | 2198.63 | 1440.23 |
| **ITGA2** | NM_002203.2:475 | Endogenous | 649.12 | 1032.46 | 343.92 | 149.02 | 423.54 | 108.76 |
| **ITGA2B** | NM_000419.3:740 | Endogenous | 81.55 | 190.17 | 44.87 | 45 | 24.2 | 16.05 |
| **ITGA4** | NM_000885.4:975 | Endogenous | 1741.99 | 614.73 | 2694.81 | 519.71 | 2355.77 | 804.65 |
| **ITGA5** | NM_002205.2:925 | Endogenous | 1187.59 | 605.8 | 1177.56 | 371.69 | 1146 | 805.17 |
| **ITGA6** | NM_000210.1:3065 | Endogenous | 4914.98 | 2786.43 | 2667.45 | 907.54 | 3469.91 | 1067.7 |
| **ITGAE** | NM_002208.4:3405 | Endogenous | 852.02 | 162.74 | 735.76 | 80.91 | 629.96 | 128.91 |
| **ITGAL** | NM_002209.2:3905 | Endogenous | 1600.04 | 829.71 | 2715.08 | 651.65 | 2170.91 | 1151.99 |
| **ITGAM** | NM_000632.3:515 | Endogenous | 489.08 | 132.87 | 530.78 | 129.61 | 454.29 | 134.97 |
| **ITGAX** | NM_000887.3:700 | Endogenous | 1271.58 | 548.26 | 1478.37 | 495.39 | 835.19 | 585.3 |
| **ITGB1** | NM_033666.2:2000 | Endogenous | 9826.45 | 3295.29 | 8092.2 | 1816.91 | 8446.82 | 2963.29 |
| **ITGB2** | NM_000211.2:520 | Endogenous | 1921.09 | 769.5 | 3212 | 784.92 | 2773.72 | 1278.89 |
| **ITGB3** | NM_000212.2:4485 | Endogenous | 224.08 | 79 | 273.53 | 89.89 | 299.1 | 94.61 |
| **ITGB4** | NM_001005731.1:4151 | Endogenous | 3018.2 | 2736.66 | 3134.28 | 922.3 | 4376.52 | 1218.53 |
| **ITK** | NM_005546.3:3430 | Endogenous | 1513.84 | 985.07 | 1935.67 | 779.15 | 1567.66 | 938.46 |
| **JAK1** | NM_002227.1:285 | Endogenous | 4476.78 | 725.97 | 5070.72 | 504.27 | 4654.32 | 941.61 |
| **JAK2** | NM_004972.2:455 | Endogenous | 2849.81 | 435.76 | 2949.57 | 487.46 | 2981.61 | 688.55 |
| **JAK3** | NM_000215.2:1715 | Endogenous | 1731.26 | 859.41 | 3309.65 | 1322.87 | 2508.78 | 1655.93 |
| **JAM3** | NM_032801.3:890 | Endogenous | 924.78 | 209.44 | 1068.08 | 136.99 | 1089.64 | 399.09 |
| **KIR_Activating_Subgroup_1** | NM_001083539.1:1146 | Endogenous | 14.37 | 3.47 | 10.78 | 1.04 | 10.51 | 4.37 |
| **KIR_Activating_Subgroup_2** | NM_014512.1:718 | Endogenous | 76.31 | 222.32 | 22.51 | 37.75 | 16.81 | 5.28 |
| **KIR_Inhibiting_Subgroup_1** | NM_014218.2:872 | Endogenous | 37.04 | 11.57 | 36.68 | 13.18 | 38.97 | 16.44 |
| **KIR_Inhibiting_Subgroup_2** | NM_014511.3:592 | Endogenous | 35.71 | 16.91 | 34.64 | 27.2 | 37.65 | 17.68 |
| **KIR3DL1** | NM_013289.2:1691 | Endogenous | 84.26 | 143.3 | 29.9 | 14.02 | 22.9 | 7.79 |
| **KIR3DL2** | NM_006737.2:884 | Endogenous | 87.96 | 244.46 | 31.35 | 48.73 | 27.29 | 7.85 |
| **KIR3DL3** | NM_153443.3:539 | Endogenous | 59.71 | 160.56 | 19.94 | 23.45 | 13.95 | 4.59 |
| **KIT** | NM_000222.2:2644 | Endogenous | 594.24 | 186.49 | 550.83 | 232.64 | 600.66 | 269.67 |
| **KLRB1** | NM_002258.2:85 | Endogenous | 2547.44 | 1520.34 | 2491.17 | 346.11 | 2103.09 | 711.5 |
| **KLRC1** | NM_002259.3:335 | Endogenous | 227.55 | 158.55 | 117 | 46.96 | 160.53 | 51.59 |
| **KLRC2** | NM_002260.3:942 | Endogenous | 272.55 | 169.4 | 263.91 | 178.19 | 290.73 | 244.18 |
| **KLRD1** | NM_002262.3:542 | Endogenous | 194 | 93.65 | 126.2 | 63.35 | 125.78 | 38.97 |
| **KLRF1** | NM_016523.1:275 | Endogenous | 168.9 | 87.56 | 124.72 | 60.02 | 98.61 | 34.1 |
| **KLRG1** | NM_005810.3:65 | Endogenous | 341.85 | 267.82 | 333.98 | 159.79 | 277.3 | 170.97 |
| **KLRK1** | NM_007360.3:522 | Endogenous | 758.2 | 297.73 | 566.05 | 152.94 | 646.98 | 235.89 |
| **LAG3** | NM_002286.5:1735 | Endogenous | 720.58 | 357.74 | 1159.26 | 423.54 | 849.16 | 319.86 |
| **LAIR2** | NM_002288.3:524 | Endogenous | 787.2 | 300.06 | 1078.08 | 442.66 | 955.35 | 465.44 |
| **LAMP1** | NM_005561.3:2070 | Endogenous | 7530.65 | 2483.62 | 8370.72 | 1113.68 | 8458.35 | 1064.18 |
| **LAMP2** | NM_001122606.1:46 | Endogenous | 2409.87 | 470.8 | 3084.15 | 624.78 | 3153.03 | 447.02 |
| **LAMP3** | NM_014398.3:1400 | Endogenous | 604.93 | 319.72 | 745.87 | 377.03 | 480.3 | 389.44 |
| **LBP** | NM_004139.2:792 | Endogenous | 60.63 | 121.42 | 20.74 | 39.48 | 15.89 | 27.1 |
| **LCK** | NM_005356.2:1260 | Endogenous | 1645.91 | 880.17 | 2674.19 | 819.48 | 2143.42 | 1014.23 |
| **LCN2** | NM_005564.3:325 | Endogenous | 2264.96 | 3323.12 | 2193.27 | 7188.37 | 1219.37 | 2167.17 |
| **LCP1** | NM_002298.4:3195 | Endogenous | 8666.59 | 4374.97 | 15099.78 | 3906.88 | 9408.17 | 3907.35 |
| **LGALS3** | NM_001177388.1:495 | Endogenous | 19691.36 | 18139.79 | 11847.52 | 8745.16 | 19158.46 | 6892.98 |
| **LIF** | NM_002309.3:1240 | Endogenous | 278.33 | 131.6 | 326.84 | 117.87 | 269.12 | 116.85 |
| **LILRA1** | NM_006863.1:1719 | Endogenous | 90.49 | 67.32 | 85.77 | 29.9 | 68.94 | 66.26 |
| **LILRA4** | NM_012276.3:1577 | Endogenous | 67.88 | 40.56 | 134.45 | 66.14 | 73.52 | 40.24 |
| **LILRA5** | NM_181879.2:545 | Endogenous | 119.07 | 138.32 | 66.56 | 45.36 | 84.48 | 198.4 |
| **LILRB1** | NM_001081637.1:2332 | Endogenous | 863.97 | 283.46 | 1000.58 | 196.3 | 813.16 | 400.3 |
| **LILRB2** | NM_005874.1:595 | Endogenous | 383.1 | 201.82 | 498.54 | 152.06 | 512.93 | 740.92 |
| **LILRB3** | NM_006864.2:2235 | Endogenous | 141.87 | 90.05 | 134.89 | 70.25 | 139.12 | 129.5 |
| **LRP1** | NM_002332.2:4240 | Endogenous | 2589.71 | 673.62 | 2494.99 | 439.25 | 2918.77 | 976.02 |
| **LRRN3** | NM_001099660.1:2940 | Endogenous | 254.55 | 273.76 | 195.82 | 77 | 325.1 | 250.25 |
| **LTA** | NM_000595.2:885 | Endogenous | 421.58 | 266.11 | 525.86 | 288.36 | 305.92 | 254.35 |
| **LTB** | NM_002341.1:330 | Endogenous | 2488.67 | 2136.55 | 6214.99 | 2891.2 | 4429.15 | 5723.3 |
| **LTBR** | NM_002342.1:1435 | Endogenous | 1653.33 | 784.25 | 1740.97 | 365.02 | 2481.49 | 696.84 |
| **LTF** | NM_002343.2:590 | Endogenous | 966.63 | 776.55 | 1846.45 | 1290.23 | 1010.97 | 1201.91 |
| **LTK** | NM_001135685.1:2418 | Endogenous | 119.74 | 73.52 | 191.61 | 71.78 | 241.75 | 131.77 |
| **LY86** | NM_004271.3:255 | Endogenous | 866.7 | 445.82 | 903.97 | 134.36 | 698.51 | 427.23 |
| **LY9** | NM_001033667.1:260 | Endogenous | 1954.91 | 768.82 | 1689.24 | 218.88 | 1493.79 | 589.28 |
| **LY96** | NM_015364.2:360 | Endogenous | 909.68 | 280.99 | 1054.95 | 293.44 | 957.31 | 333.86 |
| **LYN** | NM_002350.1:1285 | Endogenous | 845.14 | 331.94 | 1519.5 | 352.21 | 1184.35 | 447.89 |
| **MAF** | NM_005360.4:888 | Endogenous | 1304 | 366.53 | 1890.65 | 346.54 | 1809.37 | 405.81 |
| **MAGEA1** | NM_004988.4:476 | Endogenous | 33.01 | 53.53 | 14.89 | 12.75 | 12.42 | 3.95 |
| **MAGEA12** | NM_001166386.1:567 | Endogenous | 53.07 | 65.96 | 23.32 | 17.41 | 15.97 | 5.84 |
| **MAGEA3** | NM_005362.3:849 | Endogenous | 63.12 | 92.47 | 27.69 | 28.64 | 17.19 | 7.61 |
| **MAGEA4** | NM_001011548.1:778 | Endogenous | 40.13 | 44.84 | 31.66 | 10.14 | 19.47 | 9.12 |
| **MAGEB2** | NM_002364.4:1026 | Endogenous | 86.25 | 135.26 | 30.88 | 27.68 | 19.36 | 8.88 |
| **MAGEC1** | NM_005462.4:2920 | Endogenous | 32.29 | 50.46 | 17.1 | 10.29 | 12.14 | 5.27 |
| **MAGEC2** | NM_016249.3:860 | Endogenous | 49.04 | 134.34 | 18.55 | 22.83 | 14.49 | 5.51 |
| **MAP2K1** | NM_002755.2:970 | Endogenous | 1637.98 | 232.72 | 1544.97 | 189.59 | 1353.46 | 198.26 |
| **MAP2K2** | NM_030662.2:1325 | Endogenous | 4383.59 | 1109.33 | 5107.64 | 597.97 | 6077.53 | 2158.97 |
| **MAP2K4** | NM_003010.2:2830 | Endogenous | 868.19 | 179.25 | 893.16 | 125.03 | 820.59 | 147.5 |
| **MAP3K1** | NM_005921.1:2525 | Endogenous | 1786.23 | 590.66 | 1912.75 | 458.61 | 1710.54 | 338.23 |
| **MAP3K5** | NM_005923.3:1760 | Endogenous | 2822.18 | 477.52 | 2418.72 | 157.23 | 2417.84 | 515.06 |
| **MAP3K7** | NM_145333.1:670 | Endogenous | 2302.52 | 276.79 | 2169.19 | 236.59 | 2083.95 | 246.98 |
| **MAP4K2** | NM_004579.2:1610 | Endogenous | 882.86 | 289.2 | 1275.89 | 294.54 | 1190.9 | 450.82 |
| **MAPK1** | NM_138957.2:430 | Endogenous | 3403.18 | 455.87 | 2216.31 | 275.89 | 2334.19 | 422.18 |
| **MAPK11** | NM_002751.5:1310 | Endogenous | 118.41 | 108.98 | 204.81 | 71.79 | 174.86 | 66.75 |
| **MAPK14** | NM_001315.1:450 | Endogenous | 3054.15 | 340.53 | 3098.98 | 308.6 | 3477.61 | 397.86 |
| **MAPK3** | NM_001040056.1:580 | Endogenous | 3213.85 | 1667.7 | 2489.32 | 639.23 | 3383.94 | 608.43 |
| **MAPK8** | NM_002750.2:945 | Endogenous | 810.94 | 216.79 | 667.5 | 93.56 | 698.08 | 89.64 |
| **MAPKAPK2** | NM_004759.3:710 | Endogenous | 1794.17 | 471.34 | 2436.63 | 209.95 | 2356.62 | 310.05 |
| **MARCO** | NM_006770.3:1434 | Endogenous | 98.94 | 169.76 | 78.71 | 154.24 | 101.08 | 324.93 |
| **MASP1** | NM_139125.3:35 | Endogenous | 61.38 | 67.72 | 42.05 | 22.45 | 30.53 | 9.69 |
| **MASP2** | NM_139208.1:330 | Endogenous | 46.07 | 93.84 | 18.42 | 17.65 | 12.76 | 6 |
| **MAVS** | NM_020746.3:3460 | Endogenous | 2675.55 | 577.27 | 3261.48 | 478.96 | 3839.07 | 849.32 |
| **MBL2** | NM_000242.2:1756 | Endogenous | 27.24 | 40 | 18.45 | 15.29 | 12.66 | 3.91 |
| **MCAM** | NM_006500.2:1515 | Endogenous | 476.9 | 158.75 | 708.2 | 156.05 | 624.61 | 452.84 |
| **MEF2C** | NM_002397.3:2445 | Endogenous | 2781.3 | 994.91 | 4838.39 | 1447.33 | 3331.73 | 1068.98 |
| **MEFV** | NM_000243.2:1162 | Endogenous | 83.55 | 107.66 | 73.93 | 53.29 | 65.69 | 43 |
| **MERTK** | NM_006343.2:665 | Endogenous | 1081.86 | 378.05 | 726.23 | 207.73 | 769.39 | 273.89 |
| **MFGE8** | NM_001114614.1:328 | Endogenous | 1754.26 | 725.19 | 2544.61 | 470.1 | 2716.52 | 702.36 |
| **MICA** | NM_000247.1:550 | Endogenous | 400.61 | 122.03 | 343.54 | 58.88 | 412.27 | 135.63 |
| **MICB** | NM_005931.3:1387 | Endogenous | 630.46 | 237.95 | 971.63 | 230.21 | 703.22 | 160.39 |
| **MIF** | NM_002415.1:319 | Endogenous | 4309.63 | 1198.98 | 6099.95 | 1045.12 | 6393.18 | 1375.7 |
| **MME** | NM_000902.2:5059 | Endogenous | 227.79 | 189.94 | 257.47 | 140.54 | 117.82 | 72.86 |
| **MNX1** | NM_005515.3:1680 | Endogenous | 108.01 | 87.53 | 86.75 | 51.87 | 120.89 | 61.78 |
| **MPPED1** | NM_001044370.1:1486 | Endogenous | 102.68 | 233.49 | 38.55 | 33.09 | 30.68 | 24.15 |
| **MR1** | NM_001531.2:7695 | Endogenous | 404.97 | 121.28 | 332.44 | 94.73 | 274.39 | 76.81 |
| **MRC1** | NM_002438.2:525 | Endogenous | 1127.39 | 447.57 | 719.44 | 329.57 | 696.99 | 290.64 |
| **MRPS5** | NM_031902.3:390 | Housekeeping | 2663.18 | 273.68 | 1767.04 | 211.48 | 1885.7 | 251.8 |
| **MS4A1** | NM_152866.2:620 | Endogenous | 5232.98 | 3953.29 | 8828.01 | 3659.73 | 6059.73 | 4438.33 |
| **MS4A2** | NM_000139.3:661 | Endogenous | 390.27 | 170.86 | 411.97 | 73.97 | 382.86 | 198.84 |
| **MSR1** | NM_002445.3:326 | Endogenous | 482.92 | 193.75 | 286.29 | 112.61 | 247.63 | 99.47 |
| **MST1R** | NM_002447.1:3300 | Endogenous | 990.84 | 959.2 | 651.84 | 313.77 | 874.06 | 362.77 |
| **MTMR14** | NM_022485.3:720 | Housekeeping | 1662.1 | 310.33 | 2378.95 | 276.86 | 2223.13 | 457.61 |
| **MUC1** | NM_001018017.1:725 | Endogenous | 2036.31 | 1519.44 | 1275.07 | 1290.41 | 2037.52 | 1610.94 |
| **MX1** | NM_002462.2:1485 | Endogenous | 2355.07 | 799.76 | 3444.4 | 512.64 | 4030.15 | 3434.33 |
| **MYD88** | NM_002468.3:2145 | Endogenous | 2363.99 | 799.96 | 3005.44 | 563.23 | 2933.52 | 482.53 |
| **NCAM1** | NM_000615.5:1620 | Endogenous | 949.5 | 390.1 | 648.75 | 151.49 | 729.69 | 427.6 |
| **NCF4** | NM_000631.4:210 | Endogenous | 1063.9 | 298.6 | 1427.69 | 156.34 | 1318.52 | 500.57 |
| **NCR1** | NM_004829.5:602 | Endogenous | 90.78 | 27.07 | 79.48 | 15.38 | 67.8 | 26.65 |
| **NEFL** | NM_006158.3:3300 | Endogenous | 119.44 | 125.93 | 87.77 | 53.2 | 66.38 | 84.6 |
| **NEG_A** | ERCC_00096.1:230 | Negative | 11.68 | 3.37 | 8.66 | 2.85 | 6.5 | 3.6 |
| **NEG_B** | ERCC_00041.1:440 | Negative | 15.49 | 5.51 | 11.92 | 3.95 | 9.46 | 4.19 |
| **NEG_C** | ERCC_00019.1:140 | Negative | 21.7 | 7.6 | 22.02 | 6.29 | 18.12 | 8.69 |
| **NEG_D** | ERCC_00076.1:355 | Negative | 12.9 | 2.89 | 10.3 | 3.87 | 8.44 | 3.14 |
| **NEG_E** | ERCC_00098.1:785 | Negative | 12.67 | 4.53 | 9.62 | 3.37 | 9.78 | 1.81 |
| **NEG_F** | ERCC_00126.1:220 | Negative | 15.34 | 7.42 | 10.56 | 3.56 | 10.83 | 3.66 |
| **NEG_G** | ERCC_00144.1:15 | Negative | 8.28 | 3.76 | 6.45 | 1.82 | 6.55 | 3.32 |
| **NEG_H** | ERCC_00154.1:115 | Negative | 13.25 | 3.69 | 11.93 | 3.65 | 8.42 | 2.97 |
| **NFATC1** | NM_172389.1:1984 | Endogenous | 844.51 | 317.08 | 1080.44 | 286.19 | 799.88 | 295.06 |
| **NFATC2** | NM_012340.3:1815 | Endogenous | 2349.43 | 585.31 | 2528.24 | 507.12 | 2302.14 | 560.72 |
| **NFATC3** | NM_004555.2:2190 | Endogenous | 1895.82 | 437.89 | 1647.89 | 211.7 | 1448.21 | 272.67 |
| **NFATC4** | NM_001136022.2:2296 | Endogenous | 376.86 | 156.42 | 337.74 | 64.23 | 390.79 | 146.14 |
| **NFKB1** | NM_003998.2:1675 | Endogenous | 973.23 | 151.79 | 1042.71 | 102.61 | 944.85 | 189.11 |
| **NFKB2** | NM_002502.2:825 | Endogenous | 1399.56 | 613.1 | 2885.03 | 778.39 | 2602.6 | 1301.15 |
| **NFKBIA** | NM_020529.1:945 | Endogenous | 6532.83 | 3311.4 | 12220.16 | 3370.72 | 9766.78 | 3634.88 |
| **NLRC5** | NM_032206.4:860 | Endogenous | 1786.26 | 607.42 | 2195.38 | 248.36 | 2030.4 | 432.58 |
| **NLRP3** | NM_001079821.2:415 | Endogenous | 214.27 | 44.96 | 255.17 | 52.02 | 232.03 | 73.08 |
| **NOD1** | NM_006092.1:3285 | Endogenous | 431.32 | 196.09 | 637 | 89.86 | 630.61 | 80.35 |
| **NOD2** | NM_022162.1:4080 | Endogenous | 349.76 | 156.34 | 339.41 | 82.29 | 292.99 | 80.23 |
| **NOL7** | NM_016167.3:335 | Housekeeping | 1489.98 | 326.73 | 1052.13 | 143.09 | 1028.05 | 153.21 |
| **NOS2A** | NM_153292.1:546 | Endogenous | 803.51 | 1163.46 | 772.02 | 2011.69 | 656.02 | 1365.44 |
| **NOTCH1** | NM_017617.3:735 | Endogenous | 1165.88 | 205.17 | 1561.25 | 237.09 | 1752.95 | 511.77 |
| **NRP1** | NM_003873.5:370 | Endogenous | 2152.19 | 457.64 | 2488.7 | 513.89 | 2772.91 | 1070.56 |
| **NT5E** | NM_002526.2:1214 | Endogenous | 1194.54 | 583.24 | 1229.06 | 529.82 | 1429.1 | 585.96 |
| **NUBP1** | NM_001278506.1:304 | Housekeeping | 361.64 | 73.43 | 290.14 | 39.74 | 276.41 | 44.33 |
| **NUP107** | NM_020401.2:1002 | Endogenous | 1947.6 | 393.45 | 1506.66 | 158.15 | 1407.85 | 368.36 |
| **OAS3** | NM_006187.2:4980 | Endogenous | 794.33 | 186.8 | 864.57 | 207.13 | 903.78 | 662.32 |
| **OSM** | NM_020530.4:580 | Endogenous | 59.71 | 82.19 | 49.33 | 59.22 | 41.29 | 23.97 |
| **PASD1** | NM_173493.2:820 | Endogenous | 14.79 | 2.8 | 12.67 | 3.22 | 10.19 | 2.19 |
| **PAX5** | NM_016734.1:2288 | Endogenous | 1109.43 | 667.95 | 2903.45 | 1257.13 | 1664.52 | 1208 |
| **PBK** | NM_018492.2:1587 | Endogenous | 503.39 | 162.17 | 361.49 | 115.32 | 280.5 | 135.1 |
| **PDCD1** | NM_005018.1:175 | Endogenous | 227.73 | 136.88 | 329.16 | 107.37 | 186.88 | 99.71 |
| **PDCD1LG2** | NM_025239.3:235 | Endogenous | 288.9 | 123.02 | 263.8 | 53.81 | 192.72 | 90.88 |
| **PDGFC** | NM_016205.2:1632 | Endogenous | 599.76 | 110.36 | 609.7 | 125.24 | 556.24 | 301.89 |
| **PDGFRB** | NM_002609.3:840 | Endogenous | 2005.5 | 537.15 | 1575.47 | 459.23 | 1476.31 | 691.15 |
| **PECAM1** | NM_000442.3:1365 | Endogenous | 3581.26 | 712.85 | 3222.32 | 722.72 | 2860.75 | 861.83 |
| **PIK3CD** | NM_005026.3:2978 | Endogenous | 1451.89 | 775.1 | 2464.24 | 683.85 | 1838.99 | 785.32 |
| **PIK3CG** | NM_002649.2:2125 | Endogenous | 1513.36 | 481.92 | 2319.44 | 511.91 | 1834.91 | 586.14 |
| **PIN1** | NM_006221.2:434 | Endogenous | 868.81 | 156.23 | 874.72 | 79.46 | 938.62 | 103.11 |
| **PLA2G1B** | NM_000928.2:396 | Endogenous | 63.61 | 82.48 | 19.23 | 39.14 | 17.94 | 7.52 |
| **PLA2G6** | NM_001004426.1:1954 | Endogenous | 553.89 | 101.49 | 874.12 | 193.61 | 842.34 | 130.33 |
| **PLAU** | NM_002658.2:793 | Endogenous | 525.6 | 245.54 | 677.24 | 292.78 | 606.6 | 339.1 |
| **PLAUR** | NM_001005376.1:440 | Endogenous | 446.8 | 384.67 | 779.07 | 596.71 | 970.78 | 1060.26 |
| **PMCH** | NM_002674.2:172 | Endogenous | 77.74 | 169.67 | 34.02 | 72.28 | 17.47 | 7.38 |
| **PNMA1** | NM_006029.4:1565 | Endogenous | 642.41 | 97.24 | 767.22 | 72.96 | 822.39 | 152.68 |
| **POLR2A** | NM_000937.2:3775 | Housekeeping | 3574.85 | 880.57 | 5560.51 | 864.38 | 5827.89 | 2298.58 |
| **POS_A** | ERCC_00117.1:385 | Positive | 26057.56 | 10094.15 | 19158.9 | 3442.72 | 19838.2 | 3544.38 |
| **POS_B** | ERCC_00112.1:695 | Positive | 8951.72 | 4168.29 | 5789.53 | 1099.57 | 6029.48 | 1176.01 |
| **POS_C** | ERCC_00002.1:850 | Positive | 2490.78 | 1110.43 | 1627.82 | 314.67 | 1660.22 | 289.8 |
| **POS_D** | ERCC_00092.1:540 | Positive | 616.48 | 252.98 | 436.04 | 70.93 | 448.54 | 69.27 |
| **POS_E** | ERCC_00035.1:485 | Positive | 99.08 | 46.17 | 64.85 | 10.17 | 62.55 | 11.12 |
| **POS_F** | ERCC_00034.1:195 | Positive | 48.39 | 23.14 | 35.09 | 7.12 | 30.9 | 7.05 |
| **POU2AF1** | NM_006235.2:1675 | Endogenous | 1595.38 | 554.64 | 2372.45 | 375.58 | 1583.75 | 386.78 |
| **POU2F2** | NM_002698.2:908 | Endogenous | 1090.89 | 575.65 | 1811.55 | 631.38 | 1322.31 | 857.26 |
| **PPARG** | NM_015869.3:1035 | Endogenous | 816.64 | 358.36 | 652.1 | 320.35 | 1127.04 | 486.14 |
| **PPBP** | NM_002704.2:330 | Endogenous | 37.02 | 58.39 | 23.06 | 7.6 | 25.68 | 12.21 |
| **PPIA** | NM_021130.2:925 | Housekeeping | 1080.29 | 102.54 | 1258.14 | 110.06 | 1297.13 | 264.56 |
| **PRAME** | NM_006115.3:1390 | Endogenous | 105.1 | 237.28 | 59.17 | 42.6 | 41.84 | 16.84 |
| **PRF1** | NM_005041.3:2120 | Endogenous | 1000.71 | 453.26 | 997.68 | 391.78 | 872.49 | 176.82 |
| **PRG2** | NM_002728.4:256 | Endogenous | 96.89 | 151.59 | 50.7 | 44.85 | 37.87 | 18.81 |
| **PRKCD** | NM_006254.3:2165 | Endogenous | 2287.55 | 515.77 | 2642.79 | 270.72 | 2593.52 | 359.21 |
| **PRKCE** | NM_005400.2:1695 | Endogenous | 238.15 | 101.68 | 224.56 | 40.14 | 188.86 | 20.79 |
| **PRM1** | NM_002761.2:319 | Endogenous | 77.67 | 129.93 | 27.17 | 65.29 | 13.07 | 4.83 |
| **PRPF38A** | NM_032864.3:335 | Housekeeping | 2666.48 | 294.24 | 1899.15 | 178.03 | 1952.81 | 233.06 |
| **PSEN1** | NM_000021.2:900 | Endogenous | 2431.95 | 524.58 | 1672.54 | 344.81 | 1939.53 | 379.85 |
| **PSEN2** | NM_000447.2:915 | Endogenous | 681.33 | 117.18 | 532.74 | 134.76 | 611.08 | 117.79 |
| **PSMB10** | NM_002801.2:221 | Endogenous | 2196.79 | 563.75 | 3282.74 | 421.66 | 3093.09 | 843.89 |
| **PSMB7** | NM_002799.2:420 | Endogenous | 3748.15 | 668.85 | 3267.05 | 389.45 | 3177.92 | 411.76 |
| **PSMB8** | NM_004159.4:1215 | Endogenous | 5602.17 | 2229.61 | 8180.69 | 1207.63 | 7094 | 1498.06 |
| **PSMB9** | NM_002800.4:455 | Endogenous | 3495.75 | 1416.64 | 5314.51 | 1254.5 | 4285.07 | 1780.74 |
| **PSMD7** | NM_002811.3:580 | Endogenous | 3491.21 | 788 | 4261.27 | 522.98 | 4326.04 | 993.7 |
| **PTGDR2** | NM_004778.1:1835 | Endogenous | 283.73 | 203.15 | 153.69 | 136.83 | 218.57 | 193.77 |
| **PTGS2** | NM_000963.1:495 | Endogenous | 166.46 | 121.31 | 241.74 | 310.83 | 236.47 | 310.26 |
| **PTPRC** | NM_080921.3:258 | Endogenous | 4007.05 | 1808.69 | 3511.38 | 830.55 | 2990.74 | 1325.28 |
| **PVR** | NM_006505.3:604 | Endogenous | 621.35 | 307.75 | 592.91 | 180.45 | 804.67 | 276.24 |
| **PYCARD** | NM_013258.3:714 | Endogenous | 565.16 | 177.32 | 647.92 | 151.7 | 708.92 | 277.09 |
| **RAG1** | NM_000448.2:2300 | Endogenous | 99.16 | 176.7 | 34.98 | 34.14 | 28.97 | 10.52 |
| **REL** | NM_002908.2:225 | Endogenous | 1219.72 | 413.68 | 1819.15 | 460.69 | 1519.94 | 512.76 |
| **RELA** | NM_021975.2:360 | Endogenous | 457.93 | 92.37 | 725.02 | 65.07 | 753.27 | 100.11 |
| **RELB** | NM_006509.2:250 | Endogenous | 596.12 | 206.17 | 879.63 | 232.73 | 753.11 | 209.41 |
| **REPS1** | NM_001128617.2:1288 | Endogenous | 2225.8 | 588.59 | 1384.6 | 234.37 | 1497.42 | 349.28 |
| **RIPK2** | NM_003821.5:1695 | Endogenous | 587.88 | 105.96 | 588.91 | 114.5 | 485.48 | 88.53 |
| **ROPN1** | NM_017578.2:195 | Endogenous | 139.78 | 295.39 | 44.49 | 97.55 | 27.39 | 7.69 |
| **RORA** | NM_134261.2:1715 | Endogenous | 1140.34 | 345.25 | 1264.8 | 159.12 | 1352.91 | 259.38 |
| **RORC** | NM_001001523.1:1350 | Endogenous | 334.92 | 123.43 | 255.55 | 89.27 | 295.28 | 97.41 |
| **RPS6** | NM_001010.2:171 | Endogenous | 99335.48 | 18029.67 | 114076.8 | 22926.4 | 105169.23 | 23432.82 |
| **RRAD** | NM_004165.1:960 | Endogenous | 149.03 | 161.33 | 175.47 | 65.11 | 115.1 | 69.9 |
| **RUNX1** | NM_001754.4:635 | Endogenous | 851.36 | 171.49 | 1167.09 | 210.02 | 1322.96 | 463.29 |
| **RUNX3** | NM_004350.1:2085 | Endogenous | 1186.14 | 496.85 | 2994.6 | 790.91 | 2224.69 | 745.91 |
| **S100A12** | NM_005621.1:260 | Endogenous | 134.65 | 150.64 | 54.95 | 80.24 | 56.42 | 96.18 |
| **S100A7** | NM_002963.2:69 | Endogenous | 146.18 | 312.84 | 46.52 | 79.17 | 25.05 | 10.54 |
| **S100A8** | NM_002964.3:115 | Endogenous | 342.26 | 667.82 | 322.97 | 429.31 | 463.46 | 1457.59 |
| **S100B** | NM_006272.1:85 | Endogenous | 817.42 | 279.01 | 867.86 | 182.3 | 929.1 | 556.83 |
| **SAA1** | NM_199161.1:135 | Endogenous | 367.09 | 416.31 | 560.73 | 915.44 | 505.22 | 1707.8 |
| **SAP130** | NM_024545.3:3090 | Housekeeping | 1004.5 | 152.33 | 1016.17 | 55.62 | 986.63 | 135.48 |
| **SBNO2** | NM_014963.2:2002 | Endogenous | 1206.72 | 346.61 | 1822.87 | 431.86 | 1839.69 | 904.28 |
| **SDHA** | NM_004168.1:230 | Housekeeping | 2517.96 | 597.4 | 2253.58 | 514.17 | 2444.23 | 617.54 |
| **SELE** | NM_000450.2:1505 | Endogenous | 66.73 | 68 | 50.24 | 84.89 | 34.05 | 29.81 |
| **SELL** | NR_029467.1:1585 | Endogenous | 1619.98 | 1305.02 | 2973.24 | 1723.23 | 1860 | 1614.74 |
| **SELPLG** | NM_001206609.1:326 | Endogenous | 675.38 | 194.98 | 717.59 | 191.75 | 625.1 | 266.41 |
| **SEMG1** | NM_003007.2:1290 | Endogenous | 55.64 | 90.7 | 19.43 | 40.07 | 17.18 | 4.49 |
| **SERPINB2** | NM_002575.1:305 | Endogenous | 18.74 | 3.92 | 18.45 | 7.11 | 15.75 | 4.96 |
| **SERPING1** | NM_000062.2:305 | Endogenous | 4247.3 | 1124.77 | 3897.33 | 1060.08 | 3640.75 | 1238.58 |
| **SF3A3** | NM_006802.2:2060 | Housekeeping | 2213.14 | 336.57 | 3434.28 | 597.37 | 3075.85 | 661.85 |
| **SH2B2** | NM_020979.3:1567 | Endogenous | 321.63 | 112.2 | 610.4 | 222.11 | 419.89 | 126.61 |
| **SH2D1A** | NM_001114937.2:495 | Endogenous | 548.4 | 317.7 | 755.02 | 144.74 | 557.94 | 198.36 |
| **SH2D1B** | NM_053282.4:545 | Endogenous | 139.23 | 162.8 | 72.36 | 47.72 | 53.61 | 20.41 |
| **SIGIRR** | NM_021805.2:469 | Endogenous | 1240.21 | 162.15 | 1292.53 | 183.03 | 1521.62 | 314.48 |
| **SIGLEC1** | NM_023068.3:5165 | Endogenous | 760.82 | 285.93 | 764.77 | 323.81 | 915.37 | 396.72 |
| **SLAMF1** | NM_003037.2:580 | Endogenous | 352.8 | 197.03 | 538.48 | 173.83 | 435.49 | 186.57 |
| **SLAMF6** | NM_001184714.1:1032 | Endogenous | 597.9 | 345.21 | 1319.73 | 238.87 | 1041.35 | 590.9 |
| **SLAMF7** | NM_021181.3:215 | Endogenous | 1090.37 | 469.99 | 1475.42 | 681.21 | 1393.72 | 400.13 |
| **SLC11A1** | NM_000578.2:1965 | Endogenous | 96.87 | 127.5 | 52.75 | 84.42 | 53.17 | 36.89 |
| **SMAD2** | NM_005901.5:1678 | Endogenous | 2364.89 | 596.97 | 1411.88 | 248.08 | 1419.11 | 357.83 |
| **SMAD3** | NM_005902.3:4220 | Endogenous | 2194.75 | 633.34 | 3046.38 | 623.56 | 2935.84 | 438.4 |
| **SMPD3** | NM_018667.3:4733 | Endogenous | 260.89 | 121.48 | 216.98 | 86.37 | 271.31 | 82.03 |
| **SOCS1** | NM_003745.1:1025 | Endogenous | 1171.51 | 574.56 | 1039.64 | 262.78 | 721.67 | 428.25 |
| **SPA17** | NM_017425.3:175 | Endogenous | 589.89 | 218.88 | 320.84 | 138.2 | 324.92 | 96.59 |
| **SPACA3** | NM_173847.3:112 | Endogenous | 51.13 | 129.21 | 20.02 | 25.81 | 13.57 | 6.52 |
| **SPANXB1** | NM_032461.2:321 | Endogenous | 74.54 | 208.05 | 24.25 | 64.39 | 11.63 | 5.93 |
| **SPINK5** | NM_006846.3:2595 | Endogenous | 159.69 | 94.53 | 94.27 | 54.77 | 106.28 | 44.96 |
| **SPN** | NM_003123.3:2345 | Endogenous | 514.19 | 206.38 | 1130.04 | 386.67 | 1013.11 | 705.99 |
| **SPO11** | NM_198265.1:492 | Endogenous | 36.9 | 83.63 | 14.16 | 40.5 | 11.96 | 4.75 |
| **SPP1** | NM_000582.2:760 | Endogenous | 354.04 | 146.19 | 324.23 | 217.83 | 304.17 | 98.52 |
| **SSX1** | NM_005635.2:174 | Endogenous | 69.44 | 117.09 | 32.83 | 28.51 | 21.36 | 9.13 |
| **SSX4** | NM_005636.3:140 | Endogenous | 48.04 | 88.12 | 21.02 | 28.18 | 11.95 | 4.13 |
| **ST6GAL1** | NM_003032.2:1300 | Endogenous | 3662.21 | 972.83 | 4084.98 | 617.16 | 3423.43 | 962.21 |
| **STAT1** | NM_007315.2:205 | Endogenous | 4465.5 | 2283.09 | 4423.02 | 2025.85 | 3474.28 | 1901.04 |
| **STAT2** | NM_005419.2:1965 | Endogenous | 3284.35 | 546.7 | 3704.6 | 504.48 | 3698.13 | 692.5 |
| **STAT3** | NM_139276.2:4535 | Endogenous | 9924.27 | 2930.14 | 13553.34 | 1871.19 | 12818.82 | 1824.81 |
| **STAT4** | NM_003151.2:789 | Endogenous | 528.48 | 161.61 | 502.01 | 95.35 | 523.14 | 125.48 |
| **STAT5B** | NM_012448.3:200 | Endogenous | 1873.49 | 440.72 | 1878.56 | 154.25 | 1810.64 | 333.37 |
| **STAT6** | NM_003153.3:2030 | Endogenous | 5096.46 | 913.11 | 6085.09 | 880.33 | 6557.67 | 2452.78 |
| **SYCP1** | NM_003176.2:230 | Endogenous | 47.98 | 101.84 | 20.49 | 22.05 | 17.43 | 5.67 |
| **SYK** | NM_003177.3:1685 | Endogenous | 3071.59 | 907.04 | 4318.93 | 656.13 | 3206 | 865.51 |
| **SYT17** | NM_016524.2:1150 | Endogenous | 91.18 | 56.28 | 60.09 | 20.02 | 70.78 | 17.06 |
| **TAB1** | NM_153497.2:614 | Endogenous | 1014.6 | 159.51 | 1195.93 | 130.84 | 1233.95 | 123.53 |
| **TAL1** | NM_003189.2:4635 | Endogenous | 221.95 | 292.52 | 152.09 | 60.64 | 126.08 | 61.22 |
| **TANK** | NM_004180.2:110 | Endogenous | 3555.48 | 648.7 | 2985.24 | 344.33 | 2926.44 | 581.12 |
| **TAP1** | NM_000593.5:2075 | Endogenous | 1676.74 | 608.91 | 2353.44 | 525.77 | 1932.06 | 588.42 |
| **TAP2** | NM_000544.3:909 | Endogenous | 1451.66 | 406.37 | 2217.59 | 406.68 | 1934.27 | 639.47 |
| **TAPBP** | NM_003190.4:1536 | Endogenous | 7689.62 | 2005.11 | 10229.46 | 3010.26 | 10557.69 | 4666.98 |
| **TARP** | NM_001003799.1:560 | Endogenous | 836.18 | 509.34 | 645.81 | 295.74 | 724.92 | 215.03 |
| **TBK1** | NM_013254.2:1610 | Endogenous | 1073.28 | 163.57 | 1175.15 | 109.12 | 1089.14 | 154.97 |
| **TBP** | NM_001172085.1:587 | Housekeeping | 1118.54 | 160.59 | 801 | 124.95 | 841.62 | 133.94 |
| **TBX21** | NM_013351.1:890 | Endogenous | 267.65 | 177.61 | 240.32 | 68.11 | 201.33 | 49.87 |
| **TCF7** | NM_003202.2:2420 | Endogenous | 2052.19 | 1004.57 | 3311.72 | 1375.42 | 2946.47 | 1425.3 |
| **TFE3** | NM_006521.3:2935 | Endogenous | 219.68 | 53.65 | 305.5 | 84.78 | 288.53 | 84.14 |
| **TFEB** | NM_007162.2:2026 | Endogenous | 630.19 | 158.08 | 1364.56 | 312.71 | 1172.02 | 428.6 |
| **TFRC** | NM_003234.1:1220 | Endogenous | 3278.59 | 1175.36 | 3037.21 | 938.85 | 3137.72 | 673.35 |
| **TGFB1** | NM_000660.3:1260 | Endogenous | 2521.65 | 751.63 | 4182.89 | 942.63 | 3890.54 | 2629.96 |
| **TGFB2** | NM_003238.2:1125 | Endogenous | 96.6 | 21.21 | 91.81 | 24.96 | 88.08 | 67.42 |
| **THBD** | NM_000361.2:1246 | Endogenous | 528.24 | 188.99 | 531.06 | 172.83 | 521.63 | 147.16 |
| **THBS1** | NM_003246.2:3465 | Endogenous | 2224.88 | 2786.3 | 4756.09 | 5007.2 | 5110.78 | 5057.52 |
| **THY1** | NM_006288.2:135 | Endogenous | 928.44 | 267.93 | 1267.89 | 982.19 | 947.76 | 424 |
| **TICAM1** | NM_014261.1:518 | Endogenous | 634.13 | 235.03 | 750.35 | 103.32 | 822.7 | 247.26 |
| **TICAM2** | NM_021649.4:3234 | Endogenous | 576.82 | 178.71 | 461.67 | 117.38 | 377.93 | 102.03 |
| **TIGIT** | NM_173799.2:1968 | Endogenous | 651.27 | 358.83 | 1278.09 | 244.61 | 853.26 | 364.14 |
| **TIRAP** | NM_148910.2:661 | Endogenous | 291.71 | 89.49 | 472.4 | 45.79 | 556.48 | 120.01 |
| **TLK2** | NM_006852.2:2335 | Housekeeping | 1403.66 | 183.44 | 1490.46 | 69.67 | 1523.26 | 189.32 |
| **TLR1** | NM_003263.3:545 | Endogenous | 1882.53 | 693.45 | 1918.89 | 542.48 | 1697.31 | 540.15 |
| **TLR10** | NM_030956.2:2246 | Endogenous | 1508.94 | 951.93 | 1856.03 | 771.84 | 1357.08 | 870.3 |
| **TLR2** | NM_003264.3:180 | Endogenous | 474.73 | 122.97 | 460.82 | 90.06 | 429.09 | 188.67 |
| **TLR3** | NM_003265.2:230 | Endogenous | 457.6 | 174.82 | 214.03 | 134.71 | 234.69 | 147.82 |
| **TLR4** | NM_138554.2:2570 | Endogenous | 940.59 | 273.56 | 1358.41 | 286.76 | 1115.9 | 213.74 |
| **TLR5** | NM_003268.3:215 | Endogenous | 231.82 | 143.78 | 148.74 | 44.04 | 122.13 | 33.87 |
| **TLR6** | NM_006068.2:2530 | Endogenous | 1082.1 | 362.7 | 1272.94 | 327.48 | 981.85 | 313.04 |
| **TLR7** | NM_016562.3:4120 | Endogenous | 225.72 | 99.08 | 297.18 | 80.43 | 234 | 67.27 |
| **TLR8** | NM_016610.2:2310 | Endogenous | 224.4 | 122.55 | 154.48 | 71.14 | 119.57 | 78.67 |
| **TLR9** | NM_017442.2:985 | Endogenous | 307.16 | 167.64 | 411.1 | 157.36 | 326.87 | 184.52 |
| **TMEFF2** | NM_016192.2:972 | Endogenous | 119.25 | 100.33 | 66.46 | 33.88 | 51.18 | 58.23 |
| **TMUB2** | NM_024107.2:1485 | Housekeeping | 1179.9 | 207.2 | 1242.77 | 137.04 | 1380.26 | 183.22 |
| **TNF** | NM_000594.2:1010 | Endogenous | 429.62 | 338.35 | 571.3 | 393.28 | 535.93 | 416.26 |
| **TNFAIP3** | NM_006290.2:260 | Endogenous | 2665.42 | 794.28 | 4700.51 | 1727.03 | 3757.86 | 2072.61 |
| **TNFRSF10B** | NM_003842.3:565 | Endogenous | 621.25 | 191.13 | 567.9 | 72.48 | 609.39 | 134.94 |
| **TNFRSF10C** | NM_003841.3:682 | Endogenous | 161.24 | 89.4 | 105.45 | 60.2 | 94.23 | 70.77 |
| **TNFRSF11A** | NM_003839.2:490 | Endogenous | 885.26 | 324.31 | 720.55 | 374.37 | 871.49 | 387.26 |
| **TNFRSF11B** | NM_002546.2:1075 | Endogenous | 168.31 | 97.68 | 160.22 | 49.44 | 138.34 | 64.26 |
| **TNFRSF12A** | NM_016639.1:575 | Endogenous | 107.04 | 199.21 | 110.73 | 98.23 | 110.29 | 60.74 |
| **TNFRSF13B** | NM_012452.2:160 | Endogenous | 762.59 | 333.49 | 1087.77 | 228.85 | 1079.94 | 457.91 |
| **TNFRSF13C** | NM_052945.3:789 | Endogenous | 2075.59 | 1019.62 | 4633.04 | 1689.45 | 3106.74 | 1890.76 |
| **TNFRSF14** | NM_003820.2:916 | Endogenous | 2588.1 | 1089.48 | 4318.65 | 325.18 | 4311.85 | 520.5 |
| **TNFRSF17** | NM_001192.2:635 | Endogenous | 2786.63 | 970.01 | 2271.91 | 1062.02 | 1987.66 | 636.65 |
| **TNFRSF18** | NM_004195.2:445 | Endogenous | 347.8 | 168.15 | 508.36 | 240.16 | 473.61 | 262.86 |
| **TNFRSF1A** | NM_001065.2:515 | Endogenous | 2587.67 | 972.55 | 2307.71 | 309.1 | 2845.45 | 578.13 |
| **TNFRSF1B** | NM_001066.2:835 | Endogenous | 1104.38 | 587.33 | 2613.1 | 544.8 | 2238.59 | 1074.98 |
| **TNFRSF4** | NM_003327.2:200 | Endogenous | 195.26 | 65.91 | 322.35 | 124.93 | 220.87 | 150.43 |
| **TNFRSF8** | NM_152942.2:2030 | Endogenous | 173.28 | 139.12 | 207.87 | 146.18 | 114.12 | 99.15 |
| **TNFRSF9** | NM_001561.4:255 | Endogenous | 230.94 | 118.24 | 297.51 | 122.95 | 157.72 | 113.35 |
| **TNFSF10** | NM_003810.2:115 | Endogenous | 3394.62 | 1106.44 | 3472.69 | 1277.12 | 4021.9 | 998.46 |
| **TNFSF11** | NM_003701.2:490 | Endogenous | 187.64 | 162.77 | 363.2 | 197.38 | 243.4 | 185.13 |
| **TNFSF12** | NM_003809.2:339 | Endogenous | 1528.64 | 306.94 | 2152.02 | 234.05 | 2265.65 | 514.76 |
| **TNFSF13** | NM_003808.3:810 | Endogenous | 441.37 | 175.23 | 632.9 | 166.02 | 667.6 | 326.12 |
| **TNFSF13B** | NM_006573.4:1430 | Endogenous | 872.57 | 448.46 | 1037.37 | 461.2 | 694.74 | 193.54 |
| **TNFSF14** | NM_003807.3:612 | Endogenous | 268.65 | 100.25 | 434.81 | 135.5 | 367.75 | 238.65 |
| **TNFSF15** | NM_001204344.1:2338 | Endogenous | 331.44 | 298.77 | 383.36 | 131.32 | 300.3 | 77.11 |
| **TNFSF18** | NM_005092.2:175 | Endogenous | 24.23 | 14.53 | 19.23 | 9.33 | 18.27 | 13.89 |
| **TNFSF4** | NM_003326.2:545 | Endogenous | 316.8 | 183.69 | 214.59 | 56.93 | 188.4 | 61.27 |
| **TNFSF8** | NM_001244.3:518 | Endogenous | 451.3 | 260.37 | 364.92 | 111.71 | 296.17 | 327.8 |
| **TOLLIP** | NM_019009.2:1320 | Endogenous | 1645.39 | 551.81 | 1552.07 | 210.61 | 1548.78 | 342.67 |
| **TP53** | NM_000546.2:1330 | Endogenous | 2457.78 | 414.85 | 2577.08 | 214.13 | 2298.27 | 297.56 |
| **TPSAB1** | NM_003294.3:579 | Endogenous | 9070.98 | 4251.62 | 9983.44 | 2547.85 | 10113.85 | 4164.29 |
| **TPTE** | NM_199259.2:142 | Endogenous | 65.02 | 255.35 | 26.43 | 44.06 | 15.13 | 3.47 |
| **TRAF2** | NM_021138.3:1325 | Endogenous | 1171.15 | 265.27 | 1299.58 | 161.25 | 1244.97 | 318.22 |
| **TRAF3** | NM_145725.1:1795 | Endogenous | 1616.01 | 311.44 | 1942.99 | 352.49 | 1757.95 | 397.04 |
| **TRAF6** | NM_145803.1:1839 | Endogenous | 1125.68 | 363.26 | 955.1 | 102.29 | 1002.02 | 121.08 |
| **TREM1** | NM_018643.3:375 | Endogenous | 66.32 | 176.19 | 37.2 | 83.96 | 30.09 | 67.67 |
| **TREM2** | NM_018965.3:611 | Endogenous | 71.8 | 128.93 | 33.94 | 48.91 | 29.28 | 8.36 |
| **TRIM39** | NM_021253.3:3140 | Housekeeping | 456.96 | 137.43 | 418.86 | 57.75 | 371.68 | 39.91 |
| **TTK** | NM_003318.3:1200 | Endogenous | 442.4 | 134.43 | 298.81 | 101.7 | 214.45 | 91.36 |
| **TUBB** | NM_178014.2:320 | Housekeeping | 7694.76 | 1658.13 | 8702.34 | 1046.84 | 7140.66 | 1851.81 |
| **TXK** | NM_003328.1:800 | Endogenous | 231.79 | 116.59 | 269.22 | 108.1 | 293.42 | 180.02 |
| **TXNIP** | NM_006472.1:255 | Endogenous | 30578.64 | 11620.06 | 43059.41 | 26410.72 | 46992.24 | 27731.45 |
| **TYK2** | NM_003331.3:485 | Endogenous | 2080.94 | 491.94 | 2071.58 | 340.82 | 1856.85 | 314.93 |
| **UBC** | NM_021009.3:1875 | Endogenous | 57811.87 | 16372.99 | 68179.71 | 13414.19 | 69732.25 | 19296.42 |
| **ULBP2** | NM_025217.2:905 | Endogenous | 141.28 | 452.73 | 35.7 | 116.87 | 19.57 | 9.48 |
| **USP39** | NM_001256725.1:806 | Housekeeping | 771.47 | 79.44 | 523.29 | 44.29 | 521.77 | 96.45 |
| **USP9Y** | NM_004654.3:85 | Endogenous | 138.64 | 266.69 | 43.05 | 308.31 | 44.23 | 304.15 |
| **VCAM1** | NM_001078.3:2535 | Endogenous | 2305.22 | 1097.97 | 2891.65 | 880.48 | 1955.65 | 674.37 |
| **VEGFA** | NM_001025366.1:1325 | Endogenous | 1267.45 | 475.97 | 1258.77 | 785.62 | 1405.57 | 397.39 |
| **VEGFC** | NM_005429.2:565 | Endogenous | 414.17 | 156.8 | 368.44 | 93.11 | 329.64 | 84.49 |
| **XCL2** | NM_003175.3:377 | Endogenous | 428.95 | 289.53 | 180.6 | 124.79 | 158.89 | 66.21 |
| **XCR1** | NM_005283.2:700 | Endogenous | 204.59 | 213.19 | 184.95 | 73.94 | 154.9 | 60.45 |
| **YTHDF2** | NM_001172828.1:275 | Endogenous | 1617.7 | 187.34 | 1206.92 | 123.89 | 1178.75 | 233.12 |
| **ZAP70** | NM_001079.3:1175 | Endogenous | 1274.68 | 553.58 | 1920.46 | 647.62 | 1901.53 | 1354.49 |
| **ZC3H14** | NM_001160103.1:2690 | Housekeeping | 1690.22 | 258.67 | 1695.46 | 312.22 | 1658.93 | 745.75 |
| **ZKSCAN5** | NM_014569.3:3688 | Housekeeping | 207.65 | 38.79 | 208.74 | 35.28 | 188.81 | 34.79 |
| **ZNF143** | NM_003442.5:925 | Housekeeping | 861.14 | 148.62 | 738.48 | 86.11 | 715.1 | 101.86 |
| **ZNF205** | NM_001031686.1:573 | Endogenous | 96.84 | 24.37 | 123.13 | 33.34 | 142.75 | 34.43 |
| **ZNF346** | NM_012279.2:2260 | Housekeeping | 453.65 | 174.24 | 434.92 | 109.29 | 397.9 | 75.41 |

StDev=standard deviation.

**Supplementary Table 4.** Overview of VA patient samples included in the different analyses.

| **Study ID** | **Disease** | **FFPE mRNA expression** | **Frozen MC Flow Cytometry** | **Appendix microbiome sequencing** | **Sex** | **Age [years]** | **Diabetic** |
| --- | --- | --- | --- | --- | --- | --- | --- |
| APP-001 | Local PDAC | y |  | y | M | 83 | y |
| APP-002 | Local PDAC | y | y | y | M | 64 | n |
| APP-003 | Metastatic PDAC | y | y | y | M | 59 | y |
| APP-004 | Local PDAC | y | y | y | M | 65 | n |
| APP-006 | Local PDAC | y | y | y | F | 72 | y |
| APP-007 | Local PDAC | y |  |  | M | 72 | y |
| APP-008 | Metastatic PDAC |  | y |  | F | 65 | y |
| APP-009 | Local PDAC | y |  | y | M | 89 | n |
| APP-011 | Local PDAC | y | y | y | F | 59 | n |
| APP-012 | Local PDAC |  |  |  | F | 72 | y |
| APP-013 | Local PDAC | y |  | y | F | 64 | n |
| APP-014 | Local PDAC | y |  |  | F | 82 | n |
| APP-017 | Metastatic PDAC | y | y | y | F | 75 | n |
| APP-018 | Local PDAC | y |  | y | M | 61 | y |
| APP-019 | Local PDAC | y | y | y | M | 59 | y |
| APP-020 | Local PDAC | y | y | y | M | 68 | y |
| APP-021 | Local PDAC | y | y | y | F | 49 | y |
| APP-022 | Local PDAC | y | y | y | M | 71 | n |
| VASana45 | Local colon AC | y |  |  | F | 68 | uk |
| VASana16 | Metastatic colon AC | y |  |  | F | 58 | uk |
| VASana44 | Local colon AC | y |  |  | M | 76 | uk |
| VASana18 | Metastatic colon AC | y |  |  | M | 67 | uk |
| VASana14 | Local colon AC | y |  |  | F | 52 | uk |
| VASana49 | Local colon AC | y |  |  | M | 57 | uk |
| VASana42 | Local colon AC | y |  |  | F | 72 | uk |
| VASana19 | Local colon AC | y |  |  | F | 59 | uk |
| VASana46 | Metastatic colon AC | y |  |  | F | 53 | uk |
| c-101 | Local Colon AC |  | y | y | M | 57 | n |
| c-102 | Metastatic Colon AC |  | y | y | F | 58 | n |
| c-103 | Local Colon AC |  | y | y | F | 66 | n |
| c-104 | Local Colon AC |  | y | y | F | 80 | y |
| c-105 | Local Colon AC |  | y | y | M | 71 | n |
| c-107 | Local Colon AC |  | y | y | F | 90 | n |
| c-108 | Local Colon AC |  | y | y | M | 77 | n |
| c-109 | Local Colon AC |  | y | y | F | 67 | n |
| c-110 | Local Colon AC |  | y | y | F | 79 | y |
| c-111 | Local Colon AC |  | y | y | F | 75 | n |
| c-112 | Local Colon AC |  |  | y | F | 69 | n |
| c-113 | Local Colon AC |  | y | y | M | 59 | n |
| c-114 | Local Colon AC |  | y |  | M | 71 | n |
| c-201 | Local Colon AC |  |  | y | F | 68 | y |
| VASana34 | Benign – Ileus | y |  |  | F | 95 | uk |
| VASana36 | Benign –slow transit obstipation | y |  |  | M | 53 | uk |
| VASana35 | Benign – megacolon | y |  |  | M | 55 | uk |
| VASana38 | Benign –slow transit obstipation | y |  |  | F | 43 | uk |
| VASana32 | Benign – diverticulitis | y |  |  | F | 71 | uk |
| VASana37 | Benign – chronic obstipation | y |  |  | F | 48 | uk |
| VASana22 | Benign – appendicitis suspected, no disease detected by histology. | y |  |  | M | 43 | uk |
| VASana27 | Benign – appendicitis suspected, no disease detected by histology. | y |  |  | M | 55 | uk |
| VASana28 | Benign – Mesothelial cyst | y |  |  | F | 63 | uk |

VA Sana= vermiform appendix sana; FFPE=formalin fixed paraffin embedded; MC=mononuclear cells; PDAC=pancreatic ductal adenocarcinoma; y=yes; M=male; F=female; n=no; uk=unknown.

# Supplementary Figure Legends

**Supplementary Figure 1. Visualization of HLA-G protein expression by immunohistochemistry. (A).** Representative image of human placenta FFPE tissue slide, stained with hematoxylin (blue, nuclei) and an antibody against HLA-G (visualized with DAB in brown). Scale bar = 100 µm. **(B)** Representative images of human adult VA samples from three different patient groups. AC=adenocarcinoma; PDAC=pancreatic ductal adenocarcinoma. Tissue slides are stained with hematoxylin (blue, nuclei) and an antibody against HLA-G (visualized with DAB in brown). Scale bars = 250 µm. (C) Bar graphs showing median DAB intensity (HLA-G) in the stained VA tissue slides. Quantification was performed using images containing ≥1 germinal center regions from ≥6 patients per group (equal area sizes). Bars show median intensity, each dot represents one image of one patient, error bars are 95% CI.

**Supplementary Figure 2. Visualization of HLA-G protein expression by multiplex immunofluorescence. (A)** Fluorescence images of a human placenta FFPE tissue slide, stained with antibodies against HLA-G (yellow), pan cytokeratin (green), CD79A (red, B cells), and DAPI (navy blue, nuclei). Scale bars = 100 µm. **(B)** Representative fluorescence images of human VA FFPE tissue slides, stained with antibodies against CD68 (aqua blue, macrophages), CD8 (green), CD4 (white), HLA-G (yellow), CD79A (red, B cells), and DAPI (navy blue, nuclei). Scale bars = 100 µm

**Supplementary Figure 3. Imaging Mass Cytometry (IMC) analysis of the adult VA immune microenvironment in three patient groups. (A)** Heatmap showing relative immune cell abundance in mantle zones of VA GALT (*n*=3 patient samples per disease group), based on 36-marker panel. DC=dendritic cells; M=macrophages; h.e.v.=high endothelial venules, PDAC=pancreatic ductal adenocarcinoma; AC=adenocarcinoma; col=colon. **(B)** Heatmap showing the percentage of HLA-G+ cells in each immune cell type in multiple regions of interest (ROI) per patient sample.

**Supplementary Figure 4. Flow cytometry analyses of VA mononuclear cells from PDAC and Colon AC patients.** **(A)** Representative biaxial plots showing the gating strategy used to identify mononuclear cell subsets in adult human vermiform appendix samples. SSA=side scatter; FSC=forward scatter; CD=cluster of differentiation; DN=double negative; NK=natural killer; Ig=immunoglobulin.

**Supplementary Figure 5. Flow cytometry analyses of expression markers on VA mononuclear cells from PDAC and Colon AC patients. (A)** Percentage of immunoglobulin (Ig) expressing memory B cells in the two patient groups. AC=adenocarcinoma; PDAC=pancreatic ductal adenocarcinoma. **(B)** Percentage of VA mononuclear cells positive for HLA-G surface staining per patient group. Transt=transitional B cell; mem=memory B cell  **(C)** Percentage of activation marker positive B cell subsets in the two patient groups. *=*P*<0.050. AC=adenocarcinoma; PDAC=pancreatic ductal adenocarcinoma. Bars show median percentages, each dot represents an individual patient sample, error bars are 95% CI, *P*-values by Mann–Whitney U test.

**Supplementary Figure 6. Fecal microbiome composition in VA of PDAC and Colon AC patients. (A)** Number of DNA sequencing reads obtained per VA fecal sample (*n*=13 colon AC, *n*=14 PDAC). Represented as median, error bars = 95% CI and each dot represents an individual patient sample. *P*-values by Mann–Whitney U test. AC=adenocarcinoma; PDAC=pancreatic ductal adenocarcinoma. **(B)** Number of bacterial hits per patient sample using the CosmosIDHub Bacteria database version 2.0.1. Grey area indicates the cut-off of <50 bacterial hits from which patient samples are excluded from further relative abundance analyses. **(C)** Relative bacterial class abundance in the two patient groups. *P*-value by two-way ANOVA. **(D)** Linear discriminant analysis effect size (LEfSe) analysis of virulence factors in the two patient groups. Cut-off linear Discriminant analysis (LDA) score ≥3 and *P*<0.05 by Kruskal-Wallis sum-rank test.

**
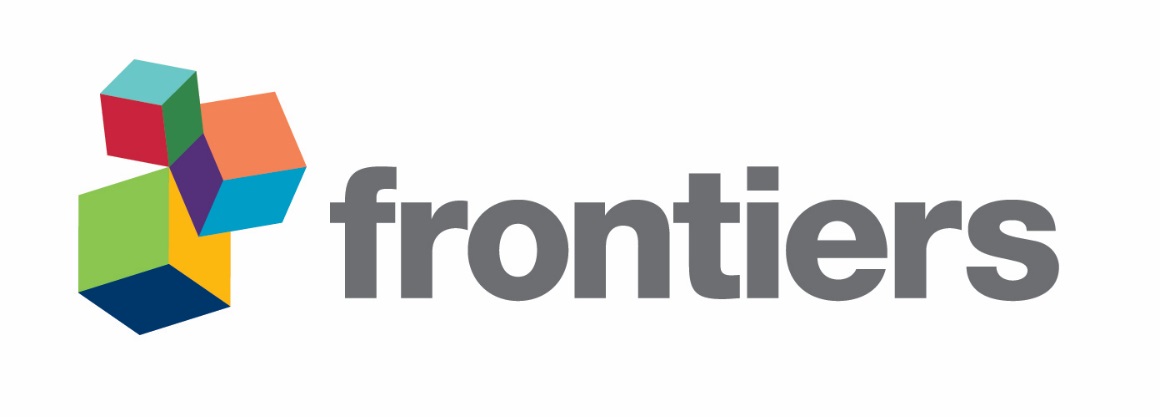
**
