# Supplementary figures and images for: B cell immune profiles in dysbiotic vermiform appendixes of pancreatic cancer patients
Source: Front Immunol. 2023 Nov 10;14:1230306. doi: 10.3389/fimmu.2023.1230306 (PMC10667699; doi:10.3389/fimmu.2023.1230306)

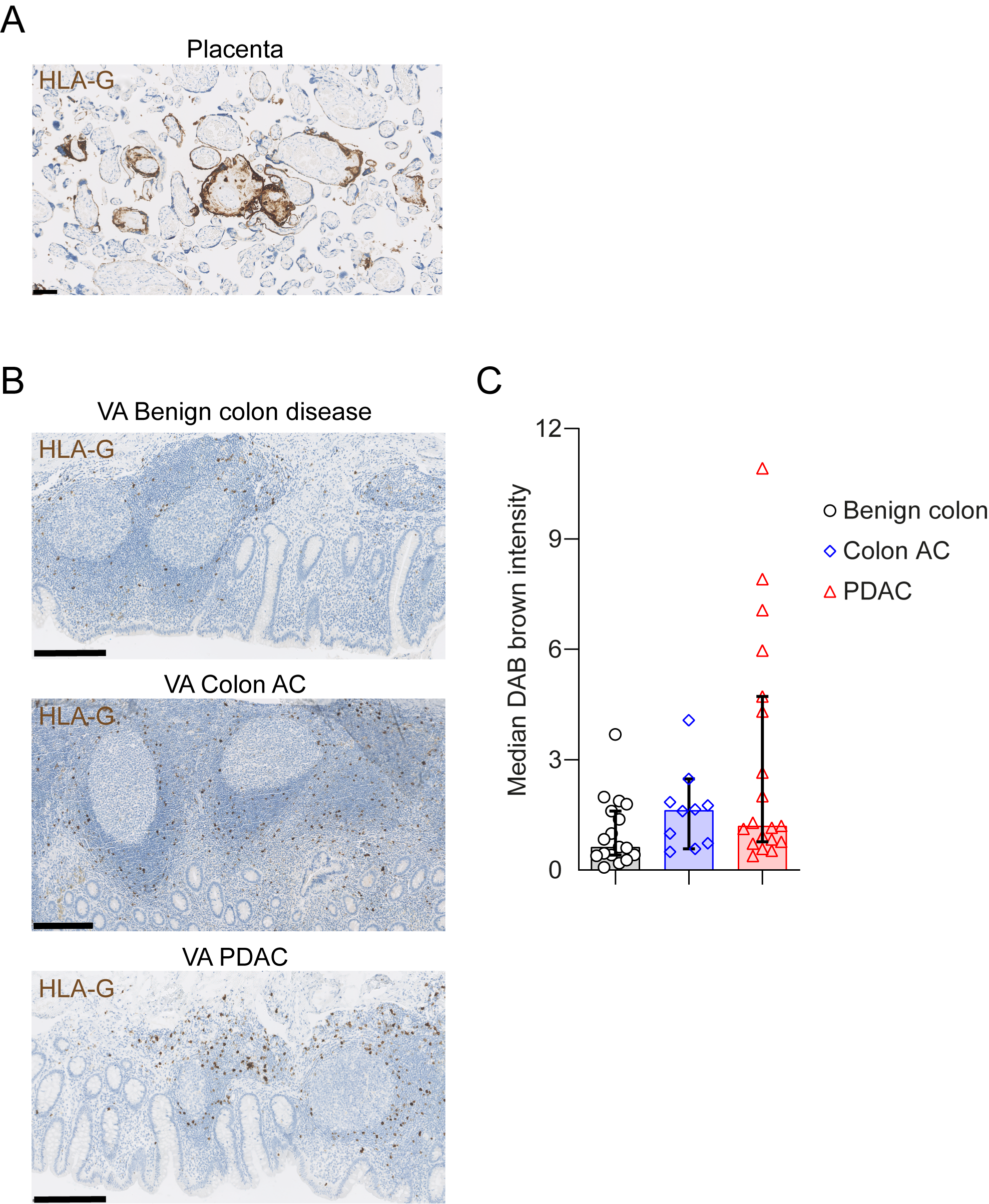

Supplement: Supplementary file 2 [file Image_1.tif]

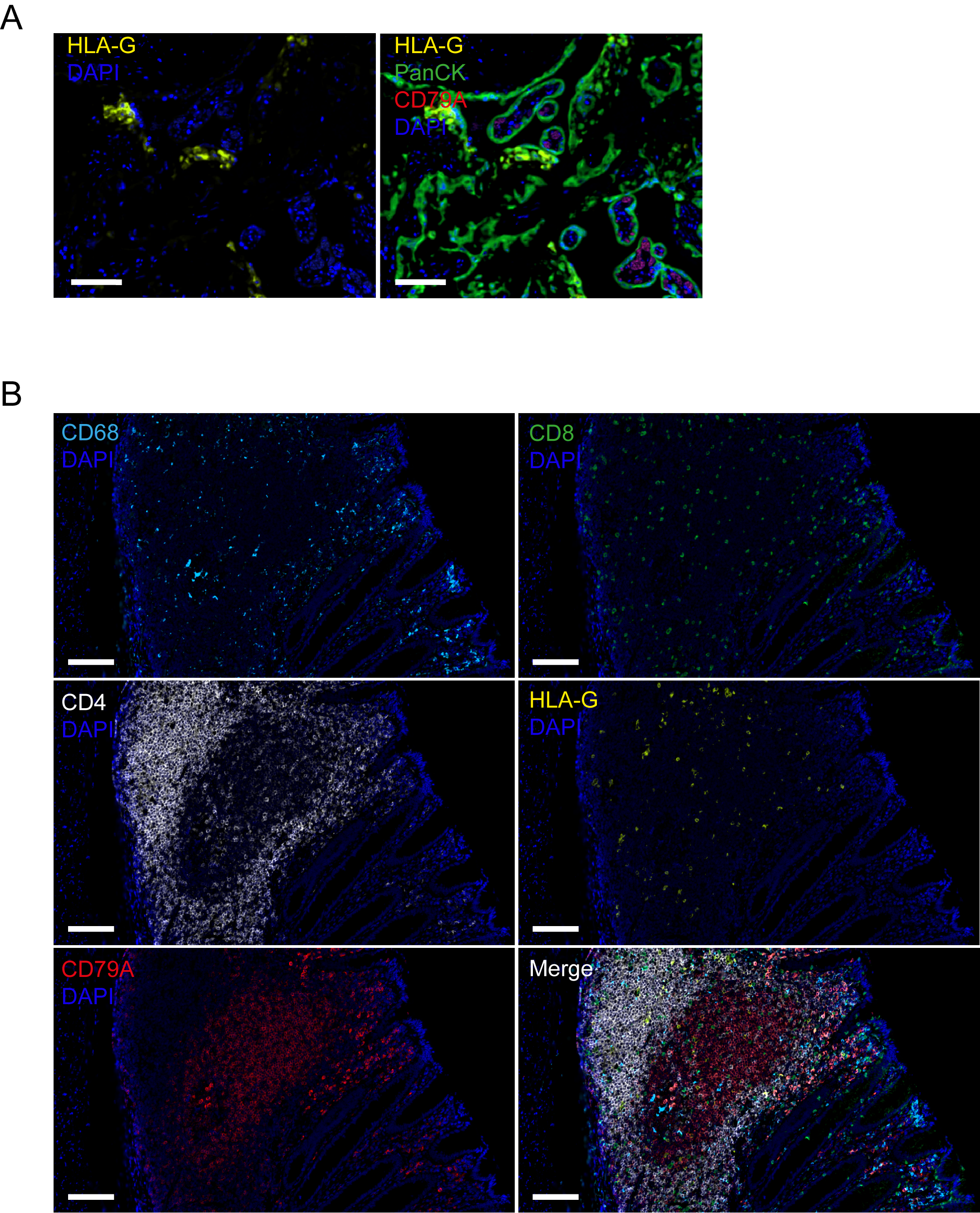

Supplement: Supplementary file 3 [file Image_2.tif]

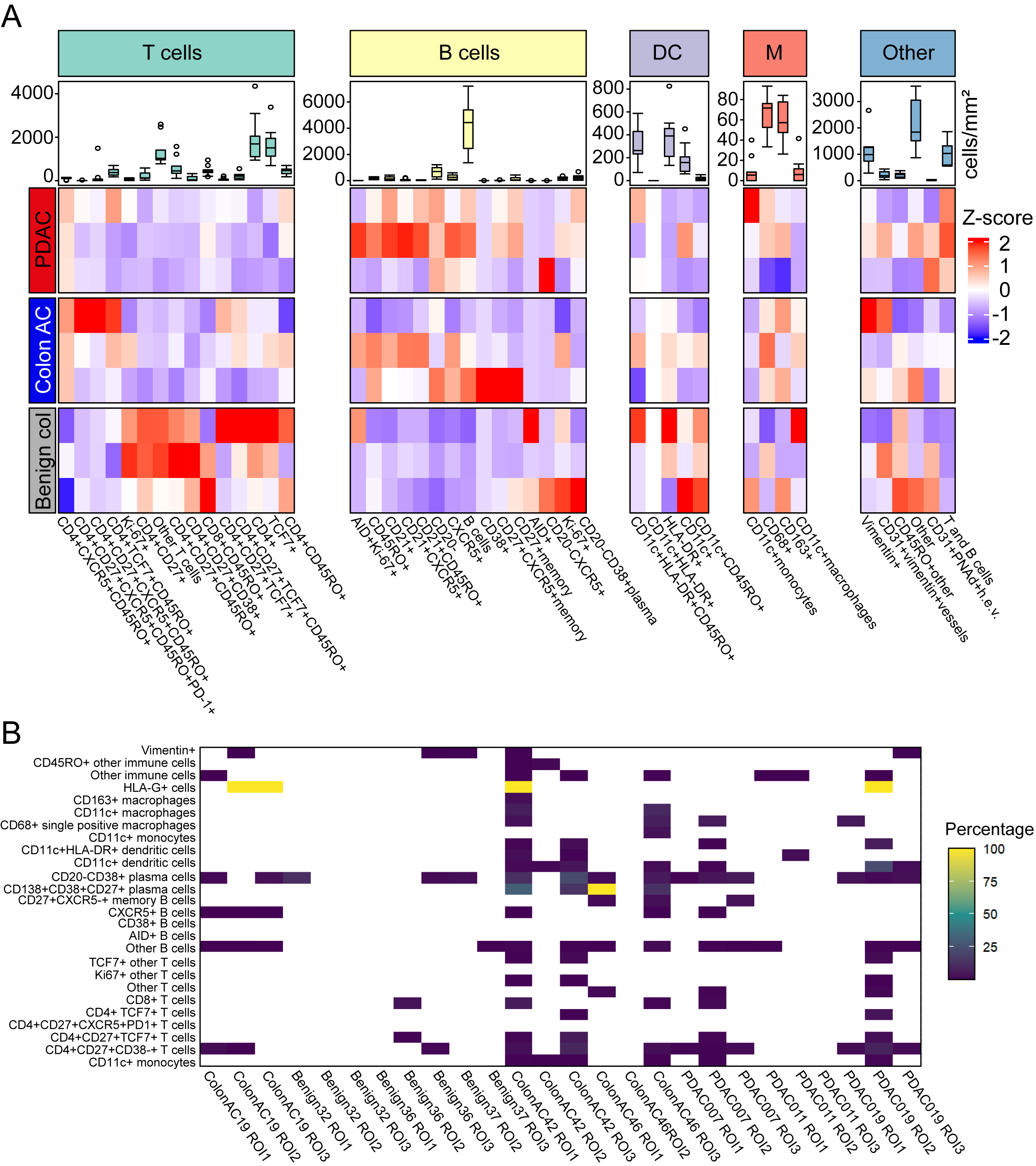

Supplement: Supplementary file 4 [file Image_3.tif]

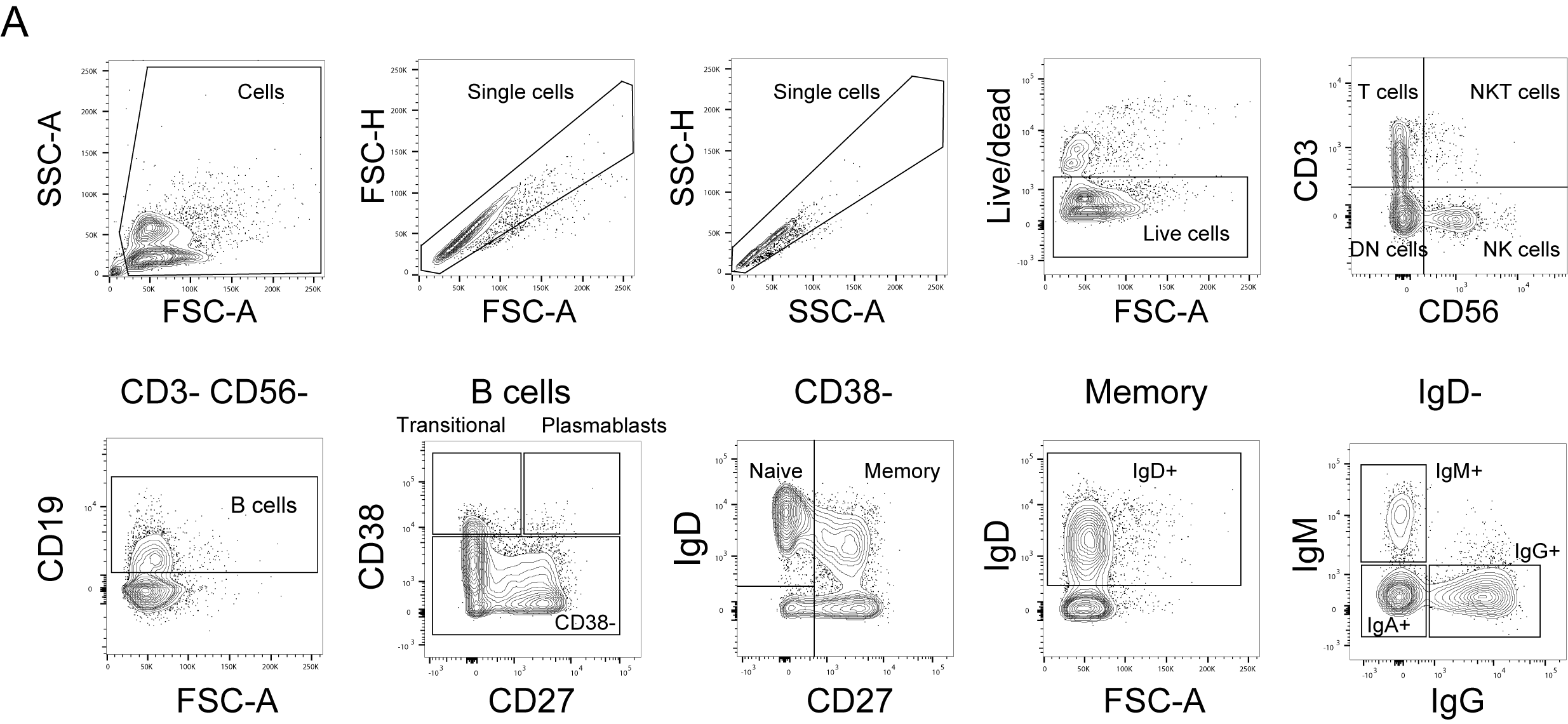

Supplement: Supplementary file 5 [file Image_4.tif]

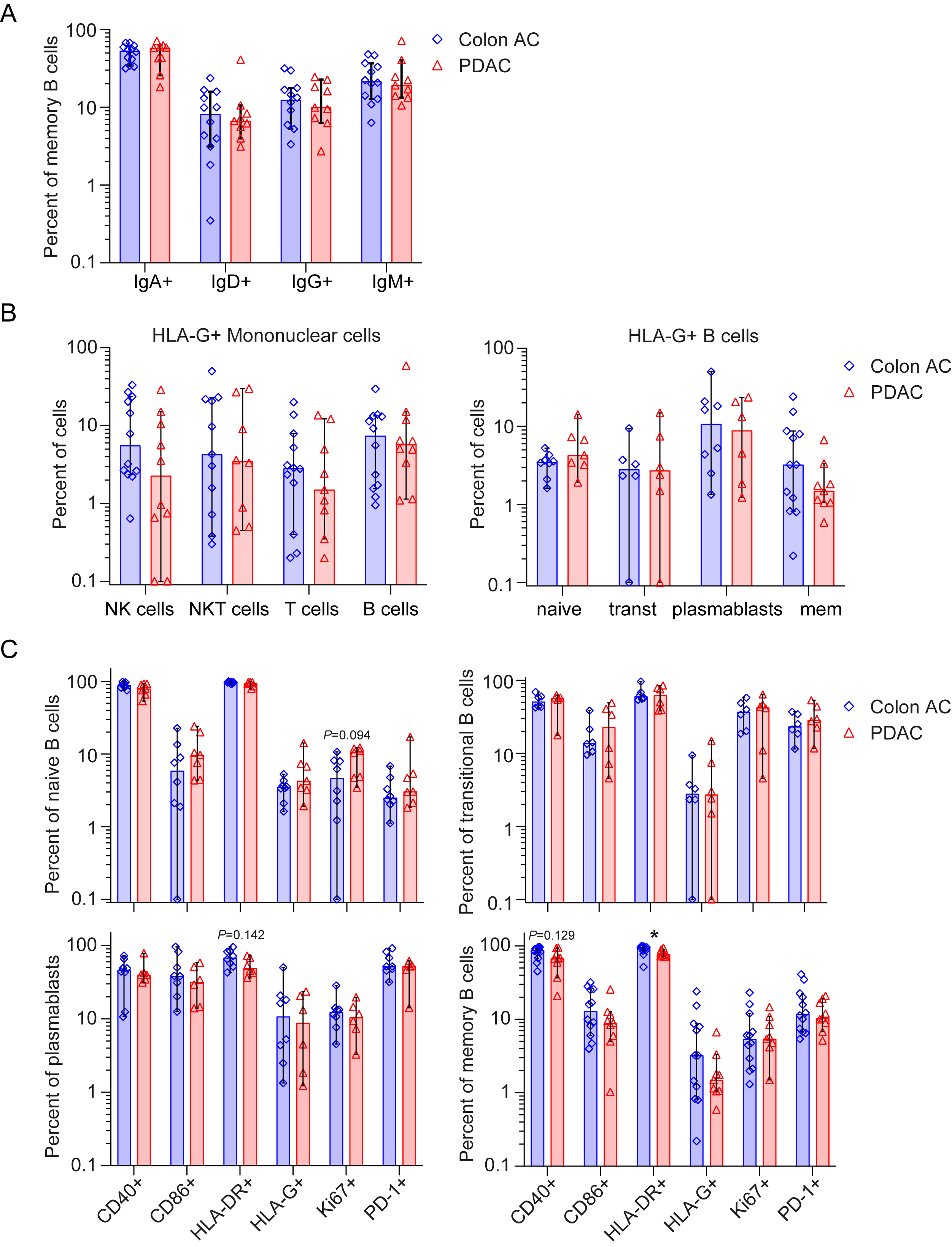

Supplement: Supplementary file 6 [file Image_5.tif]

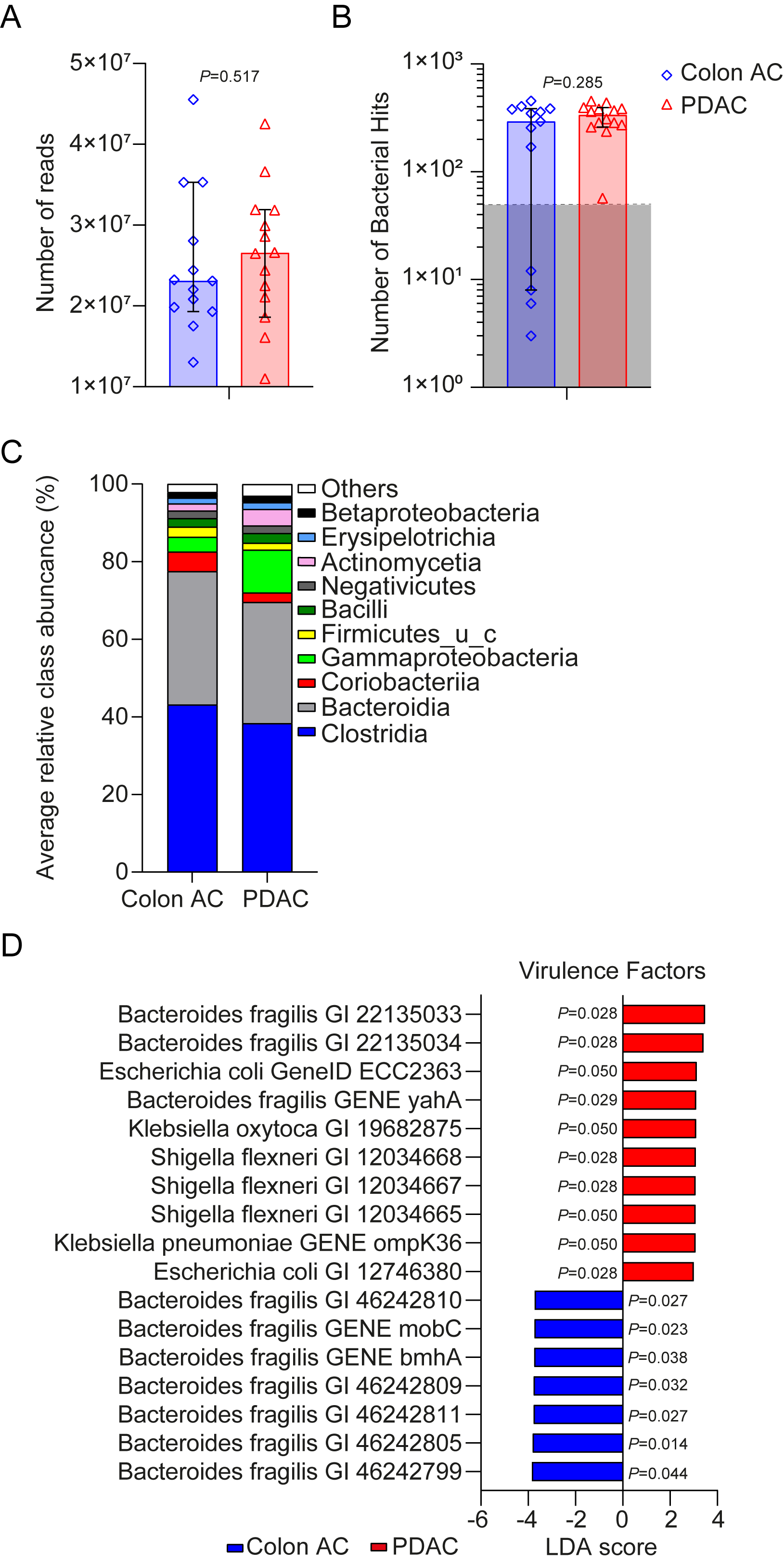

Supplement: Supplementary file 7 [file Image_6.tif]
